# Health effects associated with exposure to intimate partner violence against women and childhood sexual abuse: a Burden of Proof study

**DOI:** 10.1038/s41591-023-02629-5

**Published:** 2023-12-11

**Authors:** Cory N. Spencer, Mariam Khalil, Molly Herbert, Aleksandr Y. Aravkin, Alejandra Arrieta, María Jose Baeza, Flavia Bustreo, Jack Cagney, Renzo J. C. Calderon-Anyosa, Sinclair Carr, Jaidev Kaur Chandan, Carolina V. N. Coll, Fabiana Martins Dias de Andrade, Gisele N. de Andrade, Alexandra N. Debure, Luisa S. Flor, Ben Hammond, Simon I. Hay, Felicia N. Knaul, Rachel Q. H. Lim, Susan A. McLaughlin, Sonica Minhas, Jasleen K. Mohr, Erin C. Mullany, Christopher J. L. Murray, Erin M. O’Connell, Vedavati Patwardhan, Sofia Reinach, Dalton Scott, Reed J. D. Sorenson, Caroline Stein, Heidi Stöckl, Aisha Twalibu, Nádia Vasconcelos, Peng Zheng, Nicholas Metheny, Joht Singh Chandan, Emmanuela Gakidou

**Affiliations:** 1 Institute for Health Metrics and Evaluation, University of Washington, Seattle, WA USA; 2 Department of Applied Mathematics, University of Washington, Seattle, WA USA; 3 Department of Health Metrics Sciences, School of Medicine, University of Washington, Seattle, WA USA; 4 School of Medicine, The Pontifical Catholic University of Chile, Santiago, Chile; 5 School of Nursing and Health Studies, University of Miami, Coral Gables, FL USA; 6 Fondation Botnar, Basel, Switzerland; 7 Partnership for Maternal, Newborn and Child Health, Geneva, Switzerland; 8 McGill University, Montreal, Quebec Canada; 9 Warwick Medical School, University of Warwick, Coventry, UK; 10 Institute of Applied Health Research, University of Birmingham, Birmingham, UK; 11 Department of Epidemiology, Federal University of Pelotas, Pelotas, Brazil; 12 Human Development and Violence Research Center, Federal University of Pelotas, Pelotas, Brazil; 13 Federal University of Minas Gerais, Belo Horizonte, Brazil; 14 Institute for the Advanced Study of the Americas, University of Miami, Coral Gables, FL USA; 15 Center on Gender Equity and Health, UC San Diego School of Medicine, San Diego, CA USA; 16 Vital Strategies, New York, NY USA; 17 Institute of Medical Information Processing, Biometry and Epidemiology (IBE), Ludwig-Maximilians-University Munich, Munich, Germany

**Keywords:** Risk factors, Trauma

## Abstract

The health impacts of intimate partner violence against women and childhood sexual abuse are not fully understood. Here we conducted a systematic review by comprehensively searching seven electronic databases for literature on intimate partner violence-associated and childhood sexual abuse-associated health effects. Following the burden of proof methodology, we evaluated the evidence strength linking intimate partner violence and/or childhood sexual abuse to health outcomes supported by at least three studies. Results indicated a moderate association of intimate partner violence with major depressive disorder and with maternal abortion and miscarriage (63% and 35% increased risk, respectively). HIV/AIDS, anxiety disorders and self-harm exhibited weak associations with intimate partner violence. Fifteen outcomes were evaluated for their relationship to childhood sexual abuse, which was shown to be moderately associated with alcohol use disorders and with self-harm (45% and 35% increased risk, respectively). Associations between childhood sexual abuse and 11 additional health outcomes, such as asthma and type 2 diabetes mellitus, were found to be weak. Although our understanding remains limited by data scarcity, these health impacts are larger in magnitude and more extensive than previously reported. Renewed efforts on violence prevention and evidence-based approaches that promote healing and ensure access to care are necessary.

## Abstract (web summary)

A meta-analysis using the burden of proof risk function identified consistent evidence supporting harmful associations between the exposure to intimate partner violence against women and childhood sexual abuse on health outcomes.

## Main

Violence against women, gender-based violence and violence against children are global health priorities and unacceptably pervasive human rights violations^1–3^. Intimate partner violence against women and childhood sexual abuse are two of the most prevalent and pernicious forms of violence, associated with substantial morbidity and mortality^4–8^. Globally, it is estimated that almost one in three ever-partnered women have experienced physical and/or sexual intimate partner violence in their lifetime and 20% of young women and almost 10% of young men have experienced some form of childhood sexual abuse^4,9^. Intimate partner violence is defined as any lifetime experience of physical or sexual violence perpetrated against women by a current or former intimate partner and childhood sexual abuse is defined as exposure of women and men before age 15 to any unwanted sexual contact^10^. Existing work points to the wide extent of associated serious health consequences. The immediate emotional and physical trauma of intimate partner violence and childhood sexual abuse too often leads to mental and other health consequences that can reverberate across lifetimes and over generations^5,6,11–14^. Although long treated separately, it is now established that intimate partner violence and childhood sexual abuse co-occur in the same families, with shared risk and protective factors and produce compounding consequences^15–17^ across the lifespan. For example, childhood sexual abuse is an acknowledged risk factor for later experience and perpetration of intimate partner violence^18^.

Intimate partner violence and childhood sexual abuse have been included within the Global Burden of Diseases, Injuries and Risk Factors (GBD) risk assessment framework since 2010. Intimate partner violence accounts for more disability-adjusted life years in women of reproductive ages than risk factors such as smoking^19,20^. Currently, the attributable health burden of both intimate partner violence and childhood sexual abuse are calculated in relation to relatively few health outcomes, likely underestimating their negative impacts. Nevertheless, the latest iteration of the GBD suggests that women exposed to intimate partner violence are 1.54 times as likely to experience depression and 1.60 times as likely to become infected with HIV, while individuals exposed to childhood sexual abuse are 2.21 times as likely to experience alcohol use disorder and 1.56 times as likely to experience depression^10^. There is an urgent need to update our understanding of the health burdens associated with both risk factors with the most recently available evidence.

In this study, we assessed all available literature on the health impacts of intimate partner violence against women and childhood sexual abuse by systematically searching seven databases for evidence on all forms of violence against women and children. Here, we constrained this larger dataset to evaluate health risks associated with existing GBD risk factors: intimate partner violence and childhood sexual abuse. We re-examine existing risk–outcome pairs in the GBD and evaluate the strength of evidence for new pairs, following the burden of proof risk function (BPRF) methodology developed by Zheng and colleagues^21^. Elucidating the consequences of violence against women and children is key to centering them as a global health priority and motivating investment in prevention and effective, multi-pronged support to survivors. Outcomes found to be substantially associated with either risk factor suggest areas of intervention to prevent and manage negative health consequences, whereas associations with weaker evidence highlight opportunities for further research (Table 1).

**Table 1:** Policy summary

Background	Intimate partner violence and childhood sexual abuse are unacceptably pervasive violations of human rights and are risk factors for subsequent disease and disability that can reverberate across lifetimes and across generations. Research investigating relationships of intimate partner violence and childhood sexual abuse to specified health outcomes has been limited, underestimating their extensive health and societal impacts.
Main findings and limitations	Based on our systematic review and meta-analysis incorporating between-study heterogeneity to generate conservative estimates of the relationships between intimate partner violence or childhood sexual abuse and selected health outcomes, we found moderate evidence linking intimate partner violence to major depressive disorder and to maternal abortion and miscarriage, with our results indicating that intimate partner violence exposure was associated with, respectively, at least a 63% and a 35% increase in risk of these two outcomes occurring. On a scale with zero stars representing no evidence of association and five stars representing strong evidence, these relationships received three-star ratings. We further found weak evidence, based on the available data, for associations between intimate partner violence and HIV/AIDS (two-star rating), anxiety disorders (one star) and self-harm (one star). Extending this conservative meta-analytic framework incorporating between-study heterogeneity to estimation of childhood sexual abuse effects, we found that childhood sexual abuse exposure increased the risk of alcohol use disorders by at least 45% and risk of self-harm by 35%, with both relationships receiving a three-star rating. We also found that the existing evidence weakly supports associations between childhood sexual abuse and major depressive disorder (two stars), anxiety disorders (two stars), asthma (two stars), diabetes (one star), HIV/AIDS (one star), maternal abortion and miscarriage (one star), sexually transmitted infections excluding HIV (one star), drug use disorders (one star), conduct disorder (one star), bulimia nervosa (one star) and schizophrenia (one star). We also explored the association between childhood sexual abuse and anorexia nervosa and ischemic heart disease and found that the available evidence is not strong enough to support an association. Limitations of this study include the considerable variation across input studies in the way intimate partner violence and childhood sexual abuse are defined and measured and the extent to which potential confounding variables are controlled for. Although we accounted for study-level variability to the degree possible by including relevant covariates in the model, it would be useful in future research to better characterize the impact of confounding variables and to disaggregate definitions and measurements to achieve a more granular understanding of how particular forms of violence differentially affect health outcomes. An additional limitation is that, based on the input data available, it was necessary to model intimate partner violence and childhood sexual abuse as dichotomous risks, likely obscuring crucial information about timing, accumulation and frequency of exposure. Future research evaluating dose–response relationships will provide essential details about the effects of experiencing more than one type of violence and/or violence at multiple points during an individual’s lifetime.
Policy implications	Our review extends the previous evidence base surrounding the wide-ranging health impacts of intimate partner violence and childhood sexual abuse, which affect not only individuals but entire societies and economies. Results highlight the need to improve detection of violence against women and children. Promoting education around forms of violence and encouraging trauma-informed screening in healthcare settings are effective approaches to reach those experiencing intimate partner violence and childhood sexual abuse. Intimate partner violence and childhood sexual abuse are likely to co-occur in the same households with shared risk factors; therefore, we must consider the cumulative and intergenerational impacts when these major human rights violations are not addressed. Mitigating the impacts of violence involves evidence-based approaches encouraging healing and resilience-building among survivors as well as promoting justice system reform and addressing barriers to mental healthcare. We need to advance solutions on what works to prevent violence and prioritize existing efforts to engage with young men and adults to shift violent versions of masculinity. No single factor causes violence, and it is imperative to adopt multidisciplinary, multifaceted and systems-wide approaches to prevent violence and support survivors.

## Results

### Overview

**Extended Data Fig. 1:**
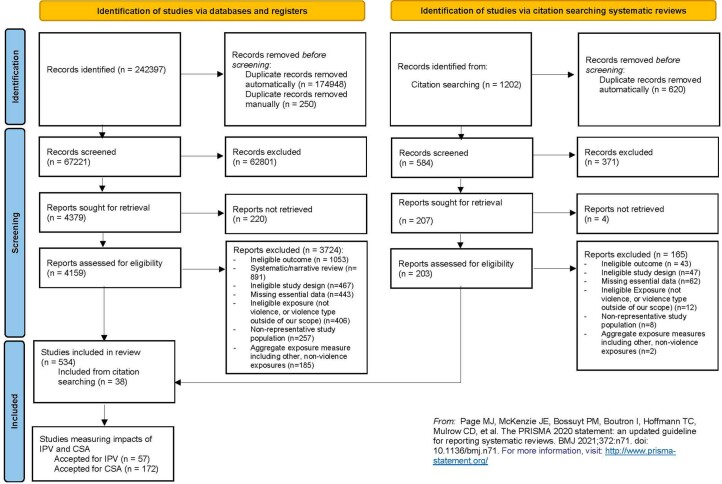
Flow Diagram of systematic review on health effects of intimate partner violence against women and childhood sexual abuse. The PRISMA flow diagram shows the data seeking approach undertaken as a part of our review, which sought to identify all literature reporting on the health effects of gender-based violence, violence against women, and violence against children and young people. Studies specifically measuring the health impacts of intimate partner violence against women (IPV) and childhood sexual abuse (CSA) were used for this analysis.

**Extended Data Fig. 2:**
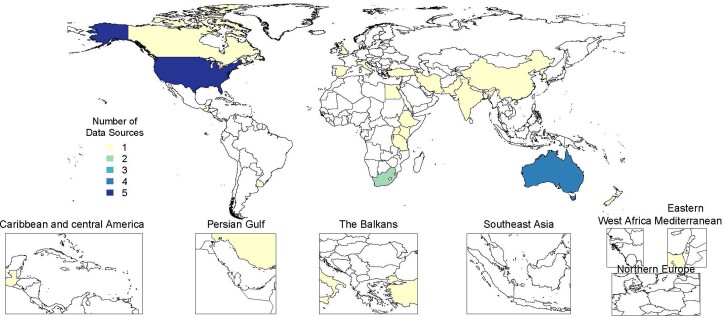
Data map of studies included in risk-outcome analyses for intimate partner violence against women and five health outcomes identified through the systematic review of the literature. Map shows number of studies conducted in a location.

**Extended Data Fig. 3:**
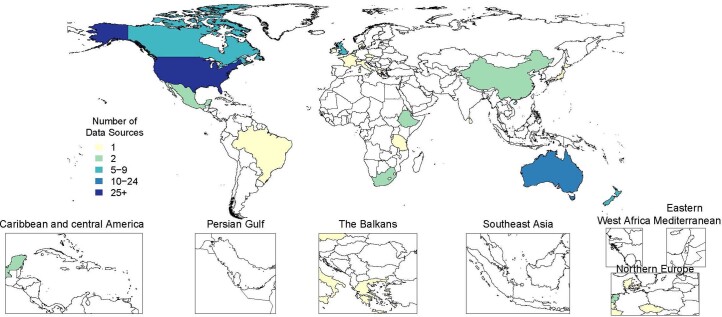
Data map of studies included in risk-outcome analyses for childhood sexual abuse and fifteen health outcomes identified through the systematic review of the literature. Map shows number of studies conducted in each location.

After conducting a systematic review on seven databases and de-duplicating records, we considered 67,221 records published between 1 January 1970 and 31 January 2023 (Extended Data Fig. 1 shows the Preferred Reporting Items for Systematic reviews and Meta-Analyses (PRISMA) flow diagram). In total, 4,379 articles met inclusion criteria during title and abstract screening and 534 of these were accepted for extraction after full-text screens. In this paper, we assessed and analyzed studies reporting specifically on intimate partner violence (*n* = 57) and childhood sexual abuse (*n* = 172). Maps displaying the count of studies identified by geographical location are displayed in Extended Data Fig. 2 (intimate partner violence) and Extended Data Fig. 3 (childhood sexual abuse). To undertake an analysis using the BPRF methodology, the health outcome studied must correspond to a GBD cause definition and we must have identified a minimum of three studies reporting on the relationship. For intimate partner violence, our models are specific to women and represent women populations only. For childhood sexual abuse, our estimates reflect both men and women, drawing upon data from studies using combined, women-only and men-only samples.

### Intimate partner violence

**Fig. 1:**
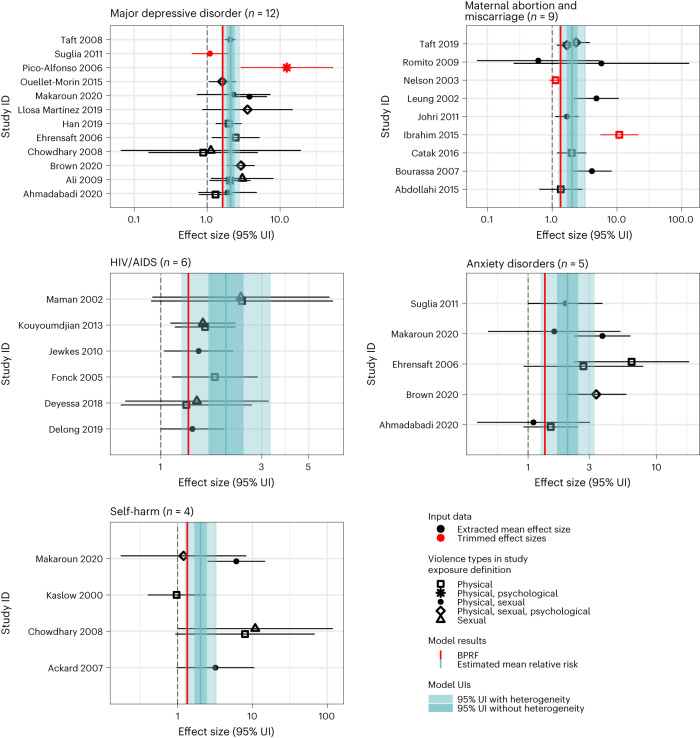
These forest plots present estimated mean relative risks, their 95% uncertainty intervals and the data points underlying the estimates for each of the five outcomes studied in association with intimate partner violence exposure (major depressive disorder, maternal abortion and miscarriage, HIV/AIDS, anxiety disorders and self-harm). The shape of the point indicates the type of intimate partner violence (physical, sexual and aggregate, including psychological) and the color indicates whether the point was detected and trimmed as an outlier. The light blue interval corresponds to the 95% UI incorporating between-study heterogeneity; the dark blue interval corresponds to the 95% UI without between-study heterogeneity. The black vertical dashed line reflects the null RR value (one) and the red vertical line is the burden of proof function at the fifth quantile for these harmful risk–outcome associations. The black data points and horizontal lines each correspond to an effect size and 95% UI from the included study identified on the *y* axis. We included multiple observations from a single study when effects were reported by form of violence, recall period and age group. Supplementary Table 15 contains more details on the included observations from each study.

**Extended Data Fig. 4:**
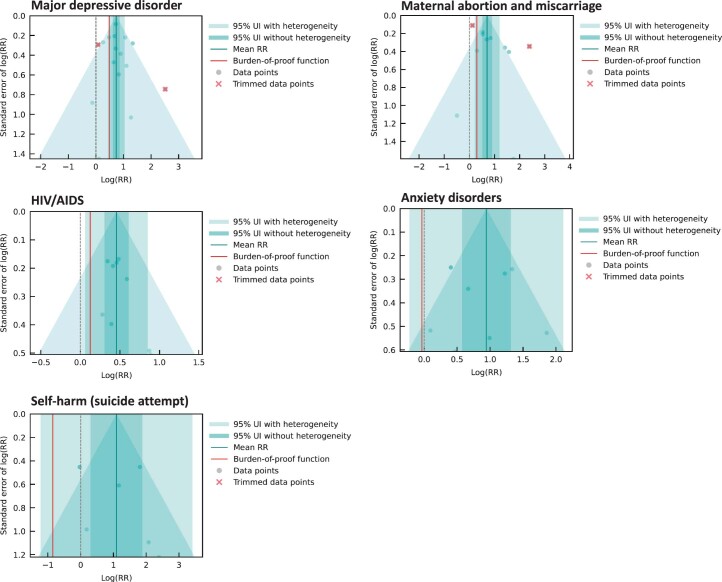
Funnel plots for meta-analysis of the association between intimate partner violence against women and five health outcomes identified through the systematic review of the literature. These modified funnel plots show the residuals of the reported mean relative risk (RR) relative to 0, the null value, on the x-axis and the residuals of the standard error, as estimated from both the reported standard error and gamma, relative to 0 on the y-axis. The light blue vertical interval corresponds to the 95% uncertainty interval (UI) incorporating between-study heterogeneity; the dark blue vertical interval corresponds to the 95% UI without between-study heterogeneity; the dots are each included observation; the red Xs are outliered observations; the grey dotted line reflects the null log(RR) of 0 (equivalent to a null RR of 1); the blue vertical line is the mean log(RR) for intimate partner violence and the outcome of interest; the red vertical line is the burden of proof function at the 5^th^ quantile for these harmful risk-outcome associations.

A total of five health outcomes were examined: major depressive disorder, maternal abortion and miscarriage, HIV/AIDS, anxiety disorders and self-harm. Estimates of the risk–outcome relationships are provided in Table 2. Forest plots are shown in Fig. 1 and funnel plots are shown in Extended Data Fig. 4.

**Table 2:** Strength of the evidence for the relationship between intimate partner violence against women and five health outcomes analyzed and childhood sexual abuse and 15 health outcomes analyzed

Risk factor	Health outcome	RR (95% UI without γ)	RR (95% UI with γ)	BPRF	ROS	Star rating	Pub. bias	No. of studies	Selected bias covariates	Risk–outcome pair in GBD 2021
Intimate partner violence	Major depressive disorder	2.1 (1.86, 2.37)	2.1 (1.55, 2.83)	1.63	0.24		No	12	None	Y
Intimate partner violence	Maternal abortion and miscarriage	2.03 (1.68, 2.46)	2.03 (1.25, 3.31)	1.35	0.15		No	9	Current and/or recent exposure	N
Intimate partner violence	HIV/AIDS	1.58 (1.36, 1.84)	1.58 (1.06, 2.34)	1.13	0.06		No	6	None	Y
Intimate partner violence	Anxiety disorders	2.57 (1.78, 3.72)	2.57 (0.8, 8.25)	0.97	−0.02		No	5	Current and/or recent exposure	N
Intimate partner violence	Self-harm	2.99 (1.36, 6.57)	2.99 (0.29, 30.25)	0.43	−0.42		No	4	None	N
Childhood sexual abuse	Alcohol use disorders	1.8 (1.62, 2.01)	1.8 (1.39, 2.33)	1.45	0.19		No	10	Unadjusted for confounding by age, sex and additional covariates; non-geographically representative study sample	Y
Childhood sexual abuse	Self-harm	1.98 (1.73, 2.26)	1.98 (1.25, 3.12)	1.35	0.15		No	16	Case–control study design (risk of reverse causation)	N
Childhood sexual abuse	Major depressive disorder	1.66 (1.51, 1.82)	1.66 (1.13, 2.44)	1.20	0.09		No	26	None	Y
Childhood sexual abuse	Anxiety disorders	1.44 (1.3, 1.6)	1.44 (1.13, 1.85)	1.17	0.08		No	12	Component outcome definition (for example, PTSD); exposure measured as experience before an age <15 (for example, 11–14)	N
Childhood sexual abuse	Asthma	1.25 (1.15, 1.35)	1.25 (1.06, 1.47)	1.09	0.04		No	4	None	N
Childhood sexual abuse	Type 2 diabetes mellitus	1.11 (1.04, 1.19)	1.11 (0.96, 1.28)	0.98	−0.01		No	7	None	N
Childhood sexual abuse	HIV/AIDS	1.34 (1.12, 1.61)	1.34 (0.87, 2.07)	0.93	−0.04		No	7	None	N
Childhood sexual abuse	Sexually transmitted infections excluding HIV	1.28 (1.04, 1.57)	1.28 (0.79, 2.08)	0.85	−0.08		No	4	None	N
Childhood sexual abuse	Maternal abortion and miscarriage	1.35 (1.11, 1.66)	1.35 (0.75, 2.44)	0.83	−0.09		No	6	None	N
Childhood sexual abuse	Drug use disorders	1.95 (1.57, 2.43)	1.95 (0.71, 5.38)	0.83	−0.09		No	16	None	N
Childhood sexual abuse	Conduct disorder	3.42 (1.64, 7.14)	3.42 (0.45, 25.7)	0.63	−0.23		No	3	Sample represents subpopulation; Unadjusted for confounding by age, sex and additional covariates; outcome is a specific drug use disorder	N
Childhood sexual abuse	Bulimia nervosa	2.95 (1.45, 5.97)	2.95 (0.37, 23.6)	0.51	−0.33		No	5	None	N
Childhood sexual abuse	Schizophrenia	3.7 (1.61, 8.53)	3.7 (0.26, 53.3)	0.40	−0.46		No	5	Confounding uncontrolled; Unadjusted for confounding by age, sex and additional covariates	N
Childhood sexual abuse	Ischemic heart disease	1.32 (0.86, 2.04)	1.32 (0.39, 4.47)	N/A	N/A		No	3	None	N
Childhood sexual abuse	Anorexia nervosa	2.07 (0.95, 4.51)	2.07 (0.22, 19.76)	N/A	N/A		No	4	None	N

The reported RR and its 95% uncertainty interval (UI) reflect the risk an individual who has been exposed to intimate partner violence or childhood sexual abuse has of developing the outcome of interest relative to that of someone who has not been exposed to these risk factors. Gamma (γ) is the estimated between-study heterogeneity. We report the 95% UI when not incorporating between-study heterogeneity (γ), ‘95%UI without γ’, and when accounting for between-study heterogeneity, ‘95% UI with γ.’ The BPRF is calculated for risk–outcome pairs that were found to have significant relationships at a 0.05 level of significance when not incorporating between-study heterogeneity (the lower bound of the 95% UI without γ does not cross the null RR value of one). The BPRF corresponds to the fifth quantile estimate of RR accounting for between-study heterogeneity closest to the null for each risk–outcome pair and it reflects the most conservative estimate of excess risk associated with intimate partner violence or childhood sexual abuse that is consistent with the available data. As we define intimate partner violence and childhood sexual abuse exposure as dichotomous risk factors (an individual either has been exposed or has not), the ROS is calculated as the signed value of log(BPRF) divided by two. Negative ROSs indicate that the evidence of the association is very weak and inconsistent. For ease of interpretation, we have transformed the ROS and BPRF into a star rating (0–5) with a higher rating representing a larger effect with stronger evidence. The potential existence of publication bias, which, if present, would affect the validity of the results, was tested using Egger’s regression. Included studies represent all available relevant data identified through our systematic reviews from January 1970 through January 2023. The selected bias covariates were chosen for inclusion in the model using an algorithm that systematically detects bias covariates that correspond to significant sources of bias in the observations included. If selected, the observations were adjusted to better reflect the gold standard values of the covariate. The Supplementary Information provides more information about the candidate bias covariates that were selected for in each model.

Among the five outcomes assessed for a relationship with intimate partner violence, two associations were found to have a three-star rating, suggesting moderate evidence of the risk–outcome relationship: major depressive disorder (0.24 risk–outcome score (ROS)) and maternal abortion and miscarriage (0.15 ROS). For major depressive disorder, the strongest relationship, we extracted 16 observations from 12 studies^22–33^ (nine cohorts and three case–control) across nine locations (Supplementary Table 1). Based on our conservative BPRF analysis, we estimated at least a 63% increase in risk of major depressive disorder (1.63 BPRF). No bias covariates were detected as significant or included in our main model. Estimated ROSs were similar in sensitivity analyses in which we subset data to cohort studies (three case–controls^25,28,31^ excluded) and excluded studies with exposure definitions including psychological intimate partner violence in addition to physical and/or sexual intimate partner violence^25,27,30,31^. Without applying 10% trimming of outliers (a likelihood-based statistical approach that limits the influence of outliers and identifies the 90% most self-coherent observations), estimated between-study heterogeneity increased and the strength of the association was reduced (Supplementary Table 2).

The outcome with the second highest number of identified and included studies was maternal abortion and miscarriage, for which we extracted 11 observations from nine studies^34–41^ (three cohorts and six case–controls) across nine locations (Supplementary Table 1). The estimated BPRF was 1.35 (at least a 35% increase in risk). Our bias covariate that flagged studies using exposure definitions measuring recent/current intimate partner violence (rather than lifetime intimate partner violence) was detected as significant and adjusted for within our final model. Main results were sensitive to analyses run without trimming outliers and excluding studies reporting low case counts (<10) in exposed and/or unexposed groups^39^ (Supplementary Table 3).

A two-star rating suggests that exposure to intimate partner violence increases the risk of a given outcome by 0–15% and can be interpreted as weak evidence of an association. HIV/AIDS was found to have a two-star rating of the association with intimate partner violence based on our conservative interpretation of the evidence (at least a 13% increase in risk, 0.06 ROS, 1.13 BPRF). We extracted nine observations from six studies^42–47^ (three cohorts and three other designs) across five locations in sub-Saharan Africa (Supplementary Table 1). No bias covariates were selected as significant for inclusion in the final model. Results were consistent with sensitivity analyses in which we subset the input data to prospective cohort studies only (three other study designs excluded^44,46,47^) and did not trim outliers (Supplementary Table 4).

A one-star rating suggests that exposure to intimate partner violence is weakly associated with the outcome under study and that introduction of additional evidence in the future may lead to changes in our assessment of this relationship. Our BPRF analysis yielded a one-star rating for two of the five studied outcomes: anxiety disorders (−0.02 ROS) and self-harm (−0.42 ROS). For anxiety disorders, we extracted eight observations from five cohort studies^23,24,26,30,32^ across three locations (Supplementary Table 1). Our bias covariate that flagged studies using exposure definitions measuring recent/current intimate partner violence (rather than lifetime intimate partner violence) was detected as significant and adjusted for within our final model. The results were consistent across several sensitivity analyses: the one-star rating persisted when removing trimming and when excluding a study that included psychological violence in its exposure definition^30^ (Supplementary Table 5).

The smallest number of studies^22,26,48,49^ (three cohorts and one case–control across two locations) was reported for an association with self-harm (operationalized across all included studies as suicide attempt) (Supplementary Table 1). No bias covariates were detected as significant, and thus none were included in the final model. When trimming a single outlying study, the overall estimated between-study heterogeneity was much lower, resulting in a three-star association (0.17 ROS). When excluding the one study^26^ that used an aggregate outcome definition, including suicidal ideation, the ROS decreased (−0.70 ROS; Supplementary Table 6). Across each of the health outcomes analyzed in association with intimate partner violence, we did not detect publication bias within our model results, as determined using Egger’s regression test^50^.

**Extended Data Fig. 5:**
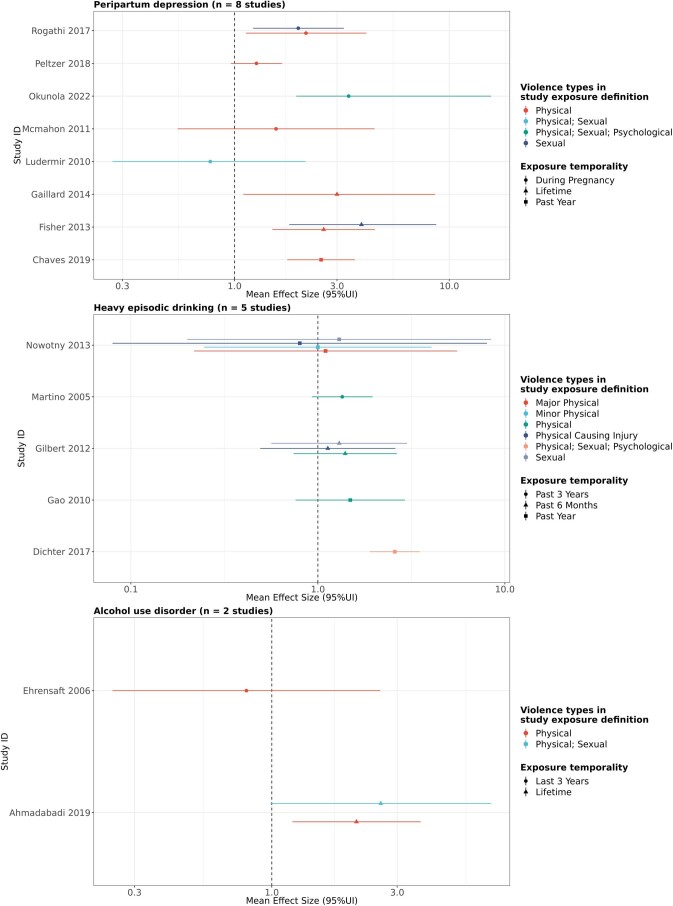
Forest plots showing extracted studies describing the association between intimate partner violence against women and the risk for peripartum depression, heavy episodic drinking, and alcohol use disorder. These forest plots present extracted mean relative risks (colored points) for each of the three outcomes for which we found studies in association with intimate partner violence exposure (peripartum depression, heavy episodic drinking, alcohol use disorder). The color of the point indicates type of intimate partner violence (that is, physical, sexual, aggregate including psychological) and the shape indicates the exposure temporality used in the author’s case definition of intimate partner violence. The horizontal lines indicate the 95% uncertainty interval from the included study. We included multiple observations from a single study when effects were reported by form of IPV, recall period, and age group. Okunala 2022 effect size has been extracted accurately as reported in the publication but should be considered with caution as lower and upper uncertainty interval bounds are not consistent with the mean estimate.

Additional outcomes that were identified in our review but not able to be analyzed using the BPRF methodology for either not meeting minimum data availability criteria (alcohol use disorder^32,51^, maternal hypertensive disorders^52,53^, gestational diabetes^52,53^ and maternal hemorrhage^53,54^ and sexually transmitted infections, excluding HIV^22,55^) or not mapping to an existing GBD cause (heavy episodic drinking^56–60^ and peripartum depression^61–68^) are presented in Supplementary Information 1.3. Extracted studies describing the risk of peripartum depression, heavy episodic drinking and alcohol use disorder are additionally visually summarized in Extended Data Fig. 5.

### Childhood sexual abuse

**Fig. 2:**
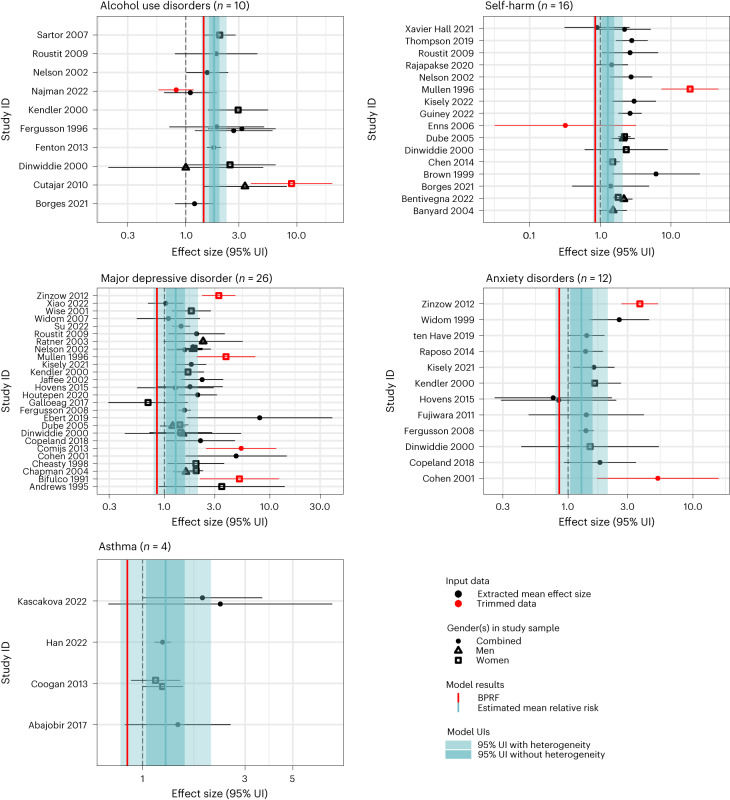
These forest plots present estimated mean relative risks, their 95% uncertainty intervals and the data points underlying the estimates for five outcomes studied in association with childhood sexual abuse and found to have a two- or three-star rating of the risk–outcome relationship (alcohol use disorders, self-harm, major depressive disorder, anxiety disorders and asthma). The shape of the point indicates gender of the sample, and the color indicates whether the point was detected and trimmed as an outlier. The light blue interval corresponds to the 95% UI incorporating between-study heterogeneity; the dark blue interval corresponds to the 95% UI without between-study heterogeneity. The black vertical dashed line reflects the null RR value (one) and the red vertical line is the burden of proof function at the fifth quantile for these harmful risk–outcome associations. The black data points and horizontal lines each correspond to an effect size and 95% UI from the included study identified on the *y* axis. We included multiple observations from a single study when effects were reported by severity/frequency of exposure and/or separately by gender or other subgroups. Supplementary Table 16 provides more details on the included observations from each study.

**Fig. 3:**
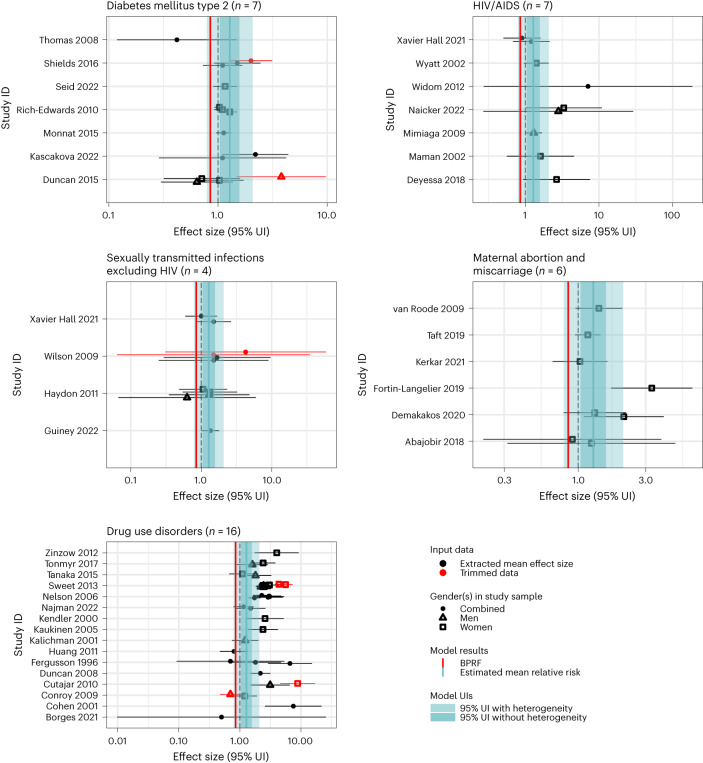
These forest plots present estimated mean relative risks, their 95% uncertainty intervals and the data points underlying the estimates for five outcomes studied in association with childhood sexual abuse and found to have a one-star rating of the risk–outcome relationship (diabetes mellitus, HIV/AIDS, maternal abortion and miscarriage, sexually transmitted infections excluding HIV and drug use disorders). The shape of the point indicates the sample gender and the color indicates whether the point was detected and trimmed as an outlier. The light blue interval corresponds to the 95% UI incorporating between-study heterogeneity; the dark blue interval corresponds to the 95% UI without between-study heterogeneity. The black vertical dotted line reflects the null RR value (one) and the red vertical line is the burden of proof function at the fifth quantile for these harmful risk–outcome associations. The black data points and horizontal lines each correspond to an effect size and 95% UI from the included study identified on the *y* axis. We included multiple observations from a single study when effects were reported by severity/frequency of exposure and/or separately by gender or other subgroups. Supplementary Table 16 provides more details on included observations from each study.

**Fig. 4:**
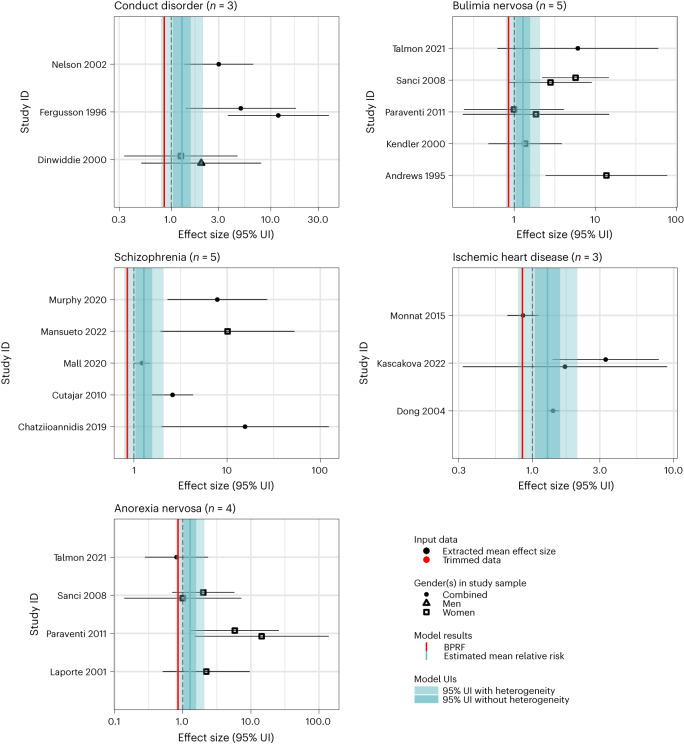
These forest plots present estimated mean relative risks, their 95% uncertainty intervals and the data points underlying the estimates for five outcomes studied in association with childhood sexual abuse and found to have a one- and zero-star rating of the risk–outcome relationship (conduct disorder, bulimia nervosa, schizophrenia, ischemic heart disease and anorexia nervosa). The shape of the point indicates the sample gender and the color indicates whether the point was detected and trimmed as an outlier. The light blue interval corresponds to the 95% UI incorporating between-study heterogeneity; the dark blue interval corresponds to the 95% UI without between-study heterogeneity. The black vertical dashed line reflects the null RR value (one) and the red vertical line is the burden of proof function at the fifth quantile for these harmful risk–outcome associations. The black data points and horizontal lines each correspond to an effect size and 95% UI from the included study identified on the *y* axis. We included multiple observations from a single study when effects were reported by severity/frequency of exposure and/or separately by gender or other subgroups. Supplementary Table 16 provides more details on the included observations from each study.

**Extended Data Fig. 6:**
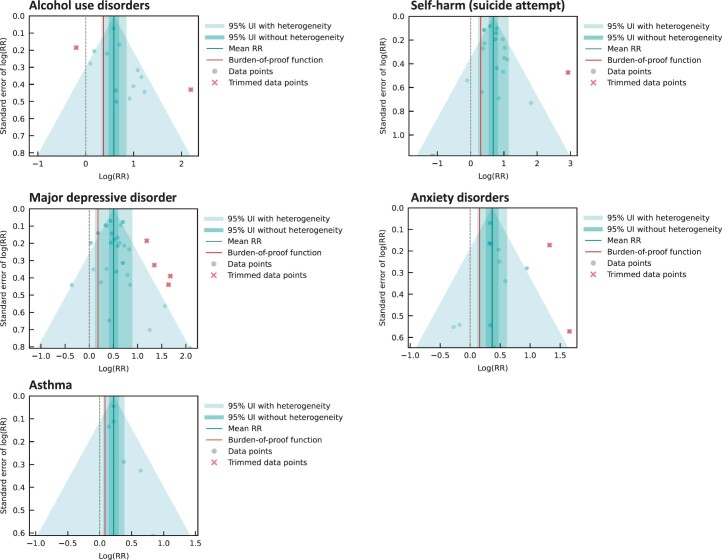
Funnel plots for meta-analysis of the association between childhood sexual abuse and five outcomes with an estimate three- or two-star association identified through the systematic review of the literature. These modified funnel plots show the residuals of the reported mean relative risk (RR) relative to 0, the null value, on the x-axis and the residuals of the standard error, as estimated from both the reported standard error and gamma, relative to 0 on the y-axis. The light blue vertical interval corresponds to the 95% uncertainty interval (UI) incorporating between-study heterogeneity; the dark blue vertical interval corresponds to the 95% UI without between-study heterogeneity; the dots are each included observation; the red Xs are outliered observations; the grey dotted line reflects the null log(RR) of 0 (equivalent to a null RR of 1); the blue vertical line is the mean log(RR) for childhood sexual abuse and the outcome of interest; the red vertical line is the burden of proof function at the 5^th^ quantile for these harmful risk-outcome associations.

**Extended Data Fig. 7:**
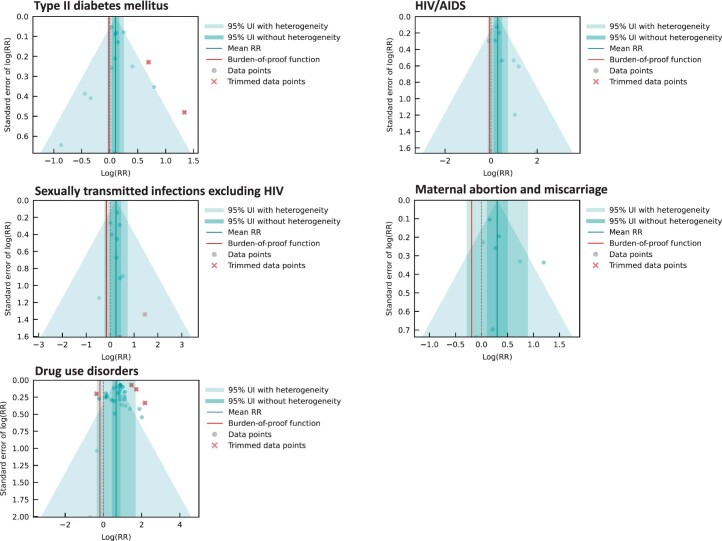
Funnel plots for meta-analysis of the association between childhood sexual abuse and five outcomes with an estimated one-star association identified through the systematic review of the literature. These modified funnel plots show the residuals of the reported mean relative risk (RR) relative to 0, the null value, on the x-axis and the residuals of the standard error, as estimated from both the reported standard error and gamma, relative to 0 on the y-axis. The light blue vertical interval corresponds to the 95% uncertainty interval (UI) incorporating between-study heterogeneity; the dark blue vertical interval corresponds to the 95% UI without between-study heterogeneity; the dots are each included observation; the red Xs are outliered observations; the grey dotted line reflects the null log(RR) of 0 (equivalent to a null RR of 1); the blue vertical line is the mean log(RR) for childhood sexual abuse and the outcome of interest; the red vertical line is the burden of proof function at the 5^th^ quantile for these harmful risk-outcome associations.

**Extended Data Fig. 8:**
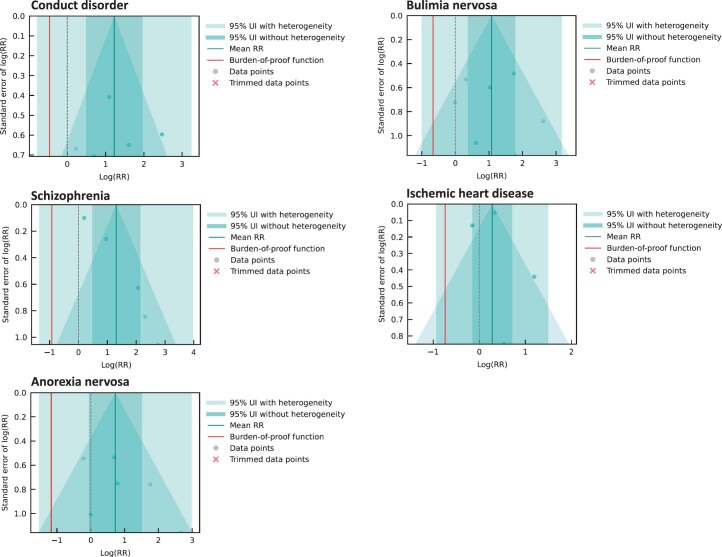
Funnel plot for meta-analysis of the association between childhood sexual abuse and five outcomes with an estimated one- or zero-star association identified through the systematic review of the literature. These modified funnel plots show the residuals of the reported mean relative risk (RR) relative to 0, the null value, on the x-axis and the residuals of the standard error, as estimated from both the reported standard error and gamma, relative to 0 on the y-axis. The light blue vertical interval corresponds to the 95% uncertainty interval (UI) incorporating between-study heterogeneity; the dark blue vertical interval corresponds to the 95% UI without between-study heterogeneity; the dots are each included observation; the red Xs are outliered observations; the grey dotted line reflects the null log(RR) of 0 (equivalent to a null RR of 1); the blue vertical line is the mean log(RR) for childhood sexual abuse and the outcome of interest; the red vertical line is the burden of proof function at the 5^th^ quantile for these harmful risk-outcome associations.

A total of 15 health outcomes met our minimum data availability criteria to investigate their associations with childhood sexual abuse. These were alcohol use disorders, self-harm, major depressive disorder, anxiety disorders, asthma, type 2 diabetes mellitus, HIV/AIDS, sexually transmitted infections, maternal abortion and miscarriage, drug use disorders, conduct disorder, bulimia nervosa, schizophrenia, anorexia nervosa and ischemic heart disease. Relevant estimates of the risk–outcome relationships associated with childhood sexual abuse are provided in Table 2. Forest plots are shown in Figs. 2–4 and funnel plots are displayed in Extended Data Figs. 6–8.

Among the outcomes assessed, we identified two with associations that yielded a three-star rating: alcohol use disorders (0.19 ROS) and self-harm (0.15 ROS). For alcohol use disorders, we extracted 15 observations from ten studies^69–78^ (nine cohorts and one case–control) across five locations (Supplementary Table 8). Alcohol use disorder was found to have a three-star rating of the association with childhood sexual abuse based on our conservative interpretation of the evidence (at least a 45% increase in risk, BPRF = 1.45). Two study-level bias covariates were found to be significant and adjusted for within the final model: geographic representativeness of the study and level of adjustment for confounding including age and sex. Results were sensitive to trimming in the model; without trimming, the estimated between-study heterogeneity increased substantially. We additionally undertook an analysis restricting our input dataset to only those studies that measured alcohol dependence (excluding studies using the accepted alternate definition of abuse and/or dependence^69,72,75,77^). When applying this restriction, results were consistent with our main analysis (0.20 ROS; Supplementary Table 9).

To estimate the association with self-harm (operationalized across all included studies as suicide attempt), we extracted 20 observations from 16 studies^71,74,77–90^ (14 cohorts and two case–controls) across nine locations (Supplementary Table 8). Our conservative interpretation of the evidence suggests at least 35% increase in self-harm risk given exposure to childhood sexual abuse with a BPRF of 1.35. Risk of reverse causation was found to be a significant bias covariate; thus, we conducted a sensitivity analysis restricting our input dataset to cohort studies only. Model results using cohort studies only^77,78,80,81,84–87,89,90^ were consistent with our main result (0.24 ROS; Supplementary Table 10).

We found a two-star rating for the association with three outcomes: major depressive disorder (0.09 ROS); anxiety disorders (0.08 ROS); and asthma (0.04 ROS). Childhood sexual abuse and major depressive disorder was the most data-rich risk–outcome pair across all health outcomes selected for analysis. We extracted 32 observations from 26 studies^71,73,74,78,83,87,91–110^ (22 cohorts and four case–controls) across ten locations (Supplementary Table 8). Based on our conservative interpretation, we estimate at least a 20% increase in risk of major depressive disorder, with a BPRF of 1.20. No bias covariates were found to be significant, and our main results were robust across sensitivity analyses: the estimated ROS decreased when removing 10% trimming (0.002 ROS) and increased when restricting the input dataset to cohort studies only^71,73,78,83,91–93,95,98–107,110^ (0.10 ROS) (Supplementary Table 11).

The association between childhood sexual abuse and anxiety disorders was also relatively data-rich; we extracted 13 observations from 12 studies^73,74,95,97,99,102,104,110–114^ (11 cohorts and one case–control) across five locations (Supplementary Table 8). Our conservative interpretation of the evidence suggests at least 17% increase in anxiety disorder risk given exposure to childhood sexual abuse, with a BPRF of 1.17. Covariates marking whether a study measured specific anxiety disorders, for example, social phobia and measuring childhood sexual abuse as sexual violence that occurred at an age younger than 15 were found to be significant and adjusted for in the final model. The ROSs were stable across sensitivity analyses that excluded a case–control study^111^ (0.08 ROS), excluded a study using administrative records to ascertain exposure^114^ (0.07 ROS) and excluded studies measuring specific anxiety disorders (for example, post-traumatic stress disorder) only^73,74,110,114^ (0.06 ROS; Supplementary Table 12).

While we identified only four studies^115–118^ reporting on the association with asthma, the studies reporting on this outcome consistently demonstrated a harmful effect and our model results found weak evidence of an association (0.04 ROS). Our conservative interpretation of the evidence suggests at least 9% increase in asthma risk given exposure to childhood sexual abuse, with a BPRF of 1.09.

We found weak evidence of association (one-star rating) for eight additional health outcomes: type 2 diabetes mellitus (number of accepted studies (*n*) = 7 (refs. ^118–124^); −0.01 ROS), HIV/AIDS (*n* = 7 (refs. ^44,47,90,125–128^); −0.04 ROS), sexually transmitted infections, excluding HIV (*n* = 4 (refs. ^85,90,129,130^); −0.08 ROS), maternal abortion and miscarriage (*n* = 6 (refs. ^37,131–135^); −0.09 ROS), drug use disorders (*n* = 16 (refs. ^69,72,73,75,77,95,110,136–144^); −0.09 ROS), conduct disorder (*n* = 3 (refs. ^71,74,75^); −0.23 ROS), bulimia nervosa (*n* = 5 (refs. ^73,91,145–147^); −0.33 ROS) and schizophrenia (*n* = 5 (refs. ^72,148–151^); −0.46 ROS).

Further describing drug use disorders as an example, we extracted 16 studies^69,72,73,75,77,95,110,136–144^ that reported on the association with childhood sexual abuse exposure (14 cohorts and two case–controls across five locations; Supplementary Table 8). We accepted author definitions that measured drug use disorders, drug abuse and illicit drug use. While most studies investigated drug use disorders and illicit drug use in aggregate (*n* = 14), we also accepted studies that investigated relationships with specific substances if this was the only outcome available (*n* = 2)^136,137^. We incorporated a bias covariate for data points measuring specific substances only and for studies measuring use rather than use disorders diagnosed via DSM criteria; measuring a specific use disorder was found to be a significant source of bias in our main model. Two bias covariates related to control for confounding and sample representativeness were also found to be significant and adjusted for within the final model (Supplementary Table 13). Results were relatively stable across sensitivity analyses in which we removed studies measuring specific drug use disorders only^136,137^ (opioid dependence and cannabis dependence; −0.12 ROS) and those measuring use rather than disorders^138–141,143,144^ (−0.13 ROS) (Supplementary Table 13).

Among the estimated one-star risk–outcome pairs, four health outcomes (type 2 diabetes mellitus^118–124^, HIV/AIDS^44,47,90,125–128^ and maternal abortion and miscarriage^37,131–135^, and drug use disorders^69,72,73,75,77,95,110,136–144^) had greater than five input studies identified (Table 2). Detailed analysis approaches and results for the additional outcomes for which the association with childhood sexual abuse received a one-star ranking are reported in Table 2 and Supplementary Information 1.6.

Last, for two additional health outcomes, ischemic heart disease and anorexia nervosa, the available evidence (ischemic heart disease, *n* = 3 (refs. ^118,123,152^); anorexia nervosa, *n* = 4 (refs. ^145–147,153^)) does not support a significant association with childhood sexual abuse exposure. For each of these outcomes, the estimate of conventional relative risk (RR) was not statistically significant (uncertainty estimated without accounting for between-study heterogeneity crossed the null RR of 1) and these outcomes were rated as zero stars (ischemic heart disease, RR = 1.32 (0.86–2.04); anorexia nervosa, RR = 2.07 (0.95–4.51)). These outcomes therefore do not meet the criteria to be considered for inclusion in the GBD. Across each of the health outcomes analyzed in association with childhood sexual abuse, we did not detect publication bias within our model results, as determined via Egger’s regression test^50^.

**Extended Data Fig. 9:**
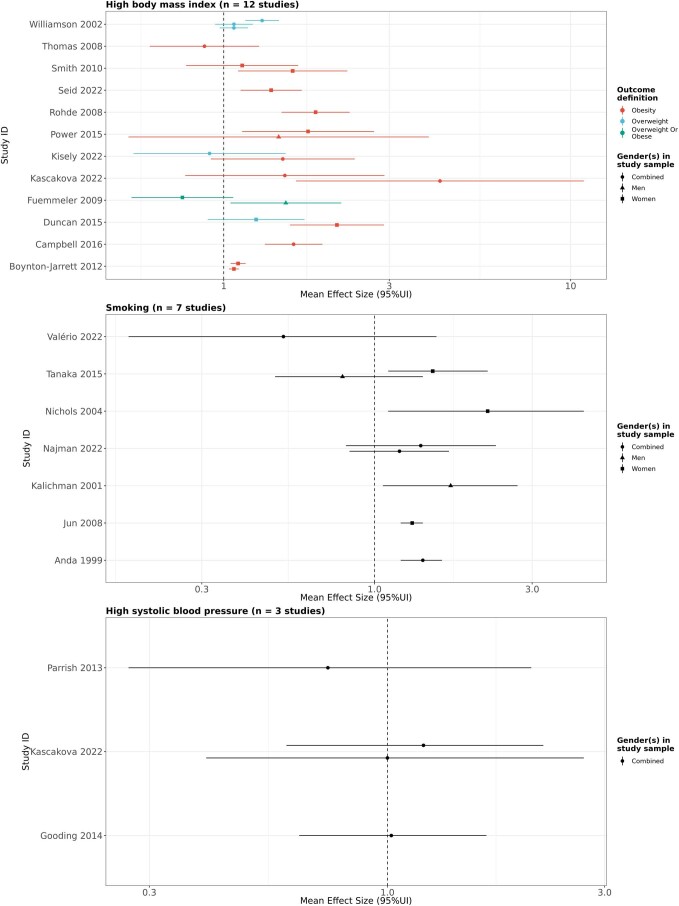
Forest plots showing extracted studies describing the association between childhood sexual abuse and the risk for high body mass index, smoking, and high systolic blood pressure. These forest plots present extracted mean relative risks (colored points) for each of the three outcomes for which we found studies in association with childhood sexual abuse exposure (high body mass index, smoking, high systolic blood pressure). In the first panel (high body mass index) the color of the point indicates whether the study describes obesity or overweight as an outcome. In all panels, the shape of the point indicates gender of the study sample. The horizontal lines indicate the 95% uncertainty interval from the included study. We included multiple observations from a single study when effects were reported by severity/frequency of exposure and/or separately by gender or other subgroups.

In addition to the GBD causes described above, some health-related risk factors were also reported in association with exposure to childhood sexual abuse in the literature, including high body mass index^118,119,121,124,154–161^, smoking^69,139,143,162–165^ and high systolic blood pressure^118,166–168^. These outcomes are presented in Supplementary Information 1.7 and extracted studies are visually summarized in Extended Data Fig. 9.

## Discussion

Our comprehensive systematic review yielded data to examine associations between intimate partner violence against women and five health outcomes, and between childhood sexual abuse and 15 health outcomes. This expands the number of health outcomes previously investigated for either risk factor and risk–outcome pairs evaluated for inclusion in the GBD.

Despite the wide-ranging nature of our systematic review, the outcomes presented here likely remain an under enumeration of the total number of health outcomes associated with intimate partner violence and childhood sexual abuse. Our literature search identified numerous health conditions (such as sexually transmitted infections, substance use disorders and high systolic blood pressure) that had fewer than three studies and therefore could not be analyzed using the BPRF methodology. Also, certain outcomes associated with either risk factor may not be captured through longitudinal study designs that were the focus of this review (for example, the immediate physical injuries such as traumatic brain injuries). We did not identify consequences such as these within our review; however, studies drawing on cross-sectional surveys that ask survivors about injuries experienced as a consequence of violence and high-quality health system databases offer a promising avenue to quantify these immediate health impacts. The disease burden attributable to intimate partner violence and childhood sexual abuse may not be fully captured without accounting for these additional outcomes and they represent an important area of continued research.

Moreover, we observed a high level of variability in control for confounding and strategies for analyzing potential mediating factors across the studies that we identified examining the health effects of intimate partner violence and childhood sexual abuse. It is likely that the health impacts of intimate partner violence and childhood sexual abuse exposure are mediated through certain physical health risk factors such as high body mass index and smoking, as indicated by our descriptive review of studies and existing meta-analyses^5^. To further clarify the pathways between exposure to violence and long-term physical health outcomes, the use of high-quality, prospective cohorts and generation of consensus on appropriate consideration for confounding and/or mediation is needed. Additionally, the interplay between adversity and socioeconomic deprivation (a factor not well captured in the current literature) and its consequences for long-term health should be considered in future analyses^169^.

The sobering reality remains that violence against women and children continues to be a neglected area within global health. In addition to highlighting the scale of violence against women and children, our study also highlights the overall dearth of evidence on violence against women and children, especially when compared to other risk factors such as smoking and high blood pressure. The most studied outcome (major depressive disorder) in our analysis is supported by 12 studies for intimate partner violence and 26 for childhood sexual abuse. In contrast, the most studied outcomes for smoking and high blood pressure are each supported by over 75 studies^170,171^. A lack of research limits our ability to understand the true health impacts of violence; for example, out of all the mental health outcomes explored, only one (anorexia nervosa), did not have strong enough evidence to support an association with childhood sexual abuse. This result likely stems from the low number of studies identified and should be interpreted as an important call for additional research, especially when considering the evidence of an association found for bulimia nervosa, a disorder for which the mechanism of an association is plausibly similar. It is also of note that the studies identified in our review largely represent populations in high-income countries, with a relatively limited number of studies identified for either risk factor in low- and middle-income settings. To the extent that research funding and research output correspond to the level of attention given to a health problem, these patterns demonstrate that violence against women and children is grossly understudied and its significance is underappreciated. Until it is prioritized as an important component of health and well-being, the evidence base will remain weak and, in turn, it will continue to be a neglected global health issue.

While advancing the evidence base on the health impacts of intimate partner violence and childhood sexual abuse is critically important, there is also the need to recognize that those experiencing these forms of violence will continue presenting to health services globally. The health sector provides a window of opportunity to intervene on intimate partner violence as it is often the only place outside the home women are consulting with others, especially during pregnancy. As outlined by the WHO^172^, interventions to address violence against women need to be included as a strategic priority in national health policies, with accompanying budget allocations and evidence-based clinical protocols to guide healthcare providers in caring for survivors. Ample evidence suggests that women welcome being asked by providers about their experiences of violence, as long as it is conducted in a professional manner to avoid traumatization and with referral options in place^173^. For this to take place, screening needs to be integrated at all levels of the health sector, including through staff training, clear policies, coordination among departments planning integrated services and effective referrals within the health sector to external services^174^.

In line with our findings, there is an emerging body of research that has demonstrated the impacts of abuse and violence beyond associations with mental health to also include physical health conditions. For example, as noted by the American Heart Association, there is an urgent need to consider the role of childhood adversity on future cardiovascular disease risk among other health conditions^118^. Approaches to mitigate these effects include the adoption of holistic and family-orientated programs, which have shown great promise in the reduction of risk factors that mediate the pathways (reducing inflammation and encouraging smoking cessation) between childhood sexual abuse and subsequent disease^119–121^. In addition to addressing specific mediators, another hypothesis is to encourage the adoption of approaches that build resilience in survivors, although many such approaches require further rigorous evaluation^122^.

For both intimate partner violence and childhood sexual abuse, there remains the need to expand the existing evidence base on what works to prevent, as well as treat and support survivors^175^. Current approaches include the development of gender-transformative interventions that engage men to change violent versions of masculinity and power inequity in relationships^176^ and survivor-centered movements such as the Brave movement that bring together prevention, healing for victims and justice system reform supporting victims and survivors^177^. No single factor causes violence and it is imperative to adopt multidisciplinary, multifaceted and systems-wide approaches to support evidence-based interventions to raise awareness, change societal norms, provide social and economic empowerment to those at risk and promote healthy relationships and adversity-free childhoods^175^.

The study has some limitations. First, there was considerable between-study heterogeneity in definitions of intimate partner violence and childhood sexual abuse. For example, the GBD defines intimate partner violence as physical and/or sexual violence over the lifetime, yet we also included information from studies that report on psychological violence and recent experiences (for example, past year only) so that we could include all of the available evidence. To account for the differences in definition, we introduced study-level bias covariates. The bias covariate capturing timing of exposure was significant for certain outcomes; for example, patterns in extracted data suggest that recent violence exposure may have a greater effect on maternal abortion and miscarriage. Potential mechanisms for this pattern include the direct physical consequences of violence, associated abusive behaviors such as reproductive coercion^178^ and women’s decision-making process around terminating a pregnancy^179^. We did not observe significant differences when testing the impact of including studies that also measured psychological violence. Yet, psychological partner violence likely carries substantial and specific health burdens, particularly for mental health. Future work should investigate the impacts of each form of intimate partner violence in addition to the impact of experiencing its multiple forms simultaneously. Existing cross-sectional evidence suggests that combined forms of exposure to partner violence are not only more prevalent than singular experiences but also more damaging to health^180^.

A second limitation is our conceptualization of intimate partner violence and childhood sexual abuse as dichotomous risks, which collapses the specific effects of the timing, accumulation and frequency of experiences of violence into one category. Some studies extracted for this analysis attempted to investigate dose–response effects by defining exposure by the severity and frequency of acts experienced; however, the results of these studies are inconsistent. Additionally, most studies did not specifically investigate the impact of exposure timing on the studied outcomes. For example, commonly used childhood sexual abuse indicators (including the GBD definition) define exposure as an experience of sexual violence occurring before a specific age (for example, 18), and it is not possible to detect the potential impact of exposure timing using this type of indicator definition as it collapses many potential ages of exposure into a binary category. By drawing upon longitudinal and linked datasets, it may be possible in the future to estimate the health effects of experiencing more than one type of violence, violence at specific developmental periods and violence at multiple points in the life course.

The operationalizations of intimate partner violence and childhood sexual abuse in the GBD are specific to age, form of violence experienced and/or perpetrator identity. Many individuals’ experiences of violence may not neatly fit into these operationalizations (or fit simultaneously into multiple categories of abuse) and it is crucial to improve the evidence surrounding health impacts of all forms of exposure to violence against women and children, including but not limited to psychological violence, cyberviolence, stalking, reproductive coercion and more. Future work will begin to investigate the health impacts of experiences that extend beyond and/or cross over definitions of intimate partner violence against women and childhood sexual abuse. Evidence generated from this work will make possible both a re-examination of existing case definitions and assessments of new forms of violence exposure for inclusion as risk factors into GBD.

Third, in our analyses, we created bias covariates that accounted for study-level adjustments to control for age, sex and the number of additional confounders (regardless of the identity of the additional confounders). We observed a high degree of variability in which confounders were measured across the literature and it is possible that some covariates should have been included as mediating variables rather than used to control for confounding (for example, variables on the causal pathway between exposure and outcome, such as depressive symptoms in the relationship between childhood sexual abuse and substance use disorders). Due to the relatively low number of studies analyzed per risk–outcome pair, it was not feasible for us to disentangle how or if included confounders drove differences between studies. This is an area that needs further investigation, especially with regard to measuring confounding and exposure over time using a life course approach.

Finally, given the strong associations with mental health disorders found in our analyses, it is important to note that the GBD framework (which is used to estimate years of life lost and lived with disability attributed to both risk factors) does not reflect differential mortality gaps for mental health disorders, except anorexia and bulimia nervosa. Premature mortality due to mental disorders can occur through a variety of conditions, including self-harm as well as infectious and chronic diseases^181–183^; however, these deaths are assigned to the most proximate cause (for example, suicide) within the GBD. It may be possible in the future to utilize the comparative risk assessment framework to quantify mental disorders’ contribution to mortality and more completely capture health loss due to mental health conditions and their upstream risk factors, including intimate partner violence and childhood sexual abuse.

This systematic review highlights the wide-ranging health consequences associated with intimate partner violence and childhood sexual abuse, ultimately informing key steps across the WHO Public Health Approach to Violence by advancing estimates of the relative risks of selected health outcomes associated with each form of abuse^184^. Intimate partner violence and childhood sexual abuse, which represent only a subset of all forms of violence against women and children, not only affect individuals but whole societies and economies—as exposure to these risk factors increases demands on overstretched health systems and perpetuates poverty and gender inequality by constraining educational attainment and economic productivity of the survivors and their families^1,185^. Although the current research trajectory often creates distinctions between violence in adulthood and childhood, in light of their shared risk factors, co-occurrence and compounding consequences across the life course, there is a clear need to examine intimate partner violence and childhood sexual abuse in unison^15^ and in the context of the wider health and societal risks of violence against women and children. Individuals at risk often fit into several categories (for example, girls below age 18 years are subject to the challenges of both intimate partner violence and childhood sexual abuse). Policymakers, practitioners and researchers are increasingly shifting to working in both domains to encourage a unified approach to detecting and addressing violence throughout the life course^1^. Investing in evidence-based interventions to prevent violence against women and children, in all forms, and provide appropriate support to survivors will result in both short- and long-term gains for individuals, their families and societies overall.

## Methods

### Overview

This study used the BPRF methodology to estimate the risk of health outcomes in association with exposure to intimate partner violence and childhood sexual abuse and to assess the strength of evidence underlying these relationships. The BPRF approach was developed by Zheng and colleagues^21^ and employs a meta-analytic tool, MR–BRT (meta-regression–Bayesian, regularized, trimmed) to estimate relative risks and uncertainty estimates that incorporate between-study heterogeneity. This approach has previously been used to evaluate the health impacts of multiple risk factors, including smoking and red meat consumption. In this study, we apply the approach to intimate partner violence and childhood sexual abuse, modeled as dichotomous risk factors, via six main analytical steps: (1) systematically reviewing the literature and extracting data from identified studies; (2) estimating a pooled relative risk comparing the risk of health outcomes to individuals exposed to the selected risk factor relative to non-exposed individuals; (3) evaluating and adjusting for systematic sources of bias within input studies; (4) estimating between-study heterogeneity while accounting for within-study correlations and incorporating this estimate into uncertainty intervals; (5) detecting potential publication bias using Egger’s regression test; and (6) estimating the BPRF, defined as the fifth quantile estimate of the risk closest to the null estimate and corresponding ROS.

We applied the BPRF methodology to risk–outcome relationships for which we identified at least three studies in the scientific literature. We estimated relative risks and BPRF and ROS values for each risk–outcome pair using all identified studies in a single model and generated results that were not location- or age-specific. For intimate partner violence, our models are specific to women. For childhood sexual abuse, our estimates reflect both men and women, drawing upon all available data regardless of how or if the input study collected and reported data by sex or gender.

We followed the PRISMA guidelines^186^ through all stages of this study (Supplementary Information 3.1). This study complies with the Guidelines on Accurate and Transparent Health Estimates Reporting (GATHER) recommendations^187^ (Supplementary Information 3.2). The study was approved by the University of Washington Institutional Review Board (study no. 9060), and the systematic review approach was registered in PROSPERO (CRD42022299831). We have previously published our review protocol^188^.

### Systematic review

Our systematic review process took place within a larger project that aimed to identify and synthesize all available data on the health impacts of exposure to any form of violence against women, gender-based violence (GBV) and violence against children (VAC) and young people, which included intimate partner violence and childhood sexual abuse^188^. In turn, our research forms part of the efforts of the *Lancet* Commission on Gender-based Violence and Maltreatment of Young People and specifically the workstream focused on better measuring the epidemiological profile and the need for action based on the health consequences of interpersonal violence against women and children^1^.

We systematically searched seven databases (PubMed, Embase, CINAHL, PsycINFO, Global Index Medicus, Cochrane and Web of Science Core Collection) for all relevant studies published between 1 January 1970 and 30 September 2021. We started our searches in the year 1970, in keeping with the start of most literature databases, improved quality of scientific literature and the standard approach to systematic review searching within the GBD. As of 15 February 2023, searches were updated to incorporate articles published and/or added to databases between 30 September and 31 January 2023. Our systematic review of the health implications followed the PRISMA guidelines^186^ and was conducted in line with our previously published protocol (PROSPERO, CRD42022299831)^188^. Our search strings are reported in Supplementary Information 4.1 and have been previously published^188^.

In brief, our search strategy incorporated keyword and controlled vocabulary restrictions corresponding to (1) violence exposure; (2) study design and type; (3) measures of association and/or risk; and (4) publication year. Searches were not restricted to predetermined health outcomes in an effort to identify and extract all literature reporting on an association between GBV and/or VAC and health. During article screening and selection, we drew upon definitions of health outcomes from the cause, injury and risk factor case definitions used by the GBD study^10,189^. While we identified and extracted studies reporting on a variety of GBV and VAC case definitions, in the present study we report only results from studies that described exposure to intimate partner violence and/or childhood sexual abuse. We define intimate partner violence and childhood sexual abuse according to the GBD study case definitions: lifetime prevalence of physical and/or sexual violence by a current or former intimate partner since age 15 and lifetime prevalence of intercourse or other contact abuse (fondling or other sexual touching) when aged 15 years or younger in which the contact was unwanted or perpetrator was 5+ years older than the victim, for intimate partner violence and childhood sexual abuse, respectively.

We utilized the systematic review software Covidence to manage our review process, including the automated de-duplication of search results across different databases. Our inclusion criteria were case–control, cohort or case–crossover studies conducted in participant groups likely to be generalizable and reporting a relative measure of association or number of cases and non-cases among exposed groups (defined as any individual who has experienced a form of intimate partner violence and/or childhood sexual abuse throughout the lifetime) versus non-exposed comparators. Our exclusion criteria included cross-sectional, ecological, case series or case studies; studies conducted in subgroups identified via a shared characteristic associated with the exposure and/or outcome under study; studies that reported only aggregate measure of exposure combining violence exposure with other, non-eligible exposures; and studies missing essential data (effect sizes and/or appropriate uncertainty information; Supplementary Information 4.2). Studies reporting cross-sectional designs were accepted only when exposure ascertainment was retrospective, ensuring that exposure preceded the current health outcomes being evaluated.

We used the above-described criteria to title and abstract screen 67,221 identified articles. Each review step (title/abstract screening, full-text screening and data extraction) began with consensus-building exercises across the review team. After training and consensus-building, the first two-thirds of titles/abstracts were reviewed by two independent reviewers, with conflicts resolved by project leaders. Upon confirmation of a low rate of total conflicts (<5% of screened), the remainder of titles/abstracts were single screened. Non-English articles were screened by reviewers with proficiency in the language. Studies that met inclusion criteria during title/abstract screening (*n* = 4,379) were full-text screened and excluded if found to meet any exclusion criteria. Two independent reviewers full-text screened 10% of articles, with conflicts resolved by project leads. Upon confirming a low conflict rate (<5%), the remaining 90% of articles were single screened. In total, we accepted and extracted 496 articles reporting on health impacts of any form of GBV and/or VAC.

We supported our primary search results by identifying and citation searching systematic reviews/meta-analyses for additional references (Supplementary Information 4.3). Briefly, we screened systematic reviews/meta-analyses according to our review criteria and categorized included reviews by risk–outcome pair. We selected the highest-quality systematic review (determined based on recency, journal impact factor, adherence to PRISMA and GATHER guidelines and quality of search strategy) per risk–outcome pair to citation search. Extracted citations (*n* = 1,202) were cross-referenced against studies screened in our review, and newly identified articles (*n* = 584) were screened according to our review criteria, resulting in an additional 38 articles accepted for extraction.

In summary, these data formed the larger pool of studies from which we drew the inputs for the present investigation, which assessed studies measuring intimate partner violence (*n* = 57) or childhood sexual abuse (*n* = 172) specifically. All articles were extracted using a modified Covidence v.2.0 extraction template (Supplementary Table 20). Variables collected during data extraction corresponded to study characteristics; population and sample characteristics; exposure and outcome assessment; and effect sizes and uncertainty estimates. Our extraction procedure included collecting standardized information on sources of potential bias within studies, discussed further in our evaluation of publication bias methods section.

### Data selection

Drawing upon all extracted studies, we identified health outcomes for which at least three studies were identified with a comparable exposure and outcome definition. Health outcomes were defined according to GBD reference and accepted alternate reference definitions (Supplementary Information 4.5), for which we incorporated covariates to detect whether their inclusion was a source of bias in final model results. For the purposes of the present study, we did not investigate associations between intimate partner violence during pregnancy and adverse birth outcomes. Low birthweight and short gestation are considered risk factors within the GBD and our analyses were restricted to GBD causes of disease and injury only. Relationships between risk factors (for example, intimate partner violence and low birthweight) are not currently incorporated within the BPRF methodology; however, these data were accepted in our broader review process and a separate study will investigate the health impacts of partner violence experienced during pregnancy and adverse birth outcomes. We also only accepted studies using highly specific diagnostic tools to diagnose specific mental disorders (rather than measuring general or overall psychological distress). For depressive and anxiety disorders specifically, we followed guidance from mental health research in the GBD and accepted studies measuring these outcomes via a list of acceptable diagnostic interviews and/or symptom scales (Supplementary Information 4.5.1). For other mental disorders, we followed GBD case definitions and accepted studies measuring outcomes by use of International Disease Classification and Diagnostic and Statistical Manual of Mental Disorder criteria. Evidence exists describing a bi-directional relationship between intimate partner violence and mental health outcomes^11,12^. We therefore only accepted study designs in which exposure preceded outcome.

In forming our input datasets for intimate partner violence models, we accepted author definitions of exposure matching the GBD case definition (physical and/or sexual intimate partner violence), those that measured physical intimate partner violence only and those that measured sexual intimate partner violence only. Due to data sparsity, we additionally accepted studies with author definitions that included psychological violence in addition to physical and/or sexual (defined exposure as any intimate partner violence involving physical, sexual and/or psychological abuse). Potential bias due to using an accepted alternate exposure definition was accounted for in our modeling process via two study-level bias covariates marking component exposure definitions and aggregate exposure definitions (Supplementary Table 23). We did not include author definitions measuring psychological intimate partner violence only, economic/financial intimate partner violence only or those reporting aggregate definitions incorporating economic intimate partner violence.

In forming our input datasets for childhood sexual abuse models, we accepted author definitions of exposure using any age threshold ≤18 years by any perpetrator. The GBD case definition considers the age for sexual abuse to be less than 15 years old; however, 18 is commonly accepted as the age of majority in many countries and is used in other global childhood sexual abuse indicators (for example, Sustainable Development Goal Indicator Target 16.2.3; ref. ^190^). Therefore, we sought to recognize that sexual violence experienced from ages 15–18 is considered childhood sexual abuse in many contexts. For definitions that used alternate ages of exposure, we incorporated a study-level bias covariate to test the impact of setting different upper bounds of the ages that constitute childhood. In addition, certain studies measured childhood sexual abuse perpetrated in the context of specific relationships (family member-perpetrated childhood sexual abuse); estimates from these studies were marked with a bias covariate indicating that a restricted perpetrator definition was used (Supplementary Table 24).

In the case that included studies for a given risk–outcome pair reported on the same underlying cohort or dataset, we compared the relative quality of each publication and selected only one publication to avoid undue influence of the specific cohort/sample in the model. Where possible, the least granular analyses were selected for use in modeling; however, for studies that only reported multiple effect sizes by non-overlapping subgroups (for example, reporting effects separately by age or sex, or gender strata), all effect sizes were included and were not adjusted as they reflected unique participants within the overall sample. If a study reported more than one effect size for multiple, non-mutually exclusive exposure/outcome definitions (for example, physical intimate partner violence and sexual intimate partner violence reported separately, with each exposure group formed without reference to the other type of exposure), we adjusted the standard errors of the observations by a factor matching the number of repeated measurements across the same sample to prevent overweighting these studies in model results (Supplementary Information 5.2). If studies reported effect sizes for repeated periods of exposure measurement (for example, measured intimate partner violence in the past year and over the lifetime), the effect size calculated using the exposure case definition closest to the GBD definition and/or with the best control for temporality between exposure and outcome was selected.

We did not observe consistent gender-specific effects of childhood sexual abuse across studies that reported effect sizes stratified by sex or gender; however, there is a potential compositional bias in childhood sexual abuse studies that represent women-only samples. Thus, for all childhood sexual abuse analyses, we included a bias covariate to indicate studies that reported on gender-specific samples. If this covariate was detected as significant within our selection algorithm, we undertook a sensitivity analysis constraining the input dataset to studies reporting effects across men and women.

For each risk–outcome pair meeting the three-study threshold, we used the MR–BRT tool to perform a meta-regression analysis to estimate the risk of the given outcome for those exposed to intimate partner violence and childhood sexual abuse relative to unexposed counterparts. Following the BPRF methodology, for risk–outcome pairs with sufficient data available (≥10 observations), we introduced likelihood-based trimming (10%) to detect and remove outliers that may otherwise over-influence the model.

### Statistical analysis

Analyses were carried out using R v.4.0.5 and Python v.3.8.

#### Testing and adjusting for biases across study designs and characteristics

Following the Grading of Recommendations, Assessment, Development and Evaluations (GRADE) approach^191^, the extracted risk of bias criteria for individual studies included (1) exposure measurement method (instrument or survey used) and data source (self-reported versus ascertained from administrative sources of information such as legal or healthcare databases); (2) outcome measurement method (instrument, survey or diagnostic criteria used) and data source (self-reported versus ascertained from administrative sources); (3) representativeness of study population; (4) control for confounding; (5) selection bias (risk of selection bias, based on percentage follow-up for longitudinal study designs and percentages of cases and controls for which exposure data could be ascertained for case–control designs); and (6) reverse causation (evaluated through study design and opportunity for recall bias (case–control studies)). Based on this extracted information, we created a series of binary covariates to capture potential sources of systematic bias within our input dataset. Across all risk–outcome pairs analyzed, the standard set of covariates included those measuring the representativeness of the study sample; whether the study sample represented a subpopulation only; risk of selection bias, defined as loss to follow-up or percentage for whom data not ascertained >20%; risk of reverse causation (case–control designs); measurement of ‘ever’ health outcome rather than current/recent; estimates uncontrolled for confounding; and odds ratio as an estimate of association (Supplementary Table 25).

Based on observed patterns in the input data, we additionally included two bias covariates for intimate partner violence analyses that captured the level of control for confounding within input studies, resulting in three categories of control for confounding: controlled for age and an additional three or more confounders (referent); controlled for age and one to two other confounders; uncontrolled for age, regardless of other confounders (Supplementary Table 26). Our intimate partner violence models additionally included two study-level bias covariates marking component exposure definitions and aggregate exposure definitions (Supplementary Table 23).

For childhood sexual abuse analyses, we additionally included bias covariates that captured whether a study reported on women only or men only and the level of control for confounding within input studies (whether a study controlled for age, gender and confounders beyond age and gender; Supplementary Table 27). Our childhood sexual abuse models additionally included two study-level bias covariates related to upper bounds of the age of exposure included in author case definitions and restricted perpetrator definitions used in author case definitions (Supplementary Table 24).

We additionally consulted with cause-specific research teams at the Institute for Health Metrics and Evaluation to gather expert guidance on accepted case definitions and best practices for measuring the relevant health outcome to inform outcome-specific bias covariates (for example, use of diagnostic interview versus symptom scale for measuring major depressive disorder; Supplementary Table 28).

The potential effect of bias covariates was tested using MR–BRT’s automated covariate selection process, which uses a Lasso strategy to identify statistically significant covariates at a threshold of 0.05 (ref. ^21^). Notably, for a covariate to be tested using this approach, there must be at least two rows of data for each value of the covariate (0 and 1). Owing to the small number of studies in our input datasets for several risk–outcome pairs, a limited set of bias covariates met the testing criteria (Supplementary Information 6). Covariates selected as significant by the stepwise Lasso strategy were adjusted for in the final model used to produce RR estimates.

#### Quantifying between-study heterogeneity

The MR–BRT tool quantifies between-study heterogeneity by accounting for within-study correlation, between-study heterogeneity and small number of studies. In this approach, the between-study heterogeneity parameter γ is estimated using the Fisher information matrix and the final uncertainty estimate reflects both the posterior uncertainty corresponding to the fixed effect (as in traditional meta-analytical approaches) as well as the 95th quantile of γ, which is sensitive to the number of studies and reported uncertainty of the effect size^21^.

#### Evaluating publication bias

Publication and reporting bias in the input data was detected and reported according to Egger’s regression test^50^, which assesses the degree to which the s.e.m. is correlated with effect size, in addition to visual inspection of funnel plots plotting the residuals of the risk function versus s.d.

#### Estimating the minimum risk exposure level

The theoretical minimum risk exposure level is the theoretically possible level of exposure that would minimize disease risk of the outcome, which, for intimate partner violence and childhood sexual abuse, was set at zero.

#### Estimating the burden of proof risk function

We estimated the BPRF, reflecting the most conservative estimate of the harmful association between intimate partner violence and childhood sexual abuse and the selected health outcomes that is consistent with the available evidence. For dichotomous risk factors, the BPRF is estimated as the fifth quantile of the model results, inclusive of between-study heterogeneity for harmful risks. ROSs are calculated from the BPRF as the signed log(BPRF) divided by two. A large positive ROS indicates strong and consistent evidence of an association, whereas a negative ROS suggests weak evidence of an association when accounting for between-study heterogeneity. BPRF values can be converted into measures of excess risk (Supplementary Information 7), which quantifies the additional risk of developing a health outcome due to exposure to the risk factor. ROS can be further categorized into star rating categories ranging from zero to five based upon the estimated ROS (one star, ≤0.0 ROS; two stars, >0.0–0.14 ROS; three stars, >0.14–0.41 ROS; four stars, >0.41–0.62 ROS; and five stars, >0.62 ROS). A one-star rating indicates weak evidence of association, whereas a five-star rating indicates very strong evidence, and all risk–outcome pairs receiving a one- to five-star rating are eligible for inclusion in GBD. Conversely, a zero-star rating is assigned when the lower bound of the 95% UI that does not incorporate γ (between-study heterogeneity) crosses the null RR value of one. This result indicates insufficient evidence of an association between exposure and outcome and ROS values are not calculated for these risk–outcome pairs. Risk–outcome pairs with a zero-star association do not satisfy GBD inclusion criteria.

#### Model validation

The meta-analytical tool used here has been extensively evaluated and validated by Zheng and colleagues^21^. For the range of risk–outcome pairs presented here, we undertook several additional sensitivity analyses to evaluate our main results. Across all risk–outcome pairs for which our input modeling dataset was more than ten observations, we undertook a sensitivity analysis in which we did not apply 10% trimming. For certain intimate partner violence-related outcomes, we accepted author definitions encompassing psychological intimate partner violence in addition to physical and/or sexual intimate partner violence. To assess the specificity of our model results to physical and/or sexual intimate partner violence (the forms of the violence currently included in the referent GBD case definition), we undertook a priori sensitivity analyses restricting to studies that only used an author definition involving physical and/or sexual violence (excluding author definitions that also incorporated psychological violence).We additionally undertook several outcome-specific analyses in which we investigated the impact of excluding studies with certain characteristics identified a priori or via bias covariate selection (Supplementary Information 1.2 and 1.5).

### Reporting summary

Further information on research design is available in the Nature Portfolio Reporting Summary linked to this article.

## Online content

Any methods, additional references, Nature Portfolio reporting summaries, source data, extended data, supplementary information, acknowledgements, peer review information; details of author contributions and competing interests; and statements of data and code availability are available at 10.1038/s41591-023-02629-5.

### Supplementary information

Supplementary Information Supplementary Sections 1–7 and Tables 1–30.

Reporting Summary

## Data Availability

The findings from this study are supported by data from the published literature. Details on data sources can be found on the burden of proof visualization tool (https://vizhub.healthdata.org/burden-of-proof/), including information about the data provider and links to where the data can be accessed or requested (where available). Study characteristics for all input data used in the analyses are also provided in Supplementary Information 1.

## Code availability

All code used for these analyses is publicly available online (https://github.com/ihmeuw-msca/burden-of-proof/).
