# Supplementary material for: Health effects associated with exposure to intimate partner violence against women and childhood sexual abuse: a Burden of Proof study
Source: Nat Med. 2023 Dec 11;29(12):3243–58. doi: 10.1038/s41591-023-02629-5 (PMC10719101; doi:10.1038/s41591-023-02629-5)
Supplement: Supplementary file 1 — Supplementary Information Supplementary Sections 1–7 and Tables 1–30. [file 41591_2023_2629_MOESM1_ESM.pdf]

nature medicine

Article

https://doi.org/10.1038/s41591-023-02629-5

Health effects associated with exposure to intimate partner violence against women and childhood sexual abuse: a Burden of Proof study

In the format provided by the authors and unedited

Contents

Section 1: Data inputs 4
Section 1.1: Data inputs for intimate partner violence studies 4
Section 1.2: Data selection and sensitivity analyses for intimate partner violence 9
Intimate partner violence and major depressive disorder 9
Intimate partner violence and maternal abortion and miscarriage 10
Intimate partner violence and HIV/AIDS 10
Intimate partner violence and anxiety disorders 11
Intimate partner violence and self-harm 11
Section 1.3: Descriptive results for additional health-related outcomes considered in association with exposure to intimate partner violence. 12
Maternal health outcomes 12
Heavy episodic drinking and alcohol use disorder 13
Section 1.4: Data inputs for childhood sexual abuse studies 14
Table S8: Summary characteristic of studies analyzed for childhood sexual abuse and 15 identified health outcomes. 14
Section 1.5: Data selection and sensitivity analyses for childhood sexual abuse 35
Childhood sexual abuse and alcohol use disorders 35
Childhood sexual abuse and self-harm 36
Childhood sexual abuse and major depressive disorder 37
Childhood sexual abuse and anxiety disorders 38
Childhood sexual abuse and drug use disorders 39
Section 1.6: Supplementary results for childhood sexual abuse selected outcomes 40
Type 2 diabetes mellitus 40
HIV/AIDS 41
Maternal abortion and miscarriage 41
Sexually transmitted infections excluding HIV ..... **Error! Bookmark not defined.**
Conduct disorder 41
Bulimia nervosa 41
Schizophrenia 41
Section 1.7: Descriptive results for additional health-related outcomes studied in association with childhood sexual abuse exposure. 42
High body mass index 42

Smoking ... 42
High systolic blood pressure ... 43
Section 2: Data input details for each risk-outcome pair ... 43
Table S15. Summary results of included intimate partner violence studies. ... 44
Table S16. Summary results of included childhood sexual abuse studies. ... 48
Section 3: PRISMA and GATHER ... 64
Section 3.1: PRISMA ... 64
Table S17. PRISMA 2020 abstract checklist ... 64
Table S18. PRISMA 2020 checklist ... 64
Section 7.2: GATHER ... 68
Table S19: GATHER Checklist ... 68
Section 4: Data source identification and assessment ... 70
Section 4.1: Literature searches ... 70
PubMed Search String ... 70
Embase Search String ... 71
Cumulative Index to Nursing and Allied Health Literature (CINAHL) Search String ... 72
PsycINFO Search String ... 73
Global Index Medicus Search String ... 75
Cochrane Search String ... 76
Web of Science Core Collection Search String ... 77
Section 4.2: Inclusion and Exclusion Criteria ... 77
Inclusion Criteria ... 78
Exclusion criteria ... 78
Section 4.3: Systematic review and meta-analysis citation searching ... 79
Section 4.4: Data Extraction ... 79
Table S20: Data Extraction Template ... 79
Section 4.5: Exposure and Outcome Definitions ... 84
Table S21: Definitions of included risk factors. ... 84
Table S22: Definitions of included outcomes. ... 84
Section 4.5.1: Additional description of accepted definitions and measurement tools for depressive and anxiety disorders ... 86
Section 4.5.2: Additional description of accepted definitions for substance use disorders ... 87
Section 5: Supplementary Methods ... 88

Section 5.1: Definitions of bias covariates 88
Table S26. Bias covariates for control for confounding used in intimate partner violence analyses. 90
Section 5.2: Data adjustment for non-mutually exclusive observations from a single study 93
Section 6: Study Quality and Bias Assessment 94
Table S29: Study bias characteristics for intimate partner violence. 94
Table S30a: Study bias Characteristics for childhood sexual abuse 98
Table S30b: Study bias Characteristics for childhood sexual abuse (continued) 106
Section 7: Calculating the ROS and measure of excess risk 115

Section 1: Data inputs

Section 1.1: Data inputs for intimate partner violence studies

Table S1: Summary characteristics of studies analyzed for intimate partner violence and five identified health outcomes.

Study	Location Name	Study Design	Years	Age Range	Mean Age (SD)	Total Sample Size	Follow-Up Years	Exposure Recall Type	Exposure Assessment Instrument	Violence Type	Outcome	Outcome Assessment Method	Outcome Assessment Instrument	Outcome Definition
Abdollahi 2015	Iran (Islamic Republic Of) (Mazandaran)	Prospective Cohort	2010	18-45	N/A	1,461	0.5	During Pregnancy	WHO Domestic Violence Questionnaire	Physical	Maternal Abortion and Miscarriage	administrative medical records or disease registries	Hospital or Health Clinic Administrative Records	Pregnancy loss determined via administrative records
Ackard 2007	United States of America (Minnesota)	Retrospective Cohort	1999-2004	N/A	20.4(0.8)	822	5	Prior to The Past One Year	Study-Specific Questionnaire	Physical and/or Sexual	Suicide and Self-Harm	self-report	Study-Specific Questionnaire (Project EAT survey – 'have you ever tried to kill yourself?)	Lifetime Experience of Suicide Attempt
Ahmadabadi 2020	Australia	Prospective Cohort	1981-2014	30-30	N/A	891	30	Lifetime	Composite Abuse Scale (CAS)	Physical and/or Sexual	Anxiety Disorders; Depressive Disorders	self-report	Composite International Diagnostic Interview (CIDI)	Lifetime Anxiety or Depressive Disorders Determined by DSM-IV Criteria
Ali 2009	Pakistan (Sindh)	Case-Control	2007-2007	15-48	N/A	304	N/A	Lifetime	Study specific questionnaire	Physical and/or Psychological	Depressive Disorders	self-report; physician diagnosis	Diagnosed with depression by psychiatrist or family physician according to DSM-IV criteria *and* currently having score of 8 or higher on Self Reporting	Lifetime Diagnosis of Major Depressive Disorder

Table S1: Summary characteristics of studies analyzed for intimate partner violence and five identified health outcomes.

Study	Location Name	Study Design	Years	Age Range	Mean Age (SD)	Total Sample Size	Follow-Up Years	Exposure Recall Type	Exposure Assessment Instrument	Violence Type	Outcome	Outcome Assessment Method	Outcome Assessment Instrument	Outcome Definition
													Questionnaire (SRQ) 20	
Bourassa 2007	Canada	Case-Control	2001-2003	N/A	25.3	1,003	N/A	Past Year	Abuse Assessment Screen	Physical and/or Sexual	Maternal Abortion and Miscarriage	administrative medical records or disease registries	Hospital or Health Clinic Administrative Records	Elective abortion
Brown 2020	Australia	Prospective Cohort	2003-2015	28-60	N/A	1,507	10	Past Year	Composite Abuse Scale (CAS)	Physical and/or Sexual and/or Psychological	Anxiety Disorders; Depressive Disorders	self-report	Beck Anxiety Inventory (BAI); Center for Epidemiologic Studies Depression Scale (CES-D	Anxiety Disorders Determined by BAI Score >=16; Major Depressive Disorder Determined by CES-D Score >=20
Catak 2016	Turkey	Case-Control	2011-2011	15-49	N/A	752	N/A	Unknown	Study-Specific Questionnaire	Physical	Maternal Abortion and Miscarriage	physician diagnosis	Hospital or Health Clinic Administrative Records	Spontaneous Abortion
Chowdhary 2008	India (Goa)	Prospective Cohort	2001-2004	18-50	N/A	1,537	1	Lifetime	Study-Specific Questionnaire	Physical and/or Sexual	Suicide and Self-Harm; Depressive Disorders; Anxiety Disorders	self-report; biomarker	Clinical Interview Schedule-Revised (CIS-R)	Lifetime Experience of Suicide Attempt; Current Depression Determined by CIS-R Criteria
Delong 2019	South Africa	Longitudinal Data from A Randomized Controlled Trial	2011-2017	13-20	N/A	2,366	3	Lifetime	WHO Violence Against Women Questionnaire	Physical and/or Sexual	Human Immunodeficiency Virus (HIV)	biomarker	HIV serostatus confirmed by blood / biomarker test	HIV Positive Serostatus
Deyessa 2018	Ethiopia (Addis Ababa)	Case-Control	2014-2014	15-49	N/A	510	N/A	Lifetime	Study-Specific Questionnaire	Physical and/or Sexual	Human Immunodeficiency Virus (HIV)	biomarker	HIV serostatus confirmed by blood / biomarker test	HIV Positive Serostatus
Ehrensaft 2006	New Zealand	Prospective Cohort	1972-1998	N/A	26	449	8	Past 3 Years	Partner Conflict Calendar (PCC)	Physical	Anxiety Disorders; Posttraumatic Stress	self-report	Diagnostic Interview	Past Year Diagnosis of GAD

Table S1: Summary characteristics of studies analyzed for intimate partner violence and five identified health outcomes.

Study	Location Name	Study Design	Years	Age Range	Mean Age (SD)	Total Sample Size	Follow-Up Years	Exposure Recall Type	Exposure Assessment Instrument	Violence Type	Outcome	Outcome Assessment Method	Outcome Assessment Instrument	Outcome Definition
											disorder; Depressive disorders		Schedule (DIS) or Diagnostic Interview Schedule-IV (DIS-IV)	or PTSD; Current Depression Determined by DIS or DIS-IV
Fonck 2005	Kenya (Nairobi)	Other (Cross-Sectional)	1996-1997	0-99	N/A	520	N/A	Lifetime	Study-Specific Questionnaire	Physical	Human Immunodeficiency Virus (HIV)	biomarker	HIV serostatus confirmed by blood / biomarker test	HIV Positive Serostatus
Han 2019	Republic Of Korea	Prospective Cohort	2006-2007	19-99	N/A	3,052	1	Past Year	Conflict Tactics Scale (CTS)	Physical	Depressive Disorders	self-report	Center for Epidemiologic Studies Depression Scale (CES-D)	Depression Determined By CES-D Score >=16
Ibrahim 2015	Egypt	Prospective Cohort	2010-2012	18-43	N/A	1,857	N/A	During Pregnancy	Study-Specific Questionnaire	Physical	Maternal Abortion and Miscarriage	administrative medical records or disease registries	Hospital or Health Clinic Administrative Records	Abortion past 20 weeks gestation
Jewkes 2010	South Africa (Eastern Cape)	Prospective Cohort	2002-2006	16-23	N/A	1,099	2	Lifetime	WHO Violence Against Women Questionnaire	Physical and/or Sexual	Human Immunodeficiency Virus (HIV)	biomarker	HIV serostatus confirmed by blood / biomarker test	HIV Positive Serostatus
Johri 2011	Guatemala	Case-Control	2006-2006	15-49	N/A	1,897	N/A	Past Year	WHO Domestic Violence Questionnaire	Physical and/or Sexual	Maternal Abortion and Miscarriage	physician diagnosis	Hospital or Health Clinic Administrative Records	Pregnancy loss before 28 weeks gestation
Kaslow 2000	United States of America	Case-Control	1995-1997	18-64	30.8(8.96)	285	N/A	Lifetime	Index of Spouse Abuse (ISA)	Physical	Suicide and Self-Harm	administrative medical records or disease registries	Hospital Administrative Records	Suicide Attempt Determined With Hospital Admittance
Kouyoumdjian 2013	Uganda	Prospective Cohort	2000-2009	15-49	N/A	10,252	5.5	Lifetime	Conflict Tactics Scale modified version (CTS2)	Physical and/or Sexual	Human Immunodeficiency Virus (HIV)	biomarker	HIV serostatus confirmed by blood / biomarker test	HIV Positive Serostatus

Table S1: Summary characteristics of studies analyzed for intimate partner violence and five identified health outcomes.

Study	Location Name	Study Design	Years	Age Range	Mean Age (SD)	Total Sample Size	Follow-Up Years	Exposure Recall Type	Exposure Assessment Instrument	Violence Type	Outcome	Outcome Assessment Method	Outcome Assessment Instrument	Outcome Definition
Leung 2002	China (Hong Kong Special Administrative Region of China)	Case-Control	1999-1999	N/A	27(8)	501	N/A	Past Year	Abuse Assessment Screen	Physical and/or Sexual	Maternal Abortion and Miscarriage	administrative medical records or disease registries	Hospital or Health Clinic Administrative Records	Elective abortion
Llosamartínez 2019	Uruguay	Case-Control	2014-2015	N/A	28.8(1)	58	N/A	Past Year	Woman Abuse Screening Tool (Short Version)	Physical and/or Sexual and/or Psychological	Depressive Disorders	self-report	Beck Depression Inventory (BDI)	Depression Determined By BDI Score >17
Makaroun 2020	United States of America	Prospective Cohort	2014-2016	45+	N/A	4,481	1.67	Past Year	Hurt, Insult, Threaten, Scream (E-HITS) Tool	Physical and/or Sexual	Suicide and Self-Harm; Anxiety Disorders; Depressive disorders	administrative medical records or disease registries	Analysis of Hospital or Health Clinic and Administrative Data	Suicide Attempt or Self Harm Determined with Health Administrative Records; Lifetime Diagnosis of Anxiety Disorder or Depression
Maman 2002	United Republic of Tanzania	Prospective Cohort	1999	18-99	N/A	245	0.25	Lifetime	Conflict Tactics Scale (CTS)	Physical and/or Sexual	Human Immunodeficiency Virus (HIV)	biomarker	HIV serostatus confirmed by blood / biomarker test	HIV Positive Serostatus
Nelson 2003	United States Of America (Pennsylvania)	Case-Control	1999-2001	14-40	N/A	1,199	N/A	Lifetime	Index of Spousal Abuse	Physical	Maternal Abortion and Miscarriage	self-report; administrative medical records or disease registries	Follow-up telephone interviews; Hospital or Health Clinic Administrative Records	Pregnancy loss before 22 weeks gestation
Ouellet-Morin 2015	Multiple Locations	Prospective Cohort	1994-2001	N/A	40	978	7	Unknown	Conflict Tactics Scale (CTS)	Physical and/or Sexual and/or Psychological	Depressive Disorders	self-report	Diagnostic Interview Schedule (DIS); Diagnostic Interview Schedule-IV (DIS-IV)	Depression Determined by BDI Score >19

Table S1: Summary characteristics of studies analyzed for intimate partner violence and five identified health outcomes.

Study	Location Name	Study Design	Years	Age Range	Mean Age (SD)	Total Sample Size	Follow-Up Years	Exposure Recall Type	Exposure Assessment Instrument	Violence Type	Outcome	Outcome Assessment Method	Outcome Assessment Instrument	Outcome Definition
Pico-Alfonso 2006	Spain	Case-Control	2000-2002	N/A	44.55	182	N/A	Lifetime	N/A	Physical and/or Psychological	Depressive Disorders	self-report	Beck Depression Inventory (BDI)	Depression Determined by BDI Score >19
Romito 2009	Italy (Friuli-Venezia Giulia)	Case-Control	2006-2007	30-99	N/A	883	N/A	Past Year	Study-Specific Questionnaire	Physical and/or Sexual	Maternal Abortion and Miscarriage	administrative medical records or disease registries	Hospital or Health Clinic Administrative Records	Elective abortion occurring before 12 weeks gestation
Suglia 2011	United States Of America	Prospective Cohort	1996-2000	N/A	24.8(5.9)	2,030	3	Prior to The Past One Year	Study-Specific Questionnaire	Physical and/or Sexual	Anxiety Disorder; Depressive Disorders	self-report	Composite International Diagnostic Interview (CIDI)	Anxiety Disorders Determined by DSM-IV Criteria; Depression Determined by CIDI Score >3
Taft 2008	Australia	Prospective Cohort	1996-2000	22-27	N/A	9,333	4	Lifetime	Conflict Tactics Scale (CTS)	Physical and/or Sexual	Depressive Disorders	self-report	Center for Epidemiologic Studies Depression Scale (CES-D)	Depression Determined By CES-D Score >=10
Taft 2019	Australia	Prospective Cohort	1996-2009	18-36	N/A	9,021	4	Prior to The Past One Year	Study-Specific Questionnaire	Physical and/or Sexual and/or Psychological	Maternal Abortion and Miscarriage	self-report	Australian Longitudinal Study on Women's Health Study Questionnaire (asks participants how many times they have experience stillbirth, miscarriage, and abortion)	Abortion for reason other than medical abnormality

Table S1: Summary characteristics of studies analyzed for intimate partner violence and five identified health outcomes.

Section 1.2: Data selection and sensitivity analyses for intimate partner violence

Intimate partner violence and major depressive disorder

For major depressive disorder, we extracted 14 studies reporting an association with intimate partner violence (IPV). Among these studies, we identified two reporting on the Fragile Families and Child Wellbeing Study. We retained estimates from Suglia 2011 because the author exposure definitions more closely aligned with the GBD case definition of IPV. Suglia 2011 used the composite international diagnostic interview short form (CIDI-SF) during a follow-up interview. While the CIDI-SF is not typically an accepted symptom scale within the GBD, upon review of this specific study cohort (Fragile Families and Child Wellbeing Study) in consultation with the mental health team at IHME, the use of the CIDI-SF was accepted. We additionally identified two studies reporting on the Veteran's Health Association Corporate Data Warehouse. We chose to retain estimates from Makaroun 2020 as the authors reported estimates specific to physical and/or sexual IPV while only an aggregate measure of IPV (including physical, sexual, and psychological violence) was available from the other study. These selections resulted in a total of 12 studies which formed our modeling input dataset.

Using these data, we undertook three sensitivity analyses. First, we ran a sensitivity analysis to test the impact of applying 10% trimming on model results. Without trimming 10% of outliers, results indicated greater between study heterogeneity ($\gamma = 0.039$) and 0.0698 risk-outcome score (ROS).

Second, existing evidence from longitudinal studies has indicated that the relationship between intimate partner violence and depression may be bi-directional. Thus, we ran a sensitivity analysis using identified cohort studies only (excluding three case-control studies without control for baseline depression; Table S2). Model results from using a subset of cohort studies were similar to our main analysis (0.222 ROS).

Lastly, to assess the impact of including these author definitions which included exposure to psychological partner violence, we undertook a sensitivity analysis restricting to studies which only used an author definition which involved physical and/or sexual abuse (including those assessing physical violence only and sexual violence only). The results of this sensitivity analysis did not substantially differ from the main result (0.194 ROS; Table S2).

Table S2. Sensitivity analyses for intimate partner violence and major depressive disorder.

**Analysis**	**RR and 95%UI**	**ROS**	**Pub. Bias**	**Gamma**	**No. of studies (no. observations)**	**Selected bias covariates**
**Main**	2.09 (1.55-2.82)	0.243	0	1.06E-06	12 (16)	--
**0% trimming**	2.07 (1.03-4.19)	0.0698	0	0.039	12 (16)	--
**Exclude case-control studies**	2.01 (1.48-2.72)	0.222	0	3.33E-06	9 (12)	--

**Exclude studies using exposure definitions which include psychological violence**	1.91 (1.4-2.61)	0.194	0	1.61E-05	7 (10)	--

MDD = Major Depressive Disorder; Pub. = publication; no. = number of; ROS = risk-outcome score.

Intimate partner violence and maternal abortion and miscarriage

For maternal abortion and miscarriage, we ran two sensitivity analyses. First, we tested the effect of trimming within our main model by undertaking an analysis without trimming outliers. In our model run without trimming two outlying studies, the estimated between-study heterogeneity was much higher than in our main results (γ = 0.385) and resulting negative ROS (-0.341). Our second analysis tested the effect of excluding a study (Abdollahi 2015) which reported estimates from a sample with fewer than 10 cases of abortion/miscarriage in exposed and/or unexposed groups. The results of this analysis also showed a greater between-study heterogeneity (γ = 0.14) and -0.169 ROS (Table S3).

Table S3. Sensitivity analyses for intimate partner violence and maternal abortion and miscarriage.

**Analysis**	**RR and 95%UI**	**ROS**	**Pub. Bias**	**Gamma**	**No. of studies (no. observations)**	**Selected bias covariates**
**Main**	2.03 (1.25-3.31)	0.15	0	6.19E-06	9 (11)	Current and/or recent IPV
**0% trimming**	2.41 (0.375-15.5)	-0.341	0	0.385	9 (11)	Current and/or recent IPV
**Exclude studies with fewer than 10 cases in exposed / unexposed groups**	2.01 (0.584-6.9)	-0.169	0	0.14	8 (10)	Current and/or recent IPV

Pub. = publication; no. = number of; ROS = risk-outcome score.

Intimate partner violence and HIV/AIDS

For HIV/AIDS, we undertook two sensitivity analyses. First, we tested the impact of applying 10% trimming on model results and found that results were not substantially different from our main analysis (with trimming: 0.0399 ROS). Second, because certain evidence suggests that HIV serostatus and/or the disclosure of one's serostatus may precipitate consequent IPV, we ran a second sensitivity analysis restricted to prospective cohort studies. Importantly, in our study identification process, we only accepted study designs in which exposure to violence preceded incident HIV infection (prospective cohorts) *or* designs in which study participants were unaware of their serostatus at the time that they reported exposure(s) to IPV, thus removing the possibility that serostatus may have caused exposure. Nonetheless, longitudinal cohort studies which measure HIV incidence using biomarkers provide the best evidence for a causal relationship. Our results estimated using only prospective cohort studies were consistent with our main analysis (0.0568 ROS; Table S4).

Table S4. Sensitivity analyses for intimate partner violence and HIV/AIDS.

Analysis	RR and 95%UI	ROS	Pub. Bias	Gamma	No. of studies (no. observations)	Selected bias covariates
**Main**	1.58 (1.06-2.34)	0.0626	0	2.48E-06	6 (9)	--
**10% trimming**	1.53 (1.01-2.31)	0.0399	0	2.94E-06	3 (4)	--
**Exclude study designs other than prospective cohorts**	1.56 (1.05-2.32)	0.0568	0	7.61E-10	6 (9)	--

Pub. = publication; no. = number of; ROS = risk-outcome score.

Intimate partner violence and anxiety disorders

For anxiety disorders, we undertook two sensitivity analyses. First, we ran a sensitivity analysis to test the impact of applying 10% trimming on model results. With trimming 10% of outliers, results were consistent with our main analyses, albeit slightly more uncertain (Table S5). Second, to assess the impact of including these author definitions which included exposure to psychological partner violence, we undertook a sensitivity analysis restricting to studies which only used an author definition which involved physical and/or sexual abuse (including those assessing physical violence only and sexual violence only). The results of this sensitivity analysis did not substantially differ from the main result (-0.0988 ROS; Table S5).

Table S5. Sensitivity analyses for intimate partner violence and anxiety disorders.

Analysis	RR and 95%UI	ROS	Pub. Bias	Gamma	No. of studies (no. observations)	Selected bias covariates
**Main**	2.57 (0.802-8.25)	-0.0166	0	0.0961	5 (8)	Current and/or recent IPV
**10% trimming**	2.39 (0.798-7.13)	-0.0247	0	0.0764	5 (8)	Current and/or recent IPV
**Exclude studies using exposure definitions which include psychological violence**	2.4 (0.668-8.61)	-0.0988	0	0.109	4 (7)	Study at risk of selection bias, Current and/or recent IPV

Pub. = publication; no. = number of; ROS = risk-outcome score.

Intimate partner violence and self-harm

For self-harm, we ran two sensitivity analyses. First, we tested the impact of applying 10% trimming on model results. With trimming a single outlying study, the overall estimated study-heterogeneity was much lower ($\gamma < 0.001$), resulting in a three-star association (0.171 ROS).

Second, we ran our model excluding one study which used an alternate outcome definition defined as suicidal ideation and/or behavior (Makaroun 2020; Table S6).

Table S6. Sensitivity analyses for intimate partner violence and self-harm.

Analysis	RR and 95%UI	ROS	Pub. Bias	Gamma	No. of studies (no. observations)	Selected bias covariates
**Main**	2.99 (0.295-30.3)	-0.424	0	0.343	4 (6)	--
**10% trimming**	4.63 (1.12-19.1)	0.171	0	3.98E-05	4 (6)	--
**Excluding one study using an alternate outcome definition of suicide ideation and/or behavior**	2.5 (0.159-39.3)	-0.697	0	0.429	3 (4)	--

Pub. = publication; no. = number of; ROS = risk-outcome score.

Section 1.3: Descriptive results for additional health-related outcomes considered in association with exposure to intimate partner violence.

While not meeting our three-study minimum, we additionally identified literature reporting the risk of specific maternal health outcomes in association with intimate partner violence, including maternal hypertensive disorders (n=2), gestational diabetes (n=2), maternal hemorrhage (n=2), alcohol use disorder (n=2) and sexually transmitted infection excluding HIV (n=2). Further, peripartum depression (n=8) and heavy episodic drinking (n=5) were well-studied health outcomes across extracted studies; however, as these outcomes are not yet included in GBD, we did not undertake a meta-analysis of these studies. Our consideration of these input studies are described further below.

Maternal health outcomes

We identified literature reporting the risk of maternal hypertensive disorders, gestational diabetes, and maternal hemorrhage; however, none of these outcomes met our minimum availability criteria of at least three studies identified. Peripartum depression was a well-studied outcome across extracted studies (n=8), and was defined as depression among pregnant or post-partum mothers up to one year after birth. All but one study used the Edinburgh Postnatal Depression Scale (EPDS) to measure peripartum depression; McMahon 2011 used the composite international diagnostic interview short form (CIDI-SF) during a follow-up interview at one-year post-partum. While the CIDI-SF is not typically an accepted symptom scale within the GBD, upon review of this specific study cohort (Fragile Families and Child Wellbeing Study) in consultation with the mental health team at IHME, the use of the CIDI-SF was accepted. Additionally, most studies measured IPV during pregnancy, although one measured lifetime occurrence of IPV while another measured any IPV experienced in the past year (Extended data figure 5).

Heavy episodic drinking and alcohol use disorder

Heavy episodic drinking is an outcome which does not correspond directly to the GBD cause of alcohol use disorder but reflects a pattern of heavy and/or harmful alcohol use. We extracted five studies which measured the risk of heavy episodic (binge) drinking in association with exposure to physical and/or sexual IPV. Case definitions for heavy drinking varied across studies (Table S7). In general, included studies did not show a strong association between heavy episodic drinking and IPV, with only one study showing a significantly harmful effect (Extended data figure 5). We also identified and extracted two studies which measured alcohol use disorder according to DSM criteria, which does map to the GBD cause of alcohol use disorder but did not meet our minimum availability criteria to undertake a BPRF analysis (i.e., at least three studies). Similar to the studies identified for heavy episodic drinking, these studies showed inconsistent results (Extended data figure 5).

Table S7: Case definitions for heavy episodic (binge) drinking by study.

Study	Outcome definition
Nowotny 2013	Binge drinking defined as drinking five or more drinks during a single occasion at least two to three times a month in the past year
Martino 2005	Past-month heavy alcoholic drinking (days on which they had four or more alcoholic drinks on the same occasion)
Gao 2010	Drank six or more alcoholic drinks on at least one occasion in the last 12 months
Gilbert 2012	Binge drinking (not further defined)
Dichter 2017	ICD-9 or 10 code for alcohol abuse

Section 1.4: Data inputs for childhood sexual abuse studies

Table S8: Summary characteristic of studies analyzed for childhood sexual abuse and 15 identified health outcomes.

Table S8: Summary characteristic of studies analyzed for childhood sexual abuse and 15 identified health outcomes.

Study	Location Name	Study Design	Years	Sex	Age Range	Mean Age (SD)	Total Sample Size	Exposure Recall Type	Exposure Assessment Method	Exposure Assessment Instrument	Age of Exposure	Perpetrator	Outcome	Outcome Assessment Method	Outcome Assessment Instrument	Outcome Definition
Abajobir 2017	Australia	Prospective Cohort	1981-2004	Both	21-21	N/A	3,762	Lifetime	Administrative records	Administrative Records	0-14	Any	Asthma	self-report; physician diagnosis	Self-reported diagnosis of Asthma by a physician (ever been told by a physician that they had asthma)	Lifetime Diagnosis of Asthma
Abajobir 2018	Australia	Prospective Cohort	1981-2004	Female	19-22	N/A	1,980	Lifetime	Administrative records	Administrative Records	0-14	Any	Maternal Abortion and Miscarriage	self-report	Study-Specific Questionnaire (survey items where participants asked to report number of times they experienced pregnancy, termination of pregnancy, and miscarriage)	Lifetime Experience of Miscarriage
Andrews 1995	United Kingdom	Prospective Cohort	1980-1989	Female	15-28	N/A	101	Lifetime	Self-reported	Study-Specific Questionnaire	0-17	Any	Bulimia Nervosa; Depressive Disorders	self-report	Present State Examination (PSE)	Bulimia Nervosa or Past Year Major Depressive Disorder Determined by DSM-III Criteria
Banyard 2004	United States of America	Prospective Cohort	1973-1990	Male	N/A	26.5	106	Lifetime	Self-reported; Administrative records	Administrative Records; Study-Specific Questionnaire	0-17	Any	Suicide And Self-Harm	self-report	Study-Specific Questionnaire (survey item asking the participant if he or she had ever intentionally tried to take his or her life)	Lifetime Experience of Suicide Attempt

Table S8: Summary characteristic of studies analyzed for childhood sexual abuse and 15 identified health outcomes.

Study	Location Name	Study Design	Years	Sex	Age Range	Mean Age (SD)	Total Sample Size	Exposure Recall Type	Exposure Assessment Method	Exposure Assessment Instrument	Age of Exposure	Perpetrator	Outcome	Outcome Assessment Method	Outcome Assessment Instrument	Outcome Definition
Bentivegna 2022	United Kingdom	Prospective Cohort	2000-2019	Female ; Male	17-17	N/A	5,119 (F); 4,852 (M)	Past Year	Self-reported	Study-Specific Questionnaire	17-17	Any	Suicide And Self-Harm	self-report	Study-Specific Questionnaire (participants asked whether they hurt themselves on purpose in the previous 12 months by cutting, burning, or bruising, or by taking an overdose, pulling out their hair, or hurting themselves in other ways)	Past Year Self Harm
Bifulco 1991	United Kingdom (Islington)	Prospective Cohort	1989-1991	Female	18-50	N/A	286	Lifetime	Self-reported	Study-Specific Questionnaire	0-17	Any	Depressive Disorders	self-report	Present State Examination (PSE)	Past Three Years Depression Determined By PSE
Borges 2021	Mexico (Mexico City)	Prospective Cohort	2005-2013	Both	19-26	N/A	1,054	Lifetime	Self-reported	Composite International Diagnostic Interview (CIDI)	0-18	Any	Suicide And Self-Harm; Alcohol Abuse or Dependence; Substance Abuse or Dependence	self-report	Composite International Diagnostic Interview (CIDI)	Suicide Attempt Since Baseline Survey; Alcohol Dependence or Substance Use Disorders Determined by DSM-III Criteria
Brown 1999	United States of America (New York)	Prospective Cohort	1975-1992	Both	18-99	N/A	580	Lifetime	Self-reported; Administrative records	Administrative Records	0-18	Any	Suicide And Self-Harm	self-report	Study-Specific Questionnaire ("suicide attempts were reported and described by children with regard to method, frequency, and associated treatment")	Suicide Attempt At Ages >=18

Table S8: Summary characteristic of studies analyzed for childhood sexual abuse and 15 identified health outcomes.

Study	Location Name	Study Design	Years	Sex	Age Range	Mean Age (SD)	Total Sample Size	Exposure Recall Type	Exposure Assessment Method	Exposure Assessment Instrument	Age of Exposure	Perpetrator	Outcome	Outcome Assessment Method	Outcome Assessment Instrument	Outcome Definition
Chapman 2004	United States of America (California)	Retrospective Cohort	1995-1996	Female; Male	N/A	56.6	9460	Lifetime	Self-reported	ACE Study Questionnaire	0-18	Any	Depressive Disorders	self-report	Diagnostic Interview Schedule (DIS); Center for Epidemiologic Studies Depression Scale (CES-D)	Past Year Depression Determined by Study Specific Criteria
Chatziioannidis 2019	Greece	Case-Control	2015-2019	Both	0-65	40(10)	124	Lifetime	Self-reported	Childhood Experience of Care and Abuse Questionnaire (CECAQ)	0-18	Any	Schizophrenic And Psychotic Disorders	physician diagnosis	Diagnosis of Schizophrenia based on chart review and confirmed with use of Greek Version of the Mini-International Neuropsychiatric Interview 5.0.0 (MINI)	Schizophrenia Determined by DSM-IV Criteria
Cheasty 1998	Ireland	Case-Control	1996-1997	Female	18-87	N/A	237	Lifetime	Self-reported	Study-Specific Questionnaire	0-16	Any	Depressive Disorders	self-report; physician diagnosis	Screening questionnaire (not specified) and Beck Depression Inventory (BDI)	Depression Determined by initial screening quesitonnaire and confirmed by cut off score of 12 on BDI
Chen 2014	China	Case-Control	2009-2011	Female	30-60	N/A	11,115	Lifetime	Self-reported	Study-Specific Questionnaire	0-16	Any	Suicide And Self-Harm	self-report	Composite International Diagnostic Interview (CIDI)	Lifetime Experience of Suicide Attempt or Suicidal Ideation
Cohen 2001	United States of America (New York)	Prospective Cohort	1975-1994	Both	18-29	N/A	664	Lifetime	Self-reported; Administrative records	Administrative Records; Study-Specific Questionnaire	0-18	Non-Partner	Anxiety Disorders; Depressive Disorders; Substance Abuse or Dependence	self-report	Diagnostic Interview Schedule for Children (DISC-1)	Anxiety Disorders, Depressive Disorders, or Substance Use Disorders Determined by DSM-III Criteria

Table S8: Summary characteristic of studies analyzed for childhood sexual abuse and 15 identified health outcomes.

Study	Location Name	Study Design	Years	Sex	Age Range	Mean Age (SD)	Total Sample Size	Exposure Recall Type	Exposure Assessment Method	Exposure Assessment Instrument	Age of Exposure	Perpetrator	Outcome	Outcome Assessment Method	Outcome Assessment Instrument	Outcome Definition
Comijs 2013	Netherlands	Case-Control	2007-2010	Both	60-93	N/A	508	Lifetime	Self-reported	Study-Specific Questionnaire	0-16	Any	Depressive Disorders	self-report	Composite International Diagnostic Interview (CIDI)	Depressive Disorders Determined by DSM-IV Criteria
Conroy 2009	Australia	Case-Control	2005	Female; Male	N/A	35	571 (F); 742 (M)	Lifetime	Self-reported	Study-Specific-Questionnaire	0-18	Any	Substance Abuse or Dependence	NA	Enrollment in a Pharmacotherapy program	Clinical Determination of Opioid Dependence
Coogan 2013	United States of America	Prospective Cohort	1995-2011	Female	21-99	N/A	28,456	Lifetime	Self-reported	Conflict Tactics Scale (CTS)	0-11	Any	Asthma	self-report	Self-reported physician's diagnosis of asthma	Diagnosis of Asthma at Ages >=18 or Use of Asthma Treatment >=3 Times/Week
Copeland 2018	United States of America (North Carolina)	Prospective Cohort	1993-2015	Both	19-30	N/A	1420	Lifetime	Self-reported; Parent Report	Child And Adolescent Psychiatric Assessment (CAPA)	0-16	Any	Anxiety Disorders; Depressive disorders	self-report	Young Adult Psychiatric Assessment (YAPA)	Anxiety Disorders and Depression at Ages >=18 Determined by DSM-IV criteria
Cutajar 2010	Australia	Case-Control	1964-1995	Female; Male	12-43	N/A	4,208 (F); 1,157 (M)	Lifetime	Administrative records	Administrative Records	0-18	Any	Alcohol Abuse or Dependence; Substance Abuse or Dependence; Schizophrenic And Psychotic Disorders	administrative medical records or disease registries	Administrative Records	Alcohol Abuse or Substance Abuse Documented in the Victorian Psychiatric Case Register (VPCR); Schizophrenia Disorder Determined by DSM-IV Criteria

Table S8: Summary characteristic of studies analyzed for childhood sexual abuse and 15 identified health outcomes.

Study	Location Name	Study Design	Years	Sex	Age Range	Mean Age (SD)	Total Sample Size	Exposure Recall Type	Exposure Assessment Method	Exposure Assessment Instrument	Age of Exposure	Perpetrator	Outcome	Outcome Assessment Method	Outcome Assessment Instrument	Outcome Definition
Demakakos 2020	United Kingdom (England)	Retrospective Cohort	2002-2007	Female	55-89	N/A	2,795	Lifetime	Self-reported	2007 Elsa Life History Interview	0-16	Any	Maternal Abortion and Miscarriage	self-report	Study-Specific Questionnaire (respondents were asked to remember all pregnancies that did not result in live birth including any experience of miscarriage, as a part of reproductive history interviews)	Lifetime Experience of 2 or More Miscarriages
Deyessa 2018	Ethiopia (Addis Ababa)	Case-Control	2014-2014	Female	15-49	N/A	510	Lifetime	Self-reported	Study-Specific Questionnaire	0-18	Any	Human Immunodeficiency Virus (HIV)	biomarker	HIV serostatus confirmed by blood / biomarker test	HIV Positive Serostatus
Dinwiddie 2000	Australia	Retrospective Cohort	1978-1993	Both	N/A	43	107 (F); 25 (M)	Lifetime	Self-reported	Semi-Structured Assessment for the Genetics of Alcoholism (SSAGA)	0-18	Any	Suicide And Self-Harm; Alcohol Abuse or Dependence; Anxiety Disorders; Conduct Disorders; Depressive Disorders	self-report	Semi-Structured Assessment for the Genetics of Alcoholism (SSAGA)	Lifetime Experience of Suicide Attempt; Alcohol Use, Social Phobia, Conduct Disorder, or Major Depressive Disorder Determined by DSM-III Criteria

Table S8: Summary characteristic of studies analyzed for childhood sexual abuse and 15 identified health outcomes.

Study	Location Name	Study Design	Years	Sex	Age Range	Mean Age (SD)	Total Sample Size	Exposure Recall Type	Exposure Assessment Method	Exposure Assessment Instrument	Age of Exposure	Perpetrator	Outcome	Outcome Assessment Method	Outcome Assessment Instrument	Outcome Definition
Dong 2004	United States of America (California)	Retrospective Cohort	1995-1997	Both	N/A	56(15.2)	17,337	Lifetime	Self-reported	Conflict Tactics Scale (CTS); Childhood Trauma Questionnaire (CTQ)	0-18	Any	Cardiovascular Diseases (CVD)	self-report	Standardized medical examination (history of ischemic heart disease defined as a positive response to any of 3 questions: “have you had or ever been told you have a heart attack (coronary)?”, “Do you get pain or heavy pressure in your chest with exertion?”, “do you use nitroglycerine?”)	Self-reported history of ischemic heart disease
Dube 2005	United States of America (California)	Retrospective Cohort	1995-1997	Female; Male	N/A	56(15.2)	9367 (F); 7970 (M)	Lifetime	Self-reported	Study-Specific Questionnaire	0-18	Any	Suicide And Self-Harm; Depressive Disorders	self-report	Study-Specific Questionnaire (respondents asked if ever attempted to commit suicide)	Lifetime Experience of Suicide Attempt; Depression Determined By Study Specific Criteria
Duncan 2008	United States of America	Retrospective Cohort	2002-2003	Both	15-30	22.64(4.44)	819	Before 16	Self-reported	semi-structured assessment for the genetics of alcoholism (SSAGA) and adaptation of the trauma assessment from the national comorbidity study	0-16	Any	Substance Abuse or Dependence	self-report	Semi-Structured Assessment for the Genetics of Alcoholism (SSAGE)	Cannabis Use or Dependence Determined by DSM-IV Criteria

Table S8: Summary characteristic of studies analyzed for childhood sexual abuse and 15 identified health outcomes.

Study	Location Name	Study Design	Years	Sex	Age Range	Mean Age (SD)	Total Sample Size	Exposure Recall Type	Exposure Assessment Method	Exposure Assessment Instrument	Age of Exposure	Perpetrator	Outcome	Outcome Assessment Method	Outcome Assessment Instrument	Outcome Definition
Duncan 2015	United States of America	Prospective Cohort	1994-2009	Female; Male	24-34	N/A	3,699	Lifetime	Self-reported	Study-Specific Questionnaire	0-12	Family Member or Caregiver	Diabetes (Type 2)	self-report; physician diagnosis; biomarker	Biomarker; Self-reported taking anti-diabetic medication; Report of receiving a diagnosis of diabetes or high blood glucose by a health care provider	HbA1c >=6.5%; Fasting Glucose >=126 mg/dL; Non fasting Blood Glucose >=200 mg/dL; or Lifetime Diagnosis of Diabetes
Ebert 2019	Belgium	Prospective Cohort	2014-2015	Both	18-99	N/A	2,242	Lifetime	Self-reported	Study-Specific Questionnaire	0-17	Any	Depressive Disorders	self-report	Composite International Diagnostic Interview (CIDI)	Depressive Disorders Determined by CIDI-SC Criteria
Enns 2006	Netherlands	Prospective Cohort	1996-1999	Both	18-64	N/A	5,670	Lifetime	Self-reported	Study-Specific Questionnaire	0-16	Anyone Or Not Specified	Suicide And Self-Harm	self-report	Composite International Diagnostic Interview (CIDI)	Lifetime Experience of Suicide Attempt
Fenton 2013	United States of America	Prospective Cohort	2001-2005	Both	18-99	N/A	27,712	Lifetime	Self-reported	Study-Specific Questionnaire	0-18	Family Member or Caregiver	Alcohol Abuse or Dependence	self-report	NIAAA Alcohol Use Disorder and Associated Disabilities Interview Schedule – DSM-IV Version (AUDADIS-IV)	Alcohol Dependence Determined by DSM-IV Criteria
Fergusson 1996	New Zealand	Prospective Cohort	1993-1995	Both	18-18	N/A	1,019	Lifetime	Self-reported	Study-Specific Questionnaire	0-16	Any	Alcohol Abuse or Dependence; Substance Abuse or Dependence Conduct Disorder	self-report	Composite International Diagnostic Interview (CIDI); Self-Report Delinquency Instrument (SRDI)	Diagnosis or Alcohol or Substance Abuse or Dependence; Conduct Disorder Determined by DSM-IV Criteria
Fergusson 2008	New Zealand	Prospective Cohort	1977-2022	Both	16-25	N/A	1,001	Lifetime	Self-reported	Study-Specific Questionnaire	0-16	Any	Anxiety Disorders; Depressive disorders	self-report	Composite International Diagnostic Interview (CIDI)	Anxiety Disorders and MDD Between Ages 16-25 Determined by DSM-IV criteria

Table S8: Summary characteristic of studies analyzed for childhood sexual abuse and 15 identified health outcomes.

Study	Location Name	Study Design	Years	Sex	Age Range	Mean Age (SD)	Total Sample Size	Exposure Recall Type	Exposure Assessment Method	Exposure Assessment Instrument	Age of Exposure	Perpetrator	Outcome	Outcome Assessment Method	Outcome Assessment Instrument	Outcome Definition
Fortin-Langelier 2019	Canada	Case-Control	2001-2010	Female	N/A	11.42(4.21)	1,322	Lifetime	Administrative records	Administrative Records	1-17	Any	Maternal Abortion and Miscarriage	administrative medical records or disease registries	Administrative Records	Pregnancy loss determined via administrative records
Fujiwara 2011	Japan	Case-Control	2002-2004	Both	N/A	50.8(0.6)	1,722	Lifetime	Self-reported	National Comorbidity Survey Replication (NCS-R)	0-18	Any	Anxiety Disorders	self-report	Composite International Diagnostic Interview (CIDI)	Anxiety Disorders Determined by DSM-IV Criteria
Galloeag 2017	Brazil (Rio Grande Do Sul)	Prospective Cohort	1993-2012	Female	N/A	18	1,954	Lifetime	Self-reported	Childhood Trauma Questionnaire (CTQ)	0-15	Any	Depressive Disorders	self-report	Mini-International Neuropsychiatric Interview (MINI)	Depression Determined by Study Specific Criteria
Guiney 2022	New Zealand	Prospective Cohort	1972-2019	Both	18-45	N/A	937	Lifetime	Self-reported	Study-Specific Questionnaire	0-16	Any	Suicide And Self-Harm; Sexually Transmitted Infection (STI)	self-report; administrative medical records or disease registries; physician diagnosis; biomarker	Structured interviews about self-harm and suicide; Self-reported diagnosis of sexually transmitted disease; Enzyme immunoassay analysis of serum samples for Herpes Simplex Virus 2	Lifetime Experience of Suicide Attempt; Self-reported Past Diagnosis of STI; Seropositivity for Herpes Simplex Virus 2
Han 2022	Multiple Locations	Prospective Cohort	2006-2017	Both	40-69	54.2(7.9)	81,105	Lifetime	Self-reported	Childhood Trauma Questionnaire (CTQ)	0-18	Any	Asthma	self-report; physician diagnosis	Self-report of receiving a diagnosis of asthma by a doctor *and* a yes answer to "in the last year, have you ever had wheeze or whistling in the chest?"	Lifetime Diagnosis of Asthma with past year experience of wheezing

Table S8: Summary characteristic of studies analyzed for childhood sexual abuse and 15 identified health outcomes.

Study	Location Name	Study Design	Years	Sex	Age Range	Mean Age (SD)	Total Sample Size	Exposure Recall Type	Exposure Assessment Method	Exposure Assessment Instrument	Age of Exposure	Perpetrator	Outcome	Outcome Assessment Method	Outcome Assessment Instrument	Outcome Definition
Haydon 2011	United States of America	Prospective Cohort	1994-2008	Female; Male	N/A	15.9	4,955 (F); 3,967 (M)	Lifetime	Self-reported	Study-Specific Questionnaire	0-18	Parent Or Adult Caregiver	Sexually Transmitted Infection (STI)	self-report; biomarker	Study-Specific Questionnaire (self-report of having been told by a doctor or nurse that they had chlamydia, gonorrhea, or trichomoniasis); Biomarker (urine test-identified chlamydia, gonorrhea, or trichomoniasis)	Self-reported diagnosis of or test-identified chlamydia, gonorrhea, or trichomoniasis
Houtepen 2020	United Kingdom (City of Bristol)	Prospective Cohort	1991-2009	Both	22-22	N/A	9,959	Lifetime	Self-reported; Parental Report	ACE Study Questionnaire	0-15	Any	Depressive Disorders	self-report	Clinical Interview Schedule-Revised (CIS-R)	Depressive Disorders Determined by ICD-10 Criteria
Hovens 2015	Netherlands	Retrospective Cohort	2004-2007	Both	18-64	42.6(13.9)	1,038	Lifetime	Self-reported	Study-Specific Questionnaire	0-16	Any	Anxiety Disorders	self-report	Composite International Diagnostic Interview (CIDI)	Past 2 Years Anxiety Disorders or Depressive Disorders Determined by DSM-IV Criteria
Huang 2011	United States of America	Prospective Cohort	1995-2002	Both	18-26	21.8(0.12)	4,882	Lifetime	Self-reported	Conflicts tactics scale (CTS); Childhood Trauma Questionnaire (CTQ)	0-12	Family Member or Caregiver	Substance Abuse or Dependence	self-report	Study-Specific Questionnaire (self-reported use of illicit drugs including marijuana, cocaine, crystal methamphetamines, and others)	Lifetime Substance Use
Jaffee 2002	New Zealand	Retrospective Cohort	1972-2002	Both	N/A	26	998	Lifetime	Self-reported	Study-Specific Questionnaire	0-12	Any	Depressive Disorders	self-report	Diagnostic Interview Schedule for Children (DISC-1); Diagnostic Interview Schedule (DIS)	Major Depressive Disorder at Age >17 Determined by DSM-III Criteria

Table S8: Summary characteristic of studies analyzed for childhood sexual abuse and 15 identified health outcomes.

Study	Location Name	Study Design	Years	Sex	Age Range	Mean Age (SD)	Total Sample Size	Exposure Recall Type	Exposure Assessment Method	Exposure Assessment Instrument	Age of Exposure	Perpetrator	Outcome	Outcome Assessment Method	Outcome Assessment Instrument	Outcome Definition
Kalichman 2001	United States of America (Georgia)	Retrospective Cohort	1999-1999	Male	17-72	N/A	474	Lifetime	Self-reported	Study-Specific Questionnaire	0-16	Any	Substance Abuse or Dependence	self-report	Study-Specific Questionnaire (participants asked about use of illegal substances including marijuana, nitrite inhalants or poppers, powder and crack cocaine, and methamphetamine in previous 6 months)	Past 6 Months Illicit Drug Use
Kascakova 2022	Czechia	Retrospective Cohort	2016-2016	Both	15+	N/A	2,818	Lifetime	Self-reported	Childhood Trauma Questionnaire (CTQ)	0-18	Any	Asthma; Cardiovascular Diseases (CVD); Diabetes (Type 2)	self-report	Self-reported history of asthma, ischemic heart disease, diabetes obtained during long-term health complaint interview	Lifetime Diagnosis of Asthma, CVD, or Diabetes
Kaukinen 2005	United States of America	Retrospective Cohort	1994-1996	Female	18+	N/A	7,689	Lifetime	Self-reported	[study specific questionnaire on age at first sexual assault]	0-14	Any	Substance Abuse or Dependence	self-report	[study specific questionnaire on frequency and type of substance use]	Past Month Illicit Drug Use
Kendler 2000	United States of America (Virginia)	Retrospective Cohort	1987-1997	Female	17-55	30.1(7.6)	1411	Lifetime	Self-reported; Report by The Participants Twin	Study-Specific Questionnaire	0-16	Any Adult or Person Older Than Yourself	Alcohol Abuse or Dependence; substance Abuse or Dependence; Anxiety Disorders; Depressive Disorders; Bulimia Nervosa	self-report	Adaptation of the Structured clinical Interview for DSM-III-R criteria	Alcohol or Substance Abuse or Dependence Determined by DSM-III Criteria; GAD, MDD, Bulimia Nervosa Determined by DSM-III Criteria

Table S8: Summary characteristic of studies analyzed for childhood sexual abuse and 15 identified health outcomes.

Study	Location Name	Study Design	Years	Sex	Age Range	Mean Age (SD)	Total Sample Size	Exposure Recall Type	Exposure Assessment Method	Exposure Assessment Instrument	Age of Exposure	Perpetrator	Outcome	Outcome Assessment Method	Outcome Assessment Instrument	Outcome Definition
Kerkar 2021	United States of America (Louisiana)	Retrospective Cohort	2011-2016	Female	18-45	N/A	1,050	Lifetime	Self-reported	Study-Specific Questionnaire	0-18	Any	Maternal Abortion and Miscarriage	self-report	Study-Specific Questionnaire (participants asked the outcome of each pregnancy [miscarriage, abortion, stillbirth, livebirth, molar/ectopic] during pregnancy history interview)	Experience of Abortion/Miscarriage at First Pregnancy and Any Pregnancy
Kisely 2021	Australia	Prospective Cohort	1981-2011	Both	N/A	30	2,425	Lifetime	Self-reported; Administrative records	Administrative Records	0-18	Any	Anxiety Disorders	self-report	Composite International Diagnostic Interview (CIDI)	Past Month Anxiety Disorder or Depressive Disorder Determined by DSM-IV Criteria
Kisely 2022	Australia	Prospective Cohort	1981-2011	Both	30-30	N/A	1689	Lifetime	Self-reported; Administrative records	Child Trauma Questionnaire (CTQ); Administrative Records	0-15	Any	Suicide And Self-Harm	self-report	Study-Specific Questionnaire (participants asked if they had ever hurt themselves on purpose in any way such as by taking an overdose of pills)	Past Year Self Harm
Laporte 2001	Canada	Case-Control	1996-1996	Female	N/A	23.5(7)	68	Lifetime	Self-reported	Study-Specific Questionnaire	0-16	Family Member or Caregiver	Anorexia Nervosa	physician diagnosis	Eating Attitudes Test (EAT)	Diagnosis of Anorexia Nervosa
Mall 2020	South Africa	Case-Control	2013-2018	Both	21-54	36.1(9.13)	2,097	Lifetime	Self-reported	Childhood Trauma Questionnaire (CTQ)	0-18	Any	Schizophrenic And Psychotic Disorders	physician diagnosis	Structured Clinical Interview for Diagnosis for Axis I Disorders (SCID-I)	Schizophrenic Disorders Determined by DSM-IV Criteria
Maman 2002	United Republic Of Tanzania	Prospective Cohort	1999	Female	18-99	N/A	245	Lifetime	Self-reported	Study-Specific Questionnaire	0-12	Any	Human Immunodeficiency Virus (HIV)	biomarker	HIV serostatus confirmed by blood / biomarker test	HIV Positive Serostatus

Table S8: Summary characteristic of studies analyzed for childhood sexual abuse and 15 identified health outcomes.

Study	Location Name	Study Design	Years	Sex	Age Range	Mean Age (SD)	Total Sample Size	Exposure Recall Type	Exposure Assessment Method	Exposure Assessment Instrument	Age of Exposure	Perpetrator	Outcome	Outcome Assessment Method	Outcome Assessment Instrument	Outcome Definition
Mansueto 2022	Italy (Toscana)	Case-Control	2020-2020	Female	N/A	43.07(10.9)	132	Lifetime	Self-reported	Childhood Experience of Care and Abuse Questionnaire (CECAQ)	0-15	Any	Schizophrenic And Psychotic Disorders	administrative medical records or disease registries; physician diagnosis	Composite International Diagnostic Interview (CIDI)	Psychotic Disorders Determined by WHO Criteria
Mimiaga 2009	Multiple Locations	Prospective Cohort	N/A	Male	18-99	N/A	4,066	Lifetime	Self-reported	Study-Specific Questionnaire	0-17	Any	Human Immunodeficiency Virus (HIV)	biomarker	HIV serostatus confirmed by blood / biomarker test	HIV Positive Serostatus
Monnat 2015	United States of America	Retrospective Cohort	2009-2012	Both	N/A	43.9(12.55)	52,250	Lifetime	Self-reported	ACE Questionnaire	0-18	Any	Cardiovascular Diseases (CCD); Diabetes (Type 2)	self-report	Self-reported ever being told by a doctor, nurse, or other health care professional that respondent had diabetes, heart attack, or myocardial infarction	Lifetime Diagnosis of Heart Attack or Myocardial Infarction or Diabetes
Mullen 1996	New Zealand	Retrospective Cohort	1989-1990	Female	18-65	N/A	497	Lifetime	Self-reported	Parental Bonding Instrument (PBI); Study-Specific Questionnaire	0-16	Any	Suicide And Self-Harm	self-report	Present State Examination (PSE)	Suicide Attempt At Ages >=18
Mullen 1996	New Zealand	Retrospective Cohort	1989-1990	Female	18-65	N/A	497	Lifetime	Self-reported	Parental Bonding Instrument (PBI); Study-Specific Questionnaire	0-16	Any	Depressive Disorders	self-report	Present State Examination (PSE)	Depression at Age >=18 Determined by Study Specific Criteria

Table S8: Summary characteristic of studies analyzed for childhood sexual abuse and 15 identified health outcomes.

Study	Location Name	Study Design	Years	Sex	Age Range	Mean Age (SD)	Total Sample Size	Exposure Recall Type	Exposure Assessment Method	Exposure Assessment Instrument	Age of Exposure	Perpetrator	Outcome	Outcome Assessment Method	Outcome Assessment Instrument	Outcome Definition
Murphy 2020	Denmark	Retrospective Cohort	1996-2008	Both	N/A	24(0)	2,629	Lifetime	Self-reported; Administrative records	Study-Specific Questionnaire	0-12	Any	Schizophrenic And Psychotic Disorders	administrative medical records or disease registries	ICD-10 Diagnosis of Schizophrenia identified in Danish National Registry of Patients	Psychotic Disorders Determined by ICD-10 Criteria
Naicker 2022	South Africa (Gauteng)	Prospective Cohort	1990-2020	Female; Male	28-28	N/A	752 (F); 682 (M)	Lifetime	Self-reported	Bt30 Questionnaire	0-18	Any	Human Immunodeficiency Virus (HIV)	biomarker	HIV serostatus confirmed by blood / biomarker test	HIV Positive Serostatus
Najman 2022	Australia	Prospective Cohort	1981-2011	Both	30-30	N/A	2,474	Lifetime	Self-reported	Child Trauma Questionnaire (CTQ)	0-16	Any	Alcohol Abuse or Dependence; Substance Abuse or Dependence	self-report	Composite International Diagnostic Interview (CIDI)	Alcohol or Substance Abuse or Dependence Determined by the CIDI criteria
Nelson 2002	Australia	Retrospective Cohort	1996-2000	Both	N/A	29.9(2.5)	283	Lifetime	Self-reported	Study-Specific Questionnaire	0-18	Any	Suicide And Self-Harm; Alcohol abuse or dependence; Conduct disorder; Depressive disorders	self-report	Semi-Structured Assessment for the Genetics of Alcoholism (SSAGA)	Lifetime Experience of Suicide Attempt; Alcohol dependence, Conduct Disorder, and Major Depressive Disorder determined by DSM-IV criteria
Nelson 2006	Australia	Case-Cohort	1996-2000	Both	N/A	29.9(2.5)	6,050	Lifetime	Self-reported	Study-Specific Questionnaire	0-17	Any	Substance Abuse or Dependence	self-report	Semi-Structured Assessment for the Genetics of Alcoholism (SSAGA)	Lifetime Substance Use Determined by DSM-IV Criteria
Paraventi 2011	Brazil (Sao Paulo)	Case-Control	Not reported	Female	18+	N/A	120	Lifetime	Self-reported	Study-Specific Questionnaire	0-16	Any	Anorexia Nervosa; Bulimia Nervosa	physician diagnosis	Clinical iagnosis of Anorexia Nervosa or Bulimia Nervosa based on DSM-IV criteria during psychiatric service	Clinical diagnosis of Anorexia Nervosa or Bulimia Nervosa

Table S8: Summary characteristic of studies analyzed for childhood sexual abuse and 15 identified health outcomes.

Study	Location Name	Study Design	Years	Sex	Age Range	Mean Age (SD)	Total Sample Size	Exposure Recall Type	Exposure Assessment Method	Exposure Assessment Instrument	Age of Exposure	Perpetrator	Outcome	Outcome Assessment Method	Outcome Assessment Instrument	Outcome Definition
Rajapakse 2020	Sri Lanka	Case-Control	2018-2019	Both	18-99	N/A	686	Lifetime	Self-reported	WHO Childhood Experiences International Questionnaire (ACE-IQ)	0-18	Any	Suicide And Self-Harm	self-report; administrative medical records or disease registries	Hospital Administrative Records	Suicide Attempt Determined with Hospital Admittance
Raposo 2014	United States of America	Prospective Cohort	2001-2005	Both	65-99	N/A	7,177	Lifetime	Self-reported	Childhood Trauma Questionnaire (CTQ)	0-18	Any	Anxiety Disorders	self-report	Alcohol Use Disorder and Associated Disabilities Interview Schedule (AUDADIS)	Past Year Anxiety Disorders
Ratner 2003	Canada	Retrospective Cohort	1998-2000	Male	19-35	28.6(3.6)	351	Lifetime	Self-reported	Study-Specific Questionnaire	0-14	Any	Depressive Disorders	self-report	Center for Epidemiologic Studies Depression Scale (CES-D)	Depression Determined By CES-D Criteria

Table S8: Summary characteristic of studies analyzed for childhood sexual abuse and 15 identified health outcomes.

Study	Location Name	Study Design	Years	Sex	Age Range	Mean Age (SD)	Total Sample Size	Exposure Recall Type	Exposure Assessment Method	Exposure Assessment Instrument	Age of Exposure	Perpetrator	Outcome	Outcome Assessment Method	Outcome Assessment Instrument	Outcome Definition
Rich-Edwards 2010	United States of America	Prospective Cohort	1989-2005	Female	25-44	N/A	67,391	Lifetime	Self-reported	Study-Specific Questionnaire	0-17	Any	Diabetes (Type 2)	self-report	Study-Specific Questionnaire (participants who reported a diabetes diagnosis during mailed questionnaire were sent supplemental questionnaires to report further details on diagnostic tests, symptoms and therapy, which were then used to classify cases according to National Diabetes Data Group and American Diabetes Association guidelines)	Diagnosis of Diabetes as an Adult (>=18 Years)
Roustit 2009	France	Retrospective Cohort	2005-2005	Both	18-99	N/A	3,023	Lifetime	Self-reported	Study-Specific Questionnaire	0-18	Any	Suicide And Self-Harm; Alcohol Use or Dependence; Depressive Disorder	self-report	Study-Specific Questionnaire defining suicide as at least one act committed with the intention of taking one's life since age 18; CAGE Questionnaire; Mini-International Neuropsychiatric Interview (MINI)	Suicide Attempt at Ages >=18; Alcohol Dependence Determined by Score 2+ on CAGE Questionnaire; Depression Determined by MINI

Table S8: Summary characteristic of studies analyzed for childhood sexual abuse and 15 identified health outcomes.

Study	Location Name	Study Design	Years	Sex	Age Range	Mean Age (SD)	Total Sample Size	Exposure Recall Type	Exposure Assessment Method	Exposure Assessment Instrument	Age of Exposure	Perpetrator	Outcome	Outcome Assessment Method	Outcome Assessment Instrument	Outcome Definition
Sanci 2008	Australia	Prospective Cohort	1992-2003	Female	N/A	14.91(0.39)	999	Lifetime	Self-reported	Study-Specific Questionnaire	0-16	Adult Or Older Person	Anorexia Nervosa; Bulimia Nervosa	self-report	Branched Eating Disorder Test (BET)	Anorexia Nervosa or Bulimia Nervosa Determined by Study-Specific Criteria
Sartor 2007	United States of America (Missouri)	Prospective Cohort	1995	Female	18-29	N/A	3,536	Lifetime	Self-reported	traumatic events and early childhood experiences modules; early childhood experiences module	0-16	Any; Family Member or Caregiver	Alcohol Abuse or Dependence	self-report	Semi-Structured Assessment for the Genetics of Alcoholism (SSAGA)	Alcohol Dependence Determined by DSM-IV Criteria
Seid 2022	Australia	Prospective Cohort	1996-2016	Female	45-99	N/A	5,656	Lifetime	Self-reported	Study-Specific Questionnaire	0-18	Any	Diabetes (Type 2)	self-report; physician diagnosis	Self-reported receiving a diagnosis of diabetes by a doctor or being treated for diabetes (included type I and type II)	Diagnosis of Diabetes in the Past 3 Years
Shields 2016	Canada	Retrospective Cohort	2012-2012	Both	18+	N/A	21,878	Lifetime	Self-reported	Childhood Experiences of Violence Questionnaire (CEVQ); General Social Survey	0-16	Any	Diabetes (Type 2)	self-report	Self-reported diagnosed with diabetes by a health professional (did not differentiate between type I and type II)	Diagnosis of Diabetes as an Adult (>=18 Years)
Su 2022	Canada	Prospective Cohort	2007-2018	Both	N/A	50.6(13.8)	1,351	Lifetime	Self-reported	Childhood Trauma Questionnaire (CTQ)	0-18	Any	Depressive Disorders	self-report	Composite International Diagnostic Interview (CIDI)	Major Depressive Disorder Determined by DSM-IV and ICD-10 Criteria

Table S8: Summary characteristic of studies analyzed for childhood sexual abuse and 15 identified health outcomes.

Study	Location Name	Study Design	Years	Sex	Age Range	Mean Age (SD)	Total Sample Size	Exposure Recall Type	Exposure Assessment Method	Exposure Assessment Instrument	Age of Exposure	Perpetrator	Outcome	Outcome Assessment Method	Outcome Assessment Instrument	Outcome Definition
Sweet 2013	United States of America	Prospective Cohort	2001-2005	Female ; Male	20+	N/A	608,088	Lifetime	Self-reported	[study specific questionnaire]	0-18	Any	Substance Abuse or Dependence	self-report	National Epidemiologic Survey on Alcohol and Related Conditions (NESARC) Survey	Substance Use or Dependence Determined by NESARC Criteria
Taft 2019	Australia	Prospective Cohort	1996-2009	Female	18-36	N/A	9,021	Lifetime	Self-reported	Study-Specific Questionnaire	0-18	Any	Maternal Abortion and Miscarriage	self-report	Australian Longitudinal Study on Women's Health Study Questionnaire (asks participants how many times they have experience stillbirth, miscarriage, and abortion)	Abortion For Reason Other Than Medical Abnormality
Talmon 2021	United States of America	Prospective And Retrospective (Different Models)	1967	Both	32-49	41.2(3.54)	493	Lifetime	Self-reported; Administrative records	Administrative Records	0-12	Any	Anorexia Nervosa; Bulimia Nervosa	physician diagnosis	Diagnostic Interview Schedule (DIS)	Diagnosis of Anorexia Nervosa or Bulimia Nervosa Determined by DSM-IV Criteria
Tanaka 2015	Canada	Prospective Cohort	1983-2001	Female ; Male	21-35	N/A	969 (F); 924 (M)	Lifetime	Self-reported	Childhood Experiences of Violence Questionnaire (CEVQ) Short Form	0-16	Any	Substance Abuse or Dependence	self-report	Alcohol Use Disorders Identification Test (AUDIT)	Past Month Illicit Drug Use
Tenhave 2019	Netherlands	Prospective Cohort	1996-2009	Both	18-64	N/A	9,304	Lifetime	Self-reported	Study-Specific Questionnaire	0-16	Any	Anxiety Disorders	self-report	Composite International Diagnostic Interview (CIDI)	Anxiety Disorders Determined by DSM-IV Criteria
Thomas 2008	Multiple Locations	Prospective Cohort	1958-2003	Both	45-45	N/A	9,310	Lifetime	Self-reported	Study-Specific Questionnaire	0-16	Family Member or Caregiver	Diabetes (Type 2)	physician diagnosis; biomarker	Biomarker	HbA1c ≥6.5%

Table S8: Summary characteristic of studies analyzed for childhood sexual abuse and 15 identified health outcomes.

Study	Location Name	Study Design	Years	Sex	Age Range	Mean Age (SD)	Total Sample Size	Exposure Recall Type	Exposure Assessment Method	Exposure Assessment Instrument	Age of Exposure	Perpetrator	Outcome	Outcome Assessment Method	Outcome Assessment Instrument	Outcome Definition
Thompson 2019	United States Of America	Prospective Cohort	1995-2008	Both	N/A	15.03(0.11)	9,421	Lifetime	Self-reported	Study-Specific Questionnaire	0-18	Parent Or Adult Caregiver	Suicide And Self-Harm	self-report	Study-Specific Questionnaire (“During the past 12 months, how many times did you actually attempt suicide?”)	Past Year Experience of one or more Suicide Attempts
Tonmyr 2017	Canada	Retrospective Cohort	2004-2005	Both	18-76	N/A	14,063	Lifetime	Self-reported	Study-Specific Questionnaire	0-16	Any	Substance Abuse or Dependence	self-report	Composite International Diagnostic Interview (CIDI)	Past Year Illicit Drug Use
van Roode 2009	New Zealand	Prospective Cohort	1973-2009	Female	18-32	N/A	465	Lifetime	Self-reported	Study-Specific Questionnaire	0-16	Any	Maternal Abortion and Miscarriage	self-report; biomarker	Study-Specific Questionnaire with questions on sexual and reproductive health, based on British National Study of Sexual Attitudes and Lifestyles (asked number of abortions)	Lifetime Experience of Abortion/Miscarriage
Widom 1999	United States of America	Prospective Cohort	1989-1995	N/A	N/A	28.72(3.84)	1,196	Lifetime	Administrative records	Administrative Records	0-11	Any	Posttraumatic Stress Disorder (PTSD)	self-report	Diagnostic Interview Schedule (DIS)	Past Year PTSD Determined by DSM-III Criteria
Widom 2007	United States of America	Prospective Cohort	1989-1995	Both	N/A	28.7(3.8)	1,292	Lifetime	Administrative records	Administrative Records	0-12	Anyone Or Not Specified	Depressive Disorders	self-report; interview	Diagnostic Interview Schedule (DIS)	Lifetime and Past Year Major Depressive Disorder Determined by DSM-IV Criteria
Widom 2012	United States of America	Prospective Cohort	1967-2005	Both	32-49	N/A	807	Lifetime	Administrative records	Administrative Records	0-12	Any	Human Immunodeficiency Virus (HIV)	biomarker	HIV serostatus confirmed by blood / biomarker test	HIV Positive Serostatus

Table S8: Summary characteristic of studies analyzed for childhood sexual abuse and 15 identified health outcomes.

Study	Location Name	Study Design	Years	Sex	Age Range	Mean Age (SD)	Total Sample Size	Exposure Recall Type	Exposure Assessment Method	Exposure Assessment Instrument	Age of Exposure	Perpetrator	Outcome	Outcome Assessment Method	Outcome Assessment Instrument	Outcome Definition
Wilson 2009	United States of America	Prospective Cohort	1967-2004	Both	32-49	N/A	670	Lifetime	Administrative records	Administrative Records	0-11	N/A	Sexually Transmitted Infection (STI)	self-report	Self-reported having been told by doctor or other health care professional that they had genital herpes, syphilis, genital warts or human papillomavirus, gonorrhea, or chlamydia	Self-reported history of diagnosis with an STI
Wise 2001	United States of America (Massachusetts)	Case-Control	1999-2001	Female	36-45	N/A	366	Lifetime	Self-reported	Conflict Tactics Scale (CTS)	0-18	Any	Depressive Disorders	self-report; physician diagnosis	Structured Clinical Interview For DSM-III-R (SCID)	Lifetime and Past Year Major Depressive Disorder Determined by SCID Criteria
Wyatt 2002	United States of America (California)	Case-Control	1994-2005	Female	18-99	N/A	457	Lifetime	Self-reported	Study-Specific Questionnaire	0-18	Any	Human Immunodeficiency Virus (HIV)	biomarker	HIV serostatus confirmed by blood / biomarker test	HIV Positive Serostatus
Xavier Hall 2021	United States of America (Illinois)	Retrospective Cohort	2015-2019	Both	16-29	N/A	1,035	Lifetime	Self-reported	Study-Specific Questionnaire	0-13	Any	Human Immunodeficiency Virus (HIV); Suicide and Self-Harm; Sexually Transmitted Infection (STI)	Self-report; biomarker	HIV serostatus confirmed by blood / biomarker test; Composite International Diagnostic Interview (CIDI)	HIV Positive Serostatus; Past 6 Months Experience of Suicide Attempt STI Status Determined by Clinical Testing

Table S8: Summary characteristic of studies analyzed for childhood sexual abuse and 15 identified health outcomes.

Study	Location Name	Study Design	Years	Sex	Age Range	Mean Age (SD)	Total Sample Size	Exposure Recall Type	Exposure Assessment Method	Exposure Assessment Instrument	Age of Exposure	Perpetrator	Outcome	Outcome Assessment Method	Outcome Assessment Instrument	Outcome Definition
Xiao 2022	China (Yunnan)	Case-Control	2018-2021	Both	N/A	13.47(1.71)	1,134	Lifetime	Self-reported	Childhood Trauma Questionnaire (CTQ)	0-15	Any	Depressive Disorders	physician diagnosis	Clinical diagnosis of a depressive disorder by professional pediatric psychiatrists following DSM-V and using Kiddie Schedule for Affective Disorders and Schizophrenia for School-age Children-Present and Lifetime version (K-SADS-PL)	Depression Determined by DSM-V criteria
Zinzow 2012	United States of America	Retrospective Cohort	2006-2006	Female	18-76	46.58(17.87)	3,001	Lifetime	Self-reported	Study-Specific Questionnaire	0-14	Any	Posttraumatic Stress Disorder (PTSD); Drug use disorders, Major depressive disorder	self-report	National Women's Study PTSD and Major Depressive Episode modules – structured interviews based on DSM-IV criteria	PTSD, MDD, drug abuse determined by structured interviews based upon DSM-IV Criteria

Section 1.5: Data selection and sensitivity analyses for childhood sexual abuse

Childhood sexual abuse and alcohol use disorders

For CSA-alcohol use disorders, we extracted a total of 16 studies reporting on the association. Among these studies, two reported on the same association using the Australian Twin Register younger cohort. We retained Nelson 2002 for measuring AUDs among the entire sample rather than just among users of alcohol, as this approach was most consistent with our other input studies. Two studies also published on the same association using the Missouri Adolescent Female Twin Study; among these we retained Sartor 2007 because it measured CSA in a manner most consistent with our other input dataset while the other study focused on the difference between behavior vs. checklist-defined CSA and measured lifetime alcohol use disorder rather than the development of a disorder. Three studies reported on this association using the Mater Hospital – University of Queensland Study of Pregnancy; we retained estimates from Najman 2022 because this publication used self-reported CSA exposure ascertainment rather than agency records with the longest follow-up (30 years) and used a case definition matching AUDs. Lastly, four studies reported on this association using the national epidemiologic survey on alcohol and related conditions dataset. We retained estimates from Fenton 2013 for reporting the exposure/outcome case definitions most consistent with our study's definitions and among the widest sample (other studies subset to female current drinkers only; those with data on sexuality only; or used alternate outcome definition measuring abuse and/or dependence).

These choices resulted in 10 remaining unique studies, among which four used an alternate case definition measuring alcohol abuse and/or dependence. The GBD reference definition for alcohol use disorders is alcohol dependence measured via DSM criteria. Alcohol abuse is an accepted alternate case definition used within the GBD, and the diagnostic criteria for substance abuse is similar but less extensive than for dependence. The principal difference between them is that the criteria for substance abuse focus on social and situational consequences of use and does not mention withdrawal or tolerance. Thus, in addition to a sensitivity analysis where we tested the impact of trimming 10% of outliers (Table S9), we tested restricting our input dataset to only those studies measuring alcohol dependence specifically. The results of this analysis were consistent with our main result, with a slightly increased ROS (0.195).

Table S9. Sensitivity analyses for childhood sexual abuse and alcohol use disorders.

Analysis	RR and 95%UI	ROS	Pub. Bias	Gamma	No. of studies (no. observations)	Selected bias covariates
**Main**	1.8 (1.39-2.33)	0.186	0	3.08E-08	10 (15)	Adjusted for confounding beyond age and sex; Study sample not geographically representative
**0% trimming**	1.92 (0.56-6.56)	-0.191	0	0.168	10 (15)	Adjusted for confounding beyond age and sex; Study sample not

						geographically representative
**Exclude studies using outcome definitions including alcohol abuse**	1.85 (1.42-2.41)	0.195	0	1.74E-06	6 (7)	--

Childhood sexual abuse and self-harm

We extracted data from a total of 19 studies reporting on the risk of self-harm (suicide attempt) given exposure to CSA. Among these input studies, two authors reported results using National Longitudinal Study of Adolescent to Adult Health dataset. We retained estimates from Thompson 2019 as the reported effect sizes were adjusted for confounding compared to the bivariate (unadjusted) associations reported by the other authors. Additionally, two authors reported results using the Children in the Community Cohort Study and we retained reported estimates from Brown 1999 as they incorporated both CPS substantiations and retrospective self-reports to create their exposed group while the other study only used substantiated CSA allegations to ascertain exposure.

We undertook two sensitivity analyses. For the first, we tested the impact of trimming outliers on our results. Without 10% trimming, estimated between-study heterogeneity was higher ($\gamma = 0.126$) and the ROS decreased (-0.0421). Study designs at risk of reverse causation (i.e., case-controls) were detected as a potential source of bias by our covariate selection algorithm. Consequently, for our second sensitivity analysis, we excluded case-controls and found an ROS of 0.244 (Table S10).

Table S10. Sensitivity analyses for childhood sexual abuse and self-harm.

Analysis	RR and 95%UI	ROS	Pub. Bias	Gamma	No. of studies (no. observations)	Selected bias covariates
**Main**	1.98 (1.25-3.12)	0.15	0	0.0148	16 (19)	Study at risk of reverse causation (case-control)
**0% trimming**	2.23 (0.776-6.41)	-0.0421	0	0.126	16 (19)	Study at risk of reverse causation (case-control)
**Exclude case-control designs and ever/lifetime suicide attempt case definitions**	2.07 (1.56-2.77)	0.244	0	0.00211	14 (17)	Effect size uncontrolled for confounding

Childhood sexual abuse and major depressive disorder

We extracted data from a total of 45 studies reporting on the risk of depressive disorders given exposure to CSA. Among these input studies, two authors reported results using the Children in the Community Cohort Study and we retained reported estimates from Brown 1999 as they incorporated both CPS substantiations and retrospective self-reports to create their exposed group while the other authors only used substantiated CSA allegations to ascertain exposure. Similarly, four authors reported on this association using the Mater Hospital – University of Queensland Study of Pregnancy. We retained estimates from Kisely 2021 because this study used self-reported CSA exposure and assessed current depression, rather than depression over the lifetime, providing the best assurance of temporality between CSA and adulthood outcome. Lastly, two studies reported estimates from the Netherlands Study of Depression and Anxiety; we retained estimates from Hovens 2015 for using a prospective cohort design rather than a retrospective cohort design.

We additionally excluded one originally extracted study for using an unaccepted symptom scale to measure depression (CIDI-SF scale). After making these data selections, we confirmed all studies used self-reported exposure to CSA and used diagnostic scales and/or symptom scales to measure depressive disorders among the sample (i.e., no outcomes were ascertained via health system records or self-reported physician diagnoses).

We undertook two sensitivity analyses for CSA-MDD. First, we tested the impact of trimming. Without 10% trimming, the ROS slightly decreased but association remained two-star (Table S11). Next, we excluded case-control study designs (cohort studies only); the result was consistent with our main result (Table S11).

Four studies reported sex-stratified effect sizes. Across these studies, sex-specific effects were not observed to significantly differ among men compared to women; however, there were several studies which reported results for samples made up entirely of women. This potential source of compositional bias was accounted for by incorporating a study-level covariate indicating whether a sample represented a single gender population (i.e., women only or men only). This covariate was not detected as significant by our selection algorithm, suggesting that the inclusion of women-only samples was not a source of substantial bias in our main result.

Table S11. Sensitivity analyses for childhood sexual abuse and major depressive disorder.

Analysis	RR and 95%UI	ROS	Pub. Bias	Gamma	No. of studies (no. observations)	Selected bias covariates
**Main**	1.66 (1.13-2.44)	0.0906	0	0.0137	26 (32)	--
**0% trimming**	1.89 (0.89-4.01)	0.00206	0	0.0726	26 (32)	Women only samples; exposure defined as CSA before an age less than 15

**Exclude case-control designs**	1.68 (1.16-2.42)	0.104	0	0.0116	20 (25)	--

Childhood sexual abuse and anxiety disorders

We extracted data from a total of 20 studies reporting on the risk of anxiety disorders given exposure to CSA. Among these input studies, four authors reported on this association using the Mater Hospital – University of Queensland Study of Pregnancy. We retained estimates from Kisely 2021 because this study used self-reported CSA exposure and assessed current depression, rather than depression over the lifetime, providing the best assurance of temporality between CSA and adulthood outcome. Lastly, two studies reported estimates from the Netherlands Study of Depression and Anxiety; we retained estimates from Hovens 2015 for using a prospective cohort design rather than a retrospective cohort design.

After making these data selections (n = 12 studies), 11 studies used self-reported exposure to CSA and one used administratively ascertained exposure (i.e., child protection agency records). All studies used diagnostic scales and/or symptom scales to measure depressive disorders among the sample (i.e., no outcomes were ascertained via health system records or self-reported physician diagnoses).

We undertook four sensitivity analyses for CSA-anxiety disorders. First, we tested the impact of trimming. Without 10% trimming, estimated between-study heterogeneity increased and the ROS became negative (Table S12). Next, we excluded case-control study designs (cohort studies only); the result was consistent with our main result (Table S12). Thirdly, we excluded the single study using administrative sources to ascertain CSA exposure. When doing so, the ROS was slightly lower (0.0682). Finally, we excluded four studies measuring specific anxiety disorders rather than any anxiety disorder in aggregate and found an ROS of 0.0638.

Table S12. Sensitivity analyses for childhood sexual abuse and anxiety disorders.

Analysis	RR and 95%UI	ROS	Pub. Bias	Gamma	No. of studies (no. observations)	Selected bias covariates
**Main**	1.44 (1.13-1.85)	0.0794	0	4.94E-10	12 (13)	Component outcome definition; CSA measured before an age less than 15 (i.e. 11-14)
**0% trimming**	1.75 (0.656-4.66)	-0.132	0	0.101	12 (13)	Component outcome definition; CSA measured before an age less than 15 (i.e. 11-14)
**Exclude case-control designs**	1.44 (1.13-1.85)	0.0796	0	1.26E-08	11 (12)	CSA measured before an age less than 15; CSA measured before an age greater than 15

**Exclude studies using administrative sources to ascertain exposure**	1.41 (1.1-1.81)	0.0682	0	1.74E-09	11 (12)	Women only sample; selection bias risk
**Exclude studies using component outcome (e.g., PTSD only)**	1.4 (1.09-1.8)	0.0638	0	1.12E-06	8 (9)	--

Childhood sexual abuse and drug use disorders

We extracted data from a total of 30 studies reporting on the association between CSA and drug use disorders. Among the originally identified and accepted studies, several authors reported on this association using the Mater-University of Queensland Study of Pregnancy (MUSP) cohort. From these studies, we chose to use the estimates from Najman 2022 because this analysis used self-reported CSA to create the exposed group and measured any drug use disorder rather than a specific type of substance use disorder (eg, amphetamine use disorder, cannabis use disorder). This definition was more consistent with the remainder of our input studies. In addition, several authors reported on the association between CSA and drug use outcomes using the National Epidemiological Survey of Alcohol and Related Conditions (NESARC); we retained the estimates from Sweet 2013 because they measured drug use disorders rather than illicit drug use (without a diagnosis of dependence). Lastly, three authors measured drug use outcomes using the National Longitudinal Study of Adolescent to Adult Health – we retained estimates from Huang 2011 for including all illicit drug use rather than measuring specific substances (other authors measured prescription opioid misuse; cocaine use, marijuana use). These selections resulted in 16 studies used for modeling.

Across the identified studies, there was insufficient evidence to investigate the relationship between CSA and specific drug use disorders (e.g., cannabis use disorder, amphetamine use disorder, opioid use disorder, etc.). In the future as more data becomes available, we plan to undertake analyses illuminating the specific relationships between CSA and each of these outcomes. For our analyses, we instead investigated the association between CSA and any drug use disorder. We accepted author definitions measuring presence of any drug use disorders according to DSM criteria (reference), illicit drug use (accepted alternate), and measuring presence of specific drug use disorders (accepted alternate). Accepted alternate definitions were marked with study-level bias covariates. In our main analyses, the bias covariate detection algorithm suggested that measuring specific drug use disorders (Duncan 2008, cannabis use disorders; Conroy 2009, opioid use disorders) may be a significant source of difference among included studies. In addition to our standard sensitivity analysis run without trimming, we therefore also undertook sensitivity analyses in which we subset the data to: (1) studies measuring aggregate drug use/use disorders only; (2) studies measuring use disorders only (i.e., excluding those measuring use); and (3) studies measuring aggregate drug use disorders only (i.e., combining the two exclusion reasons from analyses 1-2). Across all analyses, our results suggest a significant degree of between study heterogeneity (range of γ = 0.123 to 0.258; Table S13). This result is likely driven by the heterogenous nature of studies and/or outcome definitions included in the input data set; greater

consistency in outcome measurement can improve our ability to understand the relationship between CSA and drug use disorders moving forward.

Table S13. Sensitivity analyses for childhood sexual abuse and drug use disorders.

Analysis	RR and 95%UI	ROS	Pub. Bias	Gamma	No. of studies (no. observations)	Selected bias covariates
**Main**	1.95 (0.709-5.38)	-0.0908	0	0.123	16 (34)	Sample represents a subpopulation, effect size not controlled for age and sex plus additional confounders, study measured specific use disorder
**0% trimming**	2.06 (0.507-8.34)	-0.227	0	0.258	16 (34)	Sample represents a subpopulation, effect size not controlled for age and sex plus additional confounders, study measured specific use disorder
**Exclude studies measuring specific drug use disorders**	2.36 (0.638-8.74)	-0.12	0	0.196	13 (26)	Study measuring use without disorder criteria
**Exclude studies measuring drug use without disorder criteria**	2.55 (0.612-10.7)	-0.131	0	0.222	10 (22)	Study measuring specific drug use disorder
**Exclude studies measuring either specific use disorder or drug use without disorder criteria**	2.96 (0.675-13)	-0.078	0	0.213	8 (19)	--

Section 1.6: Supplementary results for childhood sexual abuse selected outcomes

Type 2 diabetes mellitus

We extracted 13 observations from seven studies (seven cohorts) reporting on the association between CSA and type 2 diabetes mellitus across four locations (Supplementary Table 8). Based on our conservative BPRF analyses, we estimated an ROS of -0.01, indicating weak evidence of an association.

In a sensitivity analysis run without 10% trimming, the ROS was 0.0007, corresponding to a two-star association under the BPRF framework.

HIV/AIDS

We extracted nine observations from seven studies (two case-controls and five cohorts) reporting on the association between CSA and HIV/AIDS across four locations (Supplementary Table 8). Based on our conservative BPRF analyses, we estimated an ROS of -0.04, indicating weak evidence of an association. There were fewer than 10 observations in our main analyses, thus we did not trim any outliers in this model.

Sexually transmitted infections excluding HIV

We extracted 11 observations from four studies (four cohorts) reporting on the association between CSA and sexually transmitted infections excluding HIV across two locations (Supplementary Table 8). Based on our conservative BPRF analyses, we estimated an ROS of -0.08, indicating weak evidence of an association. In a sensitivity analysis run without trimming 10% of outliers, the ROS was -0.0765.

Maternal abortion and miscarriage

We extracted eight observations from six studies (one case-control and five cohorts) reporting on the association between CSA and maternal abortion and miscarriage across five locations (Supplementary Table 8). Based on our conservative BPRF analyses, we estimated an ROS of -0.09, indicating weak evidence of an association. There were fewer than 10 observations in our main analyses, thus we did not trim any outliers in this model.

Conduct disorder

We extracted five observations from three studies (three cohorts) reporting on the association between CSA and conduct disorder across two locations (Supplementary Table 8). Based on our conservative BPRF analyses, we estimated an ROS of -0.23. There were fewer than 10 observations in our main analyses, thus we did not trim any outliers in this model.

Bulimia nervosa

We extracted seven observations from five studies (one case-control and four cohorts) reporting on the association between CSA and bulimia nervosa across four locations (Supplementary Table 8). Based on our conservative BPRF analyses, we estimated an ROS of -0.33, indicating weak evidence of an association. There were fewer than 10 observations in our main analyses, thus we did not trim any outliers in this model.

Schizophrenia

We extracted five observations from five studies (four case-control and one cohort) reporting on the association between CSA and schizophrenia across five locations (Supplementary Table 8). Based on our conservative BPRF analyses, we estimated an ROS of -0.46, indicating weak evidence of an association. There were fewer than 10 observations in our main analyses, thus we did not trim any outliers in this model.

Section 1.7: Descriptive results for additional health-related outcomes studied in association with childhood sexual abuse exposure.

High body mass index

We extracted 15 studies measuring an association between CSA and adult-onset BMI. Across these studies, three authors reported on this association using the National Longitudinal Study of Adolescent to Adult Health. We retained estimates from Fuemmeler 2009 as the exposure and outcome definitions most clearly aligned with indicators in the GBD (sexual abuse compared to no sexual abuse; overweight and overweight/obese compared to non-overweight). We also found that two authors reported on the CSA and high body mass index association using the Mater Hospital – University of Queensland Study of Pregnancy. We retained estimates from Kisely 2022 as they reported exposure and outcome definitions mostly closely aligned with our case definitions and followed participants for a longer period (30 vs. 21 years in alternate study). These selections resulted in 12 studies, across which outcome definitions corresponding to high BMI varied. Most studies reported on measure of obesity, defined as body mass index greater than or equal to 30 kg/m^{2,} while others reported on overweight, defined as body mass index 25 – 29 kg/m^2. Extended data figure 9 shows the distribution of effect sizes, with color indicating which outcome definition the study used. In general, effect sizes tend to demonstrate a harmful effect of CSA on adult-onset obesity (Extended data figure 9); however, there is substantial heterogeneity in strength and significance of these estimates.

Smoking

We extracted 10 studies which measured the risk of smoking in association with exposure to CSA. Three studies used the Mater-University of Queensland Study of Pregnancy, and we retained Najman 2022 as this study used the longest follow-up and self-reported exposure to CSA. In addition, two studies used the Nurses Health Study II. We retained estimates from Jun 2008 as the analysis approach was most consistent with other included studies (the alternate publication investigated the mediating effect of CSA on sexual orientation disparities in tobacco use). These selections resulted in seven studies. Case definitions for smoking varied across studies (Table S14). Four of the seven of the extracted and accepted studies showed a significantly harmful relationship between CSA and smoking (Extended data figure 9).

Table S14: Case definitions for smoking by study.

Study	Outcome definition
**Valerio 2022**	Current smokers are those who used one or more cigarettes a day for at least one week in the last 30 days.
**Najman 2022**	Respondents self-reported current cigarette smoking at the 30-year follow-up.
**Kalichman 2001**	self-reported tobacco use in the past 6 months.
**Nichols 2004**	Smoking status at study enrolment: participants who indicated on the study of moods and cycles baseline questionnaire that they had smoked more than 100 cigarettes in their lifetime were classified as smokers.
**Jun 2008**	Self-reported smoking between ages 15 and 19 years.
**Anda 1999**	Current smoking defined as smoking at the time of the survey.
**Tanaka 2015**	Self-reported daily cigarette smoking.

High systolic blood pressure

We identified five studies reporting on the association between CSA and a measure of high systolic blood pressure. Two studies reported this association using the same underlying cohort (National Longitudinal Study of Adolescent to Adult Health, Wave IV), and we chose to retain the study reporting a combined sex effect size (Gooding 2014) as this was most comparable with the other included studies. This selection resulted in four studies. The four included studies did not show consistent evidence of a significantly harmful effect (Extended data figure 9).

Section 2: Data input details for each risk-outcome pair

For intimate partner violence included studies, we report the observations used for modeling by outcome in Table S15. The log transformed effect size and standard error are reported. Where relevant, the column "effect size details" describes differences between multiple included observations from a single study and corresponding to the same violence type. For non-mutually exclusive data points, the factor by which we adjusted the standard error is reported in the column, "SE adjustment factor."

For childhood sexual abuse included studies, we report the observations used for modeling by outcome in Table S16. The log transformed effect size and standard error are reported. Where relevant, the column "effect size details" describes differences between multiple included observations from a single study and corresponding to the same group of participants. Across included studies, the most common reason for multiple observations were studies reporting effect sizes by frequency and/or severity of exposure. For non-mutually exclusive data points, the factor by which we adjusted the standard error is reported in the column, "SE adjustment factor."

Table S15. Summary results of included intimate partner violence studies.

Table S15. Summary results of included intimate partner violence studies

Author	Health outcome	IPV type	Effect size details	SE adjustment factor	Log effect size	Standard error of the log effect size
Ahmadabadi 2020	Anxiety disorders	Severe combined (physical and sexual)		N/A	0.10	0.52
Ahmadabadi 2020	Anxiety disorders	Physical abuse		N/A	0.41	0.25
Brown 2020	Anxiety disorders	physical; sexual; psychological		N/A	1.22	0.28
Ehrensaft 2006	Anxiety disorders	physical	Generalized Anxiety Disorder	2	0.99	0.55
Ehrensaft 2006	Anxiety disorders	physical	Posttraumatic Stress Disorder	2	1.86	0.53
Makaroun 2020	Anxiety disorders	physical; sexual	Age strata: 60-99	N/A	0.47	0.61
Makaroun 2020	Anxiety disorders	physical; sexual	Age strata: 45-59	N/A	1.34	0.26
Suglia 2011	Anxiety disorders	physical; sexual		N/A	0.67	0.34
Delong 2019	HIV/AIDS	physical; sexual		N/A	0.34	0.18
Deyessa 2018	HIV/AIDS	physical		N/A	0.28	0.36
Deyessa 2018	HIV/AIDS	sexual		N/A	0.39	0.40
Fonck 2005	HIV/AIDS	physical		N/A	0.59	0.24
Jewkes 2010	HIV/AIDS	physical; sexual		N/A	0.41	0.19
Kouyoumdjian 2013	HIV/AIDS	sexual		N/A	0.46	0.18

Table S15. Summary results of included intimate partner violence studies

Author	Health outcome	IPV type	Effect size details	SE adjustment factor	Log effect size	Standard error of the log effect size
Kouyoumdjian 2013	HIV/AIDS	physical		N/A	0.48	0.17
Maman 2002	HIV/AIDS	physical		N/A	0.88	0.51
Maman 2002	HIV/AIDS	sexual		N/A	0.87	0.49
Ahmadabadi 2020	Major depressive disorder	Severe combined (physical and sexual)		N/A	0.64	0.47
Ahmadabadi 2020	Major depressive disorder	Physical abuse		N/A	0.26	0.27
Ali 2009	Major depressive disorder	Physical and psychological		N/A	0.72	0.33
Ali 2009	Major depressive disorder	sexual		N/A	1.11	0.51
Brown 2020	Major depressive disorder	physical; sexual; psychological		N/A	1.06	0.22
Chowdhary 2008	Major depressive disorder	physical		N/A	-0.13	0.88
Chowdhary 2008	Major depressive disorder	sexual		N/A	0.11	1.45
Ehrensaft 2006	Major depressive disorder	physical		N/A	0.90	0.39
Han 2019	Major depressive disorder	physical		N/A	0.67	0.21
Llosamartínez 2019	Major depressive disorder	physical; sexual; psychological		N/A	1.27	0.73
Makaroun 2020	Major depressive disorder	physical; sexual	Age strata: 60-99	N/A	0.83	0.59
Makaroun 2020	Major depressive disorder	physical; sexual	Age strata: 45-59	N/A	1.34	0.28

Table S15. Summary results of included intimate partner violence studies

Author	Health outcome	IPV type	Effect size details	SE adjustment factor	Log effect size	Standard error of the log effect size
Ouellet-Morin 2015	Major depressive disorder	physical; sexual; psychological		N/A	0.48	0.22
Pico-Alfonso 2006	Major depressive disorder	physical; psychological		N/A	2.51	0.74
Suglia 2011	Major depressive disorder	physical; sexual		N/A	0.09	0.29
Taft 2008	Major depressive disorder	physical; sexual		N/A	0.72	0.09
Abdollahi 2015	Maternal abortion and miscarriage	physical		N/A	0.31	0.39
Bourassa 2007	Maternal abortion and miscarriage	physical; sexual		N/A	1.41	0.36
Catak 2016	Maternal abortion and miscarriage	physical		N/A	0.69	0.27
Ibrahim 2015	Maternal abortion and miscarriage	physical		N/A	2.39	0.34
Johri 2011	Maternal abortion and miscarriage	physical; sexual		N/A	0.52	0.21
Leung 2002	Maternal abortion and miscarriage	physical; sexual		N/A	1.57	0.41
Nelson 2003	Maternal abortion and miscarriage	physical		N/A	0.12	0.11

Table S15. Summary results of included intimate partner violence studies

Author	Health outcome	IPV type	Effect size details	SE adjustment factor	Log effect size	Standard error of the log effect size
Romito 2009	Maternal abortion and miscarriage	physical; sexual	Age strata: 30-99	N/A	1.75	1.59
Romito 2009	Maternal abortion and miscarriage	physical; sexual	Age strata: <30	N/A	-0.49	1.11
Taft 2019	Maternal abortion and miscarriage	physical; sexual; psychological	Exposure recall: more than 1 year ago	N/A	0.52	0.18
Taft 2019	Maternal abortion and miscarriage	physical; sexual; psychological	Exposure recall: past year	N/A	0.85	0.25
Ackard 2007	Self-harm	physical; sexual		N/A	1.16	0.61
Chowdhary 2008	Self-harm	physical		N/A	2.08	1.09
Chowdhary 2008	Self-harm	sexual		N/A	2.39	1.22
Kaslow 2000	Self-harm	physical		N/A	-0.03	0.45
Makaroun 2020	Self-harm	physical; sexual	Age strata: 45-59	N/A	1.81	0.45
Makaroun 2020	Self-harm	physical; sexual; psychological	Age strata: 60-99	N/A	0.18	0.98

Table S16. Summary results of included childhood sexual abuse studies.

Table S16. Summary results of included childhood sexual abuse studies

Author	Health outcome	Sex	Effect size details	SE adjustment factor	Log effect size	Standard error of the log effect size
Borges 2021	Alcohol use disorders	Combined Male and Female		N/A	0.18	0.21
Cutajar 2010	Alcohol use disorders	Male		N/A	1.23	0.44
Cutajar 2010	Alcohol use disorders	Female		N/A	2.19	0.43
Dinwiddie 2000	Alcohol use disorders	Male		N/A	0.00	0.82
Dinwiddie 2000	Alcohol use disorders	Female		N/A	0.92	0.48
Fenton 2013	Alcohol use disorders	Combined Male and Female		N/A	0.58	0.07
Fergusson 1996	Alcohol use disorders	Combined Male and Female	Contact CSA excluding intercourse	N/A	1.16	0.36
Fergusson 1996	Alcohol use disorders	Combined Male and Female	CSA Involving Intercourse	N/A	0.99	0.41
Fergusson 1996	Alcohol use disorders	Combined Male and Female	Non-Contact CSA	N/A	0.64	0.50
Kendler 2000	Alcohol use disorders	Female		N/A	1.09	0.32
Najman 2022	Alcohol use disorders	Combined Male and Female	Low severity CSA	N/A	0.10	0.28
Najman 2022	Alcohol use disorders	Combined Male and Female	Moderate/Severe CSA	N/A	-0.20	0.19

Table S16. Summary results of included childhood sexual abuse studies

Author	Health outcome	Sex	Effect size details	SE adjustment factor	Log effect size	Standard error of the log effect size
Nelson 2002	Alcohol use disorders	Combined Male and Female		N/A	0.44	0.22
Roustit 2009	Alcohol use disorders	Combined Male and Female		N/A	0.63	0.44
Sartor 2007	Alcohol use disorders	Female		N/A	0.70	0.17
Laporte 2001	Anorexia nervosa	Female		N/A	0.79	0.75
Paraventi 2011	Anorexia nervosa	Female	CSA frequency: At Least One Time	N/A	1.76	0.76
Paraventi 2011	Anorexia nervosa	Female	CSA frequency: 2 Times Or More	N/A	2.67	1.16
Sanci 2008	Anorexia nervosa	Female	CSA frequency: 1 Report	N/A	0.00	1.01
Sanci 2008	Anorexia nervosa	Female	CSA frequency: ≥ 2 Reports	N/A	0.69	0.54
Talmon 2021	Anorexia nervosa	Combined Male and Female		N/A	-0.21	0.54
Cohen 2001	Anxiety disorders	Combined Male and Female		N/A	1.66	0.57
Copeland 2018	Anxiety disorders	Combined Male and Female		N/A	0.59	0.34
Dinwiddie 2000	Anxiety disorders	Female		N/A	0.41	0.65

Table S16. Summary results of included childhood sexual abuse studies

Author	Health outcome	Sex	Effect size details	SE adjustment factor	Log effect size	Standard error of the log effect size
Fergusson 2008	Anxiety disorders	Combined Male and Female		N/A	0.33	0.07
Fujiwara 2011	Anxiety disorders	Combined Male and Female		N/A	0.34	0.54
Hovens 2015	Anxiety disorders	Combined Male and Female	CSA frequency: Once or Sometimes	N/A	-0.17	0.54
Hovens 2015	Anxiety disorders	Combined Male and Female	CSA frequency: Regularly or Very Often	N/A	-0.27	0.55
Kendler 2000	Anxiety disorders	Female		N/A	0.49	0.25
Kisely 2021	Anxiety disorders	Combined Male and Female		N/A	0.48	0.19
Raposo 2014	Anxiety disorders	Combined Male and Female		N/A	0.32	0.16
Tenhave 2019	Anxiety disorders	Combined Male and Female		N/A	0.34	0.17
Widom 1999	Anxiety disorders	Combined Male and Female		N/A	0.94	0.28
Zinzow 2012	Anxiety disorders	Female		N/A	1.32	0.17
Abajobir 2017	Asthma	Combined Male and Female		N/A	0.38	0.29

Table S16. Summary results of included childhood sexual abuse studies

Author	Health outcome	Sex	Effect size details	SE adjustment factor	Log effect size	Standard error of the log effect size
Coogan 2013	Asthma	Female		N/A	0.14	0.13
Coogan 2013	Asthma	Female		N/A	0.22	0.11
Han 2022	Asthma	Combined Male and Female		N/A	0.22	0.05
Kascakova 2022	Asthma	Combined Male and Female	Slovak Sample	N/A	0.83	0.61
Kascakova 2022	Asthma	Combined Male and Female	Czech Sample	N/A	0.64	0.33
Andrews 1995	Bulimia nervosa	Female		N/A	2.62	0.88
Kendler 2000	Bulimia nervosa	Female		N/A	0.31	0.53
Paraventi 2011	Bulimia nervosa	Female	CSA frequency: At Least One Time.	N/A	-0.01	0.72
Paraventi 2011	Bulimia nervosa	Female	CSA frequency: 2 Times or More	N/A	0.62	1.06
Sanci 2008	Bulimia nervosa	Female	CSA frequency: 1 Report	N/A	1.03	0.60
Sanci 2008	Bulimia nervosa	Female	CSA frequency: ≥ 2 Reports	N/A	1.74	0.48
Talmon 2021	Bulimia nervosa	Combined Male and Female		N/A	1.81	1.17

Table S16. Summary results of included childhood sexual abuse studies

Author	Health outcome	Sex	Effect size details	SE adjustment factor	Log effect size	Standard error of the log effect size
Dinwiddie 2000	Conduct disorder	Male		N/A	0.69	0.71
Dinwiddie 2000	Conduct disorder	Female		N/A	0.22	0.67
Fergusson 1996	Conduct disorder	Combined Male and Female	Contact CSA Excluding Intercourse	N/A	1.61	0.65
Fergusson 1996	Conduct disorder	Combined Male and Female	CSA Involving Intercourse	N/A	2.48	0.60
Nelson 2002	Conduct disorder	Combined Male and Female		N/A	1.10	0.41
Duncan 2015	Type 2 diabetes mellitus	Male		N/A	1.34	0.48
Duncan 2015	Type 2 diabetes mellitus	Female		N/A	-0.34	0.41
Duncan 2015	Type 2 diabetes mellitus	Male		N/A	-0.45	0.39
Duncan 2015	Type 2 diabetes mellitus	Female		N/A	0.03	0.26
Kascakova 2022	Type 2 diabetes mellitus	Combined Male and Female	Slovak Sample	N/A	0.10	0.69
Kascakova 2022	Type 2 diabetes mellitus	Combined Male and Female	Czech Sample	N/A	0.79	0.35
Monnat 2015	Type 2 diabetes mellitus	Combined Male and Female		N/A	0.12	0.08
Rich-Edwards 2010	Type 2 diabetes mellitus	Female		N/A	0.03	0.05

Table S16. Summary results of included childhood sexual abuse studies

Author	Health outcome	Sex	Effect size details	SE adjustment factor	Log effect size	Standard error of the log effect size
Rich-Edwards 2010	Type 2 diabetes mellitus	Female		N/A	0.10	0.09
Rich-Edwards 2010	Type 2 diabetes mellitus	Female		N/A	0.25	0.08
Seid 2022	Type 2 diabetes mellitus	Female		N/A	0.15	0.13
Shields 2016	Type 2 diabetes mellitus	Combined Male and Female	Both Frequent and Severe CSA	N/A	0.69	0.23
Shields 2016	Type 2 diabetes mellitus	Combined Male and Female	Severe but Infrequent CSA	N/A	0.41	0.25
Shields 2016	Type 2 diabetes mellitus	Combined Male and Female	Non-Severe CSA at Any Frequency	N/A	0.10	0.21
Thomas 2008	Type 2 diabetes mellitus	Combined Male and Female		N/A	-0.87	0.64
Borges 2021	Drug use disorders	Combined Male and Female		N/A	-0.69	2.01
Cohen 2001	Drug use disorders	Combined Male and Female		N/A	2.01	0.54
Conroy 2009	Drug use disorders	Male		N/A	-0.36	0.20
Conroy 2009	Drug use disorders	Female		N/A	0.18	0.23
Cutajar 2010	Drug use disorders	Male		N/A	1.14	0.38
Cutajar 2010	Drug use disorders	Female		N/A	2.18	0.33

Table S16. Summary results of included childhood sexual abuse studies

Author	Health outcome	Sex	Effect size details	SE adjustment factor	Log effect size	Standard error of the log effect size
Duncan 2008	Drug use disorders	Combined Male and Female		N/A	0.77	0.19
Fergusson 1996	Drug use disorders	Combined Male and Female	Non-Contact CSA	N/A	-0.36	1.04
Fergusson 1996	Drug use disorders	Combined Male and Female	Contact CSA Excluding Intercourse	N/A	0.59	0.49
Fergusson 1996	Drug use disorders	Combined Male and Female	CSA Involving Intercourse	N/A	1.89	0.42
Huang 2011	Drug use disorders	Combined Male and Female		N/A	-0.22	0.27
Kalichman 2001	Drug use disorders	Male		N/A	0.20	0.25
Kaukinen 2005	Drug use disorders	Female		N/A	0.87	0.29
Kendler 2000	Drug use disorders	Female		N/A	0.95	0.36
Najman 2022	Drug use disorders	Combined Male and Female	Low severity CSA	N/A	0.40	0.29
Najman 2022	Drug use disorders	Combined Male and Female	Moderate/Severe CSA	N/A	0.14	0.20
Nelson 2006	Drug use disorders	Combined Male and Female		N/A	0.55	0.11

Table S16. Summary results of included childhood sexual abuse studies

Author	Health outcome	Sex	Effect size details	SE adjustment factor	Log effect size	Standard error of the log effect size
Nelson 2006	Drug use disorders	Combined Male and Female		N/A	1.06	0.29
Nelson 2006	Drug use disorders	Combined Male and Female		N/A	1.13	0.28
Nelson 2006	Drug use disorders	Combined Male and Female		N/A	1.10	0.24
Nelson 2006	Drug use disorders	Combined Male and Female		N/A	0.82	0.17
Sweet 2013	Drug use disorders	Female		N/A	1.72	0.13
Sweet 2013	Drug use disorders	Male		N/A	0.89	0.07
Sweet 2013	Drug use disorders	Male		N/A	1.02	0.10
Sweet 2013	Drug use disorders	Male		N/A	0.79	0.10
Sweet 2013	Drug use disorders	Male		N/A	0.89	0.13
Sweet 2013	Drug use disorders	Female		N/A	0.90	0.07
Sweet 2013	Drug use disorders	Female		N/A	1.47	0.07
Sweet 2013	Drug use disorders	Female		N/A	1.12	0.17
Tanaka 2015	Drug use disorders	Female		N/A	0.10	0.25
Tanaka 2015	Drug use disorders	Male		N/A	0.59	0.30
Tonmyr 2017	Drug use disorders	Male		N/A	0.47	0.30

Table S16. Summary results of included childhood sexual abuse studies

Author	Health outcome	Sex	Effect size details	SE adjustment factor	Log effect size	Standard error of the log effect size
Tonmyr 2017	Drug use disorders	Female		N/A	0.88	0.24
Zinzow 2012	Drug use disorders	Female		N/A	1.39	0.42
Deyessa 2018	HIV/AIDS	Female		N/A	0.97	0.53
Maman 2002	HIV/AIDS	Female		N/A	0.47	0.54
Mimiaga 2009	HIV/AIDS	Male		N/A	0.26	0.13
Naicker 2022	HIV/AIDS	Male		N/A	1.03	1.20
Naicker 2022	HIV/AIDS	Female		N/A	1.20	0.61
Widom 2012	HIV/AIDS	Combined Male and Female		N/A	1.96	1.67
Wyatt 2002	HIV/AIDS	Female		N/A	0.34	0.20
Xavierhall 2021	HIV/AIDS	Combined Male and Female	Forced Sexual Touching CSA	N/A	-0.11	0.30
Xavierhall 2021	HIV/AIDS	Combined Male and Female	Forced Touching and Penetrative CSA	N/A	0.18	0.29
Dong 2004	Ischemic heart disease	Combined Male and Female		N/A	0.34	0.05
Kascakova 2022	Ischemic heart disease	Combined Male and Female	Czech Sample	N/A	1.19	0.44
Kascakova 2022	Ischemic heart disease	Combined Male and Female	Slovak Sample	N/A	0.53	0.85

Table S16. Summary results of included childhood sexual abuse studies

Author	Health outcome	Sex	Effect size details	SE adjustment factor	Log effect size	Standard error of the log effect size
Monnat 2015	Ischemic heart disease	Combined Male and Female		N/A	-0.15	0.13
Andrews 1995	Major depressive disorder	Female		N/A	1.25	0.70
Bifulco 1991	Major depressive disorder	Female		N/A	1.64	0.44
Chapman 2004	Major depressive disorder	Female		N/A	0.69	0.08
Chapman 2004	Major depressive disorder	Male		N/A	0.47	0.14
Cheasty 1998	Major depressive disorder	Female		N/A	0.69	0.31
Cohen 2001	Major depressive disorder	Combined Male and Female		N/A	1.57	0.56
Comijs 2013	Major depressive disorder	Combined Male and Female		N/A	1.68	0.39
Copeland 2018	Major depressive disorder	Combined Male and Female		N/A	0.79	0.38
Dinwiddie 2000	Major depressive disorder	Female		N/A	0.36	0.35
Dinwiddie 2000	Major depressive disorder	Male		N/A	0.41	0.65
Dube 2005	Major depressive disorder	Male		N/A	0.18	0.14
Dube 2005	Major depressive disorder	Female		N/A	0.34	0.10
Ebert 2019	Major depressive disorder	Combined Male and Female		N/A	2.08	0.81

Table S16. Summary results of included childhood sexual abuse studies

Author	Health outcome	Sex	Effect size details	SE adjustment factor	Log effect size	Standard error of the log effect size
Fergusson 2008	Major depressive disorder	Combined Male and Female		N/A	0.44	0.07
Galloeag 2017	Major depressive disorder	Female		N/A	-0.36	0.44
Houtepen 2020	Major depressive disorder	Combined Male and Female		N/A	0.73	0.21
Hovens 2015	Major depressive disorder	Combined Male and Female	CSA frequency: Once or Sometimes	N/A	0.24	0.43
Hovens 2015	Major depressive disorder	Combined Male and Female	CSA frequency: Regularly or Very Often	N/A	0.56	0.36
Jaffee 2002	Major depressive disorder	Combined Male and Female		N/A	0.82	0.24
Kendler 2000	Major depressive disorder	Female		N/A	0.51	0.18
Kisely 2021	Major depressive disorder	Combined Male and Female		N/A	0.59	0.17
Mullen 1996	Major depressive disorder	Female		N/A	1.35	0.33
Nelson 2002	Major depressive disorder	Female		N/A	0.65	0.10
Nelson 2002	Major depressive disorder	Combined Male and Female		N/A	0.44	0.20
Nelson 2002	Major depressive disorder	Male		N/A	0.63	0.20

Table S16. Summary results of included childhood sexual abuse studies

Author	Health outcome	Sex	Effect size details	SE adjustment factor	Log effect size	Standard error of the log effect size
Ratner 2003	Major depressive disorder	Male		N/A	0.85	0.44
Roustit 2009	Major depressive disorder	Combined Male and Female		N/A	0.70	0.31
Su 2022	Major depressive disorder	Combined Male and Female		N/A	0.36	0.10
Widom 2007	Major depressive disorder	Combined Male and Female		N/A	0.09	0.35
Wise 2001	Major depressive disorder	Female		N/A	0.59	0.22
Xiao 2022	Major depressive disorder	Combined Male and Female		N/A	0.03	0.20
Zinzow 2012	Major depressive disorder	Female		N/A	1.19	0.19
Abajobir 2018	Maternal abortion and miscarriage	Female	Pregnancy Miscarriage	2	0.22	0.70
Abajobir 2018	Maternal abortion and miscarriage	Female	Termination Of Pregnancy	2	-0.09	0.74
Demakakos 2020	Maternal abortion and miscarriage	Female	Recurrent Miscarriage	N/A	0.74	0.33
Demakakos 2020	Maternal abortion and miscarriage	Female	Single Miscarriage	N/A	0.27	0.26
Fortin-Langelier 2019	Maternal abortion and miscarriage	Female		N/A	1.20	0.34

Table S16. Summary results of included childhood sexual abuse studies

Author	Health outcome	Sex	Effect size details	SE adjustment factor	Log effect size	Standard error of the log effect size
Kerkar 2021	Maternal abortion and miscarriage	Female		N/A	0.03	0.23
Taft 2019	Maternal abortion and miscarriage	Female		N/A	0.16	0.11
Vanroode 2009	Maternal abortion and miscarriage	Female		N/A	0.34	0.19
Chatziioannidis 2019	Schizophrenia	Combined Male and Female		N/A	2.75	1.06
Cutajar 2010	Schizophrenia	Combined Male and Female		N/A	0.96	0.26
Mall 2020	Schizophrenia	Combined Male and Female		N/A	0.20	0.10
Mansueto 2022	Schizophrenia	Female		N/A	2.32	0.85
Murphy 2020	Schizophrenia	Combined Male and Female		N/A	2.06	0.63
Banyard 2004	Self-harm	Male		N/A	0.42	0.23
Bentivegna 2022	Self-harm	Female		N/A	0.58	0.08
Bentivegna 2022	Self-harm	Male		N/A	0.77	0.14
Borges 2021	Self-harm	Combined Male and Female		N/A	0.34	0.64

Table S16. Summary results of included childhood sexual abuse studies

Author	Health outcome	Sex	Effect size details	SE adjustment factor	Log effect size	Standard error of the log effect size
Brown 1999	Self-harm	Combined Male and Female		N/A	1.82	0.73
Chen 2014	Self-harm	Female		N/A	0.41	0.12
Dinwiddie 2000	Self-harm	Female		N/A	0.85	0.69
Dube 2005	Self-harm	Male		N/A	0.74	0.19
Dube 2005	Self-harm	Female		N/A	0.79	0.10
Enns 2006	Self-harm	Combined Male and Female		N/A	-1.14	1.18
Guiney 2022	Self-harm	Combined Male and Female		N/A	0.97	0.19
Kiselydmedres 2022	Self-harm	Combined Male and Female		N/A	1.10	0.36
Mullen 1996	Self-harm	Female		N/A	2.93	0.47
Nelson 2002	Self-harm	Combined Male and Female		N/A	1.00	0.35
Rajapakse 2020	Self-harm	Combined Male and Female		N/A	0.37	0.27
Roustit 2009	Self-harm	Combined Male and Female		N/A	0.97	0.47

Table S16. Summary results of included childhood sexual abuse studies

Author	Health outcome	Sex	Effect size details	SE adjustment factor	Log effect size	Standard error of the log effect size
Thompson 2019	Self-harm	Combined Male and Female		N/A	1.03	0.27
Xavierhall 2021	Self-harm	Combined Male and Female	CSA Forced Sexual Touching	N/A	-0.11	0.54
Xavierhall 2021	Self-harm	Combined Male and Female	Forced Touching and Penetrative CSA	N/A	0.79	0.44
Guiney 2022	Sexually transmitted infections excluding HIV	Combined Male and Female		N/A	0.30	0.14
Haydon 2011	Sexually transmitted infections excluding HIV	Male	Self-Reported STD in Past 12 Months	2	-0.46	1.15
Haydon 2011	Sexually transmitted infections excluding HIV	Female	Self-Reported STD in Past 12 Months	2	0.29	0.45
Haydon 2011	Sexually transmitted infections excluding HIV	Female	Test-Identified Current STD	2	0.06	0.40
Haydon 2011	Sexually transmitted infections excluding HIV	Male	Test-Identified Current STD	2	0.26	0.67
Wilson 2009	Sexually transmitted infections excluding HIV	Combined Male and Female	Specific STD: Chlamydia	4	0.41	0.92
Wilson 2009	Sexually transmitted infections excluding HIV	Combined Male and Female	Specific STD: Genital herpes	4	0.40	1.61
Wilson 2009	Sexually transmitted infections excluding HIV	Combined Male and Female	Specific STD: Gonorrhea	4	0.52	0.89

Table S16. Summary results of included childhood sexual abuse studies

Author	Health outcome	Sex	Effect size details	SE adjustment factor	Log effect size	Standard error of the log effect size
Wilson 2009	Sexually transmitted infections excluding HIV	Combined Male and Female	Specific STD: Syphilis	4	1.45	1.34
Xavierhall 2021	Sexually transmitted infections excluding HIV	Combined Male and Female	Forced Sexual Touching CSA	N/A	0.00	0.27
Xavierhall 2021	Sexually transmitted infections excluding HIV	Combined Male and Female	Forced Touching and Penetrative CSA	N/A	0.41	0.29

Section 3: PRISMA and GATHER

Section 3.1: PRISMA

Table S17. PRISMA 2020 abstract checklist

Section and Topic	Item #	Checklist item	Reported (Yes/No)
**TITLE**			
Title	1	Identify the report as a systematic review.	Yes, briefly.
**BACKGROUND**			
Objectives	2	Provide an explicit statement of the main objective(s) or question(s) the review addresses.	Yes.
**METHODS**			
Eligibility criteria	3	Specify the inclusion and exclusion criteria for the review.	Not in abstract. Covered in detail in the main text and supplementary information.
Information sources	4	Specify the information sources (e.g. databases, registers) used to identify studies and the date when each was last searched.	Not in abstract. Covered in detail in the main text and supplementary information.
Risk of bias	5	Specify the methods used to assess risk of bias in the included studies.	Not in abstract. Covered in detail in the main text and supplementary information.
Synthesis of results	6	Specify the methods used to present and synthesise results.	Yes, briefly. More detail provided in the main text and supplementary information.
**RESULTS**			
Included studies	7	Give the total number of included studies and participants and summarise relevant characteristics of studies.	Not in abstract. Covered in detail in the main text and supplementary information.
Synthesis of results	8	Present results for main outcomes, preferably indicating the number of included studies and participants for each. If meta-analysis was done, report the summary estimate and confidence/credible interval. If comparing groups, indicate the direction of the effect (i.e. which group is favoured).	Yes, very briefly. More detail provided in the main text and supplementary information.
**DISCUSSION**			
Limitations of evidence	9	Provide a brief summary of the limitations of the evidence included in the review (e.g. study risk of bias, inconsistency and imprecision).	Not in abstract. Covered in detail in the main text and supplementary information.
Interpretation	10	Provide a general interpretation of the results and important implications.	Yes
**OTHER**			
Funding	11	Specify the primary source of funding for the review.	Not in abstract but described in the main text.
Registration	12	Provide the register name and registration number.	Not in abstract but reported in main text methods section.

Table S18. PRISMA 2020 checklist

Section and	Ite	Checklist item	Location where item is

Topic	m #		reported
**TITLE**			
Title	1	Identify the report as a systematic review.	Manuscript Title and Methods Section Headings
**ABSTRACT**			
Abstract	2	See the PRISMA 2020 for Abstracts checklist.	Abstract section in main text
**INTRODUCTION**			
Rationale	3	Describe the rationale for the review in the context of existing knowledge.	Main section in-text
Objectives	4	Provide an explicit statement of the objective(s) or question(s) the review addresses.	Main section in-text
**METHODS**			
Eligibility criteria	5	Specify the inclusion and exclusion criteria for the review and how studies were grouped for the syntheses.	"Systematic Review" section in Methods & Section 1.2: Inclusion and Exclusion Criteria in Supplementary Information
Information sources	6	Specify all databases, registers, websites, organisations, reference lists and other sources searched or consulted to identify studies. Specify the date when each source was last searched or consulted.	"Systematic Review" section in Methods & Section 1.1: Literature searches in Supplementary Information
Search strategy	7	Present the full search strategies for all databases, registers and websites, including any filters and limits used.	"Systematic Review" section in Methods & Section 1.1: Literature searches in Supplementary Information
Selection process	8	Specify the methods used to decide whether a study met the inclusion criteria of the review, including how many reviewers screened each record and each report retrieved, whether they worked independently, and if applicable, details of automation tools used in the process.	"Systematic Review" section in Methods main text
Data collection process	9	Specify the methods used to collect data from reports, including how many reviewers collected data from each report, whether they worked independently, any processes for obtaining or confirming data from study investigators, and if applicable, details of automation tools used in the process.	"Systematic Review" section in Methods main text
Data items	10a	List and define all outcomes for which data were sought. Specify whether all results that were compatible with each outcome domain in each study were sought (e.g. for all measures, time points, analyses), and if not, the methods used to decide which results to collect.	"Data selection" section in Methods main text; Supplementary Information Section 1.3: Exposure and Outcome Definitions; Table S2: Definitions of included risk factors & Table S3: Definitions of included outcomes
	10b	List and define all other variables for which data were sought (e.g. participant and intervention characteristics, funding sources). Describe any assumptions made about any missing or unclear information.	Section 1.4; Data Extraction in Supplementary Information
Study risk of bias	11	Specify the methods used to assess risk of bias in the included studies, including details of the tool(s) used, how many reviewers assessed each study and whether they worked	"Testing and adjusting for biases across study

assessment		independently, and if applicable, details of automation tools used in the process.	designs and characteristics” in Methods section; Supplementary Information Section 5.1.1: Definitions of bias covariates
Effect measures	12	Specify for each outcome the effect measure(s) (e.g. risk ratio, mean difference) used in the synthesis or presentation of results.	Section 2.2: Effect Size Data Details in Supplementary Information
Synthesis methods	13a	Describe the processes used to decide which studies were eligible for each synthesis (e.g. tabulating the study intervention characteristics and comparing against the planned groups for each synthesis (item #5)).	“Data Selection” in Methods main text
	13b	Describe any methods required to prepare the data for presentation or synthesis, such as handling of missing summary statistics, or data conversions.	Methods main text; Section 6 in Supplementary Information
	13c	Describe any methods used to tabulate or visually display results of individual studies and syntheses.	Methods main text; Sections 4-6 in Supplementary Information
	13d	Describe any methods used to synthesize results and provide a rationale for the choice(s). If meta-analysis was performed, describe the model(s), method(s) to identify the presence and extent of statistical heterogeneity, and software package(s) used.	“Estimating the burden of proof risk function” methods main text; Sections 4-6 in Supplementary Information
	13e	Describe any methods used to explore possible causes of heterogeneity among study results (e.g. subgroup analysis, meta-regression).	“Quantifying between-study heterogeneity” methods main text; Supplementary Information Section 6: Sensitivity Analyses and Supplementary Results
	13f	Describe any sensitivity analyses conducted to assess robustness of the synthesized results.	Results section in main text; Supplementary Information Section 6: Sensitivity Analyses and Supplementary Results
Reporting bias assessment	14	Describe any methods used to assess risk of bias due to missing results in a synthesis (arising from reporting biases).	“Testing and adjusting for biases across study designs and characteristics” in Methods main text; Section 5: Supplementary Methods in Supplementary Information
Certainty assessment	15	Describe any methods used to assess certainty (or confidence) in the body of evidence for an outcome.	“Estimating the burden of proof risk function” in Methods main text
**RESULTS**			
Study selection	16a	Describe the results of the search and selection process, from the number of records identified in the search to the number of studies included in the review, ideally using a flow diagram.	PRISMA flow diagram (Section 1.5 in Supplementary Information); “Data selection” in methods main text

	16b	Cite studies that might appear to meet the inclusion criteria, but which were excluded, and explain why they were excluded.	N/A
Study characteristics	17	Cite each included study and present its characteristics.	Table S6: Summary of Study Metadata and Characteristics for intimate partner violence & Table S7: Summary of Study Metadata and Characteristics for childhood sexual abuse (Section 2: Data Inputs)
Risk of bias in studies	18	Present assessments of risk of bias for each included study.	Table S10: Bias covariates for intimate partner studies & Table S11: Study Bias Characteristics for childhood sexual abuse (Section 3)
Results of individual studies	19	For all outcomes, present, for each study: (a) summary statistics for each group (where appropriate) and (b) an effect estimate and its precision (e.g. confidence/credible interval), ideally using structured tables or plots.	Section 4: The RR Estimates and their 95% Uncertainty Interval for All Risk-Outcome Pairs
Results of syntheses	20a	For each synthesis, briefly summarise the characteristics and risk of bias among contributing studies.	Section 2: Data inputs in Supplementary Information; Section 3: Study quality and bias assessment in Supplementary Information
	20b	Present results of all statistical syntheses conducted. If meta-analysis was done, present for each the summary estimate and its precision (e.g. confidence/credible interval) and measures of statistical heterogeneity. If comparing groups, describe the direction of the effect.	Results in main text; Section 4: Supplementary results in Supplementary Information
	20c	Present results of all investigations of possible causes of heterogeneity among study results.	Section 6: Sensitivity Analyses and Supplementary Results in Supplementary Information
	20d	Present results of all sensitivity analyses conducted to assess the robustness of the synthesized results.	Section 6: Sensitivity Analyses and Supplementary Results in Supplementary Information
Reporting biases	21	Present assessments of risk of bias due to missing results (arising from reporting biases) for each synthesis assessed.	Results section main text; Section 6 in Supplementary Results
Certainty of evidence	22	Present assessments of certainty (or confidence) in the body of evidence for each outcome assessed.	Results section main text
**DISCUSSION**			
Discussion	23a	Provide a general interpretation of the results in the context of other evidence.	Discussion section main text
	23b	Discuss any limitations of the evidence included in the review.	Discussion section main text
	23c	Discuss any limitations of the review processes used.	Discussion section main text
	23d	Discuss implications of the results for practice, policy, and future research.	Discussion section main

			text
**OTHER INFORMATION**			
Registration and protocol	24a	Provide registration information for the review, including register name and registration number, or state that the review was not registered.	The systematic review was registered in PROSPERO (CRD42022299831); The entirety of the Global Burden of Diseases, Injuries, and Risk Factors Study has been registered and approved through the UW IRB, as detailed in Methods section main text.
	24b	Indicate where the review protocol can be accessed, or state that a protocol was not prepared.	Review protocol publication cited in Methods section main text; review was registered in PROSPERO (CRD42022299831)
	24c	Describe and explain any amendments to information provided at registration or in the protocol.	NA
Support	25	Describe sources of financial or non-financial support for the review, and the role of the funders or sponsors in the review.	Main text "Acknowledgments"
Competing interests	26	Declare any competing interests of review authors.	Main text "Competing interests"
Availability of data, code and other materials	27	Report which of the following are publicly available and where they can be found; template data collection forms; data extracted from included studies; data used for all analyses; analytic code; any other materials used in the review.	Main text "Data availability" and "Code availability" sections; data collection form template in SI Table 5

Section 3.2: GATHER

Table S19: GATHER Checklist

Item #	Checklist item	Reported on page #
Objectives and funding		
1	Define the indicator(s), populations (including age, sex, and geographic entities), and time period(s) for which estimates were made.	Main text Methods overview and systematic review sections (p 41-43)
2	List the funding sources for the work.	Main text Acknowledgement section (Pg 12)
Data Inputs		
For all data inputs from multiple sources that are synthesized as part of the study:		
3	Describe how the data were identified and how the data were accessed.	Main text Methods overview and systematic review sections (p 41-43)
4	Specify the inclusion and exclusion criteria. Identify all ad-hoc exclusions.	Main text Methods systematic review section (p 41-43); Supplementary Information Section 4.2 (p 77)
5	Provide information on all included data sources and their main characteristics. For each data source used, report reference information or contact name/institution, population represented, data collection method,	SI Table S1 & S8 (Pg 4 and 15)

	year(s) of data collection, sex and age range, diagnostic criteria or measurement method, and sample size, as relevant.	
6	Identify and describe any categories of input data that have potentially important biases (e.g., based on characteristics listed in item 5).	SI Section 6 (Pg 94)
For data inputs that contribute to the analysis but were not synthesized as part of the study:		
7	Describe and give sources for any other data inputs.	N/A
For all data inputs:		
8	Provide all data inputs in a file format from which data can be efficiently extracted (e.g., a spreadsheet rather than a PDF), including all relevant meta-data listed in item 5. For any data inputs that cannot be shared because of ethical or legal reasons, such as third-party ownership, provide a contact name or the name of the institution that retains the right to the data.	Data inputs in excel format available on the GHDx (https://preview.healthdata.org/burden-of-proof/; username: PreviewUser; password: IHMEPreview)
Data analysis		
9	Provide a conceptual overview of the data analysis method. A diagram may be helpful.	Main text methods overview section (p 41)
10	Provide a detailed description of all steps of the analysis, including mathematical formulae. This description should cover, as relevant, data cleaning, data pre-processing, data adjustments and weighting of data sources, and mathematical or statistical model(s).	Main text methods section (p 41)
11	Describe how candidate models were evaluated and how the final model(s) were selected.	Main text methods "Model validation" section (p 47)
12	Provide the results of an evaluation of model performance, if done, as well as the results of any relevant sensitivity analysis.	Main text methods "Model validation" section (p 47); SI section 1.2 and 1.5 (p 10 and 36)
13	Describe methods for calculating uncertainty of the estimates. State which sources of uncertainty were, and were not, accounted for in the uncertainty analysis.	Main text methods "Quantifying between-study heterogeneity" (p 46)
14	State how analytic or statistical source code used to generate estimates can be accessed.	https://github.com/ihmeuw-msca/burden-of-proof/
Results and Discussion		
15	Provide published estimates in a file format from which data can be efficiently extracted.	(https://preview.healthdata.org/burden-of-proof/; username: PreviewUser; password: IHMEPreview)
16	Report a quantitative measure of the uncertainty of the estimates (e.g. uncertainty intervals).	Results section in main text (p 4-8); Table 2
17	Interpret results in light of existing evidence. If updating a previous set of estimates, describe the reasons for changes in estimates.	Main text discussion section (p 8-12)
18	Discuss limitations of the estimates. Include a discussion of any modelling assumptions or data limitations that affect interpretation of the estimates.	Main text discussion section (p 8-12)

Section 4: Data source identification and assessment

We conducted a systematic search of 7 databases (PubMed; Embase/Elsevier; Cumulative Index to Nursing and Allied Health Literature (CINAHL), PsychInfo; Global Index Medicus; Cochrane; Web of Science) to identify relevant literature published from 1 January 1970 to 31 January 2023. The date of our original search was September 30, 2021, and our search was updated to include literature published between 30 September and 31 January 2023 on February 6, 2023. The search strings applied to each database are reported in Section 4.1 and have been previously published in our review protocol. Across the seven databases searched, we found 242,397 results, of which 175,198 were duplicative, leaving 67,221 unique studies. Systematic reviews that were captured through our search were flagged for separate citation searching process (Supplementary Information Section 4.3). All sources were title/abstract and full text screened for acceptance based on the inclusion and exclusion criteria detailed in section 4.2.

Section 4.1: Literature searches

PubMed Search String

("Sex Offenses"[mh] OR "Violence"[mh:noexp] OR "Domestic Violence"[mh] OR "Gender-Based Violence"[mh] OR "Intimate Partner Violence"[mh] OR "Physical Abuse"[mh] OR "Rape"[mh] OR "Torture"[mh] OR "Workplace Violence"[mh] OR "Gun violence"[mh] OR "Battered Women"[mh] OR "Adult Survivors of Child abuse"[mh] OR "Exposure to Violence"[mh] OR "Emotional Abuse"[mh] OR "Sexual Harassment"[mh] OR "Harassment, Non-Sexual"[mh:noexp] OR "Aggression"[mh:noexp] OR "Coercion"[Mesh] OR "Dehumanization"[mh] OR "stalking"[mh] OR “adverse childhood experiences”[mh] OR violence[tiab] OR "sexual assault"[tiab] OR "sexual harassment"[tiab] OR “sexual abuse”[tiab] OR “sex abuse”[tiab] OR rape[tiab] OR “forced sex”[tiab] OR “sexual coercion”[tiab] OR “reproductive coercion”[tiab] OR “sex trafficking”[tiab] OR “sexual exploitation”[tiab] OR “forced marriage”[tiab] OR “child marriage*”[tiab] OR “early marriage*”[tiab] OR “child bride*”[tiab] OR CEFM[tiab] OR “female genital mutilation”[tiab] OR “female genital cutting”[tiab] OR “female circumcision”[tiab] OR “female genital circumcision”[tiab] OR infibulation*[tiab] OR clitoridectom*[tiab] OR clitorectom*[tiab] OR “ritual female genital surger*”[tiab] OR FGM[tiab] OR “physical abuse”[tiab] OR “psychological abuse”[tiab] OR “emotional abuse”[tiab] OR “economic abuse”[tiab] OR “financial abuse”[tiab] OR "verbal abuse"[tiab] OR maltreatment[tiab] OR “violent discipline”[tiab] OR “corporal punishment”[tiab] OR “adverse childhood experience*”[tiab] OR molestation[tiab] OR “child abuse”[tiab] OR “partner abuse”[tiab] OR "dating abuse"[tiab] OR “wife abuse”[tiab] OR “spouse abuse”[tiab] OR “domestic abuse”[tiab] OR “elder abuse”[tiab] OR “senior abuse”[tiab] OR “aged abuse”[tiab] OR victimization[tiab] OR dehumanization[tiab] OR victimisation[tiab] OR dehumanisation[tiab] OR stalking[tiab] OR cyberviolence[tiab] OR cybervictimization[tiab] OR cyberstalking[tiab])

AND

(Case-Control Studies[mh] OR Cross-Over Studies[mh] OR Cohort Studies[mh] OR Systematic Review[pt] OR Meta-Analysis[pt] OR "Twin Study"[pt] OR “systematic review”[tiab] OR “meta-analysis”[tiab] OR “cohort”[tiab] OR “cross-over”[tiab] OR “case-control”[tiab] OR “prospective”[tiab] OR “retrospective”[tiab] OR “longitudinal”[tiab] OR “follow-up”[tiab] OR “followup”[tiab])

AND

("Statistics as Topic"[mh] OR Risk[mh] OR Odds Ratio[mh] OR “risk*”[tiab] OR “odds”[tiab] OR “cross-product ratio*”[tiab] OR “hazards ratio*”[tiab] OR “hazard ratio*”[tiab] OR statistic*[tiab] OR “HR”[tiab] OR “RR”[tiab] OR “aOR”[tiab] OR relation*[tiab] OR correlat*[tiab] OR associat*[tiab] OR likel*[tiab])

AND

("1970/01/01"[PDat] : "2023/01/31"[PDat])

Embase Search String

('sexual violence'/exp OR 'forced sex'/exp OR 'violence'/de OR 'domestic violence'/exp OR 'gender based violence'/exp OR 'partner violence'/exp OR 'dating violence'/exp OR 'physical abuse'/exp OR 'physical violence'/exp OR 'torture'/exp OR 'workplace violence'/exp OR 'gun violence'/exp OR 'battered woman'/exp OR 'child abuse survivor'/exp OR 'exposure to violence'/exp OR 'emotional abuse'/exp OR 'elderly abuse'/exp OR 'sexual harassment'/exp OR 'non-sexual harassment'/de OR 'aggression'/de OR 'verbal hostility'/exp OR 'coercion'/exp OR 'intimidation'/exp OR 'dehumanization'/exp OR 'stalking'/de OR 'childhood adversity'/exp OR 'maltreatment'/exp OR 'corporal punishment'/exp OR 'victimization'/exp OR (violence OR 'sexual assault' OR 'sexual harassment' OR 'sexual abuse' OR 'sex abuse' OR rape OR 'forced sex' OR 'sexual coercion' OR 'reproductive coercion' OR 'sex trafficking' OR 'sexual exploitation' OR 'forced marriage*' OR ‘child marriage*’ OR ‘early marriage*’ OR ‘child bride*’ OR CEFM OR 'female genital mutilation' OR 'female genital cutting' OR 'female circumcision' OR 'female genital circumcision' OR infibulation* OR clitoridectom* OR clitorectom* OR ‘ritual female genital surger*’ OR FGM OR 'physical abuse' OR 'psychological abuse' OR 'emotional abuse' OR 'economic abuse' OR ‘financial abuse’ OR 'verbal abuse' OR maltreatment OR 'violent discipline' OR 'corporal punishment' OR 'adverse childhood experience*' OR molestation OR 'child abuse' OR 'partner abuse' OR 'dating abuse' OR 'wife abuse' OR 'spouse abuse' OR 'domestic abuse' OR 'elder abuse' OR 'senior abuse' OR 'aged abuse' OR victimization OR dehumanisation OR victimisation OR dehumanization OR stalking OR cyberviolence OR cybervictimization OR cyberstalking):ti,ab,kw)

AND

('case control study'/exp OR 'crossover procedure'/exp OR 'cohort analysis'/exp OR 'systematic review'/exp OR 'systematic review (topic)'/exp OR 'meta analysis'/exp OR 'meta analysis (topic)'/exp OR 'twin study'/exp OR ('systematic review' OR meta-analysis OR cohort OR cross-over OR case-control OR prospective OR retrospective OR longitudinal OR follow-up OR followup):ti,ab,kw)

AND

('statistics'/exp OR 'statistical parameters'/exp OR 'risk'/exp OR 'risk ratio'/exp OR 'correlation'/exp OR 'statistical analysis'/exp OR ('risk*' OR 'odds' OR 'cross-product ratio*' OR 'hazards ratio*' OR 'hazard ratio*' OR statistic* OR 'HR' OR 'RR' OR 'aOR' OR relation* OR correlat* OR associat* OR likel*):ti,ab,kw)

AND

[1970-2023]/py

AND

[01-01-1970]/sd NOT [31-01-2023]/sd

Cumulative Index to Nursing and Allied Health Literature (CINAHL) Search String

((MH ("Sexual Abuse+" OR "Violence" OR "Domestic Violence+" OR "Gender-Based Violence" OR "Circumcision, Female" OR "Dating Violence" OR "Torture" OR "Workplace Violence" OR "Gun Violence" OR "Battered Women" OR "Child Abuse Survivors" OR "Exposure to Violence" OR "Emotional Abuse" OR "Sexual Harassment" OR "Aggression" OR "Verbal Abuse" OR "Coercion" OR "Dehumanization" OR "Stalking" OR "Adverse Childhood Experiences" OR "Sibling Violence" OR "School Violence" OR "Student Abuse")) OR TI(violence OR "sexual assault" OR "sexual harassment" OR "sexual abuse" OR "sex abuse" OR rape OR "forced sex" OR "sexual coercion" OR "reproductive coercion" OR "sex trafficking" OR "sexual exploitation" OR "forced marriage" OR “child marriage*” OR “early marriage*” OR “child bride*” OR CEFM OR "female genital mutilation" OR "female genital cutting" OR "female circumcision" OR "female genital circumcision" OR infibulation* OR clitoridectom* OR clitorectom* OR “ritual female genital surger*” OR FGM OR "physical abuse" OR "psychological abuse" OR "emotional abuse" OR "economic abuse" OR “financial abuse” OR "verbal abuse" OR maltreatment OR "violent discipline" OR "corporal punishment" OR "adverse childhood experience*" OR molestation OR "child abuse" OR "partner abuse" OR "dating abuse" OR "wife abuse" OR "spouse abuse" OR "domestic abuse" OR "elder abuse" OR "senior abuse" OR "aged abuse" OR victimization OR dehumanization OR victimisation OR dehumanisation OR stalking OR cyberviolence OR cybervictimization OR cyberstalking) OR AB(violence OR "sexual assault" OR "sexual harassment" OR "sexual abuse" OR "sex abuse" OR rape OR "forced sex" OR "sexual coercion" OR "reproductive coercion" OR "sex trafficking" OR "sexual exploitation" OR "forced marriage" OR “child marriage*” OR “early marriage*” OR “child bride*” OR CEFM OR "female genital mutilation" OR "female genital cutting" OR "female circumcision" OR "female genital circumcision" OR infibulation* OR clitoridectom* OR clitorectom* OR “ritual female genital surger*” OR FGM OR "physical abuse" OR "psychological abuse" OR "emotional abuse" OR "economic abuse" OR “financial abuse” OR "verbal abuse" OR maltreatment OR "violent discipline" OR "corporal punishment" OR "adverse childhood experience*" OR molestation OR "child abuse" OR "partner abuse" OR "dating abuse" OR "wife abuse" OR "spouse abuse" OR "domestic abuse" OR "elder abuse" OR "senior abuse" OR "aged abuse" OR victimization OR dehumanization OR victimisation OR dehumanisation OR stalking OR cyberviolence OR cybervictimization OR cyberstalking))

AND

((MH ("Case Control Studies+" OR "Crossover Design" OR "Systematic Review" OR "Meta Analysis" OR "Prospective Studies+" OR "Retrospective Design")) OR TI("systematic review" OR meta-analysis OR cohort OR cross-over OR case-control OR prospective OR retrospective OR longitudinal OR follow-up OR followup) OR AB("systematic review" OR meta-analysis OR cohort OR cross-over OR case-control OR prospective OR retrospective OR longitudinal OR follow-up OR followup))

AND

((MH ("Statistics+" OR "Data Analysis, Statistical+")) OR TI(risk* OR odds OR "cross-product ratio*" OR "hazards ratio*" OR "hazard ratio*" OR statistic* OR "HR" OR "RR" OR "aOR" OR relation* OR correlat* OR associat* OR likel*) OR AB(risk* OR odds OR "cross-product ratio*" OR "hazards ratio*" OR "hazard ratio*" OR statistic* OR "HR" OR "RR" OR "aOR" OR relation* OR correlat* OR associat* OR likel*))

)

Limits: Publication Date: January 1970 – January 2023

PsycINFO Search String

(DE("Sexual Violence" OR "Sex Offenses" OR "Sexual Abuse" OR "Sexual Coercion" OR "Sex Trafficking" OR "Violence" OR "Domestic Violence" OR "Child Abuse" OR "Elder Abuse" OR "Circumcision" OR "Intimate Partner Violence" OR "Dating Violence" OR "Physical Abuse" OR "Physical Discipline" OR "Punishment" OR "Rape" OR "Acquaintance Rape" OR "Torture" OR "Workplace Violence" OR "Gun Violence" OR "Battered Females" OR "Exposure to Violence" OR "Emotional Abuse" OR "Sexual Harassment" OR "Aggressive Behavior" OR "Verbal Abuse" OR "Coercion" OR "Stalking" OR "Childhood Adversity" OR "School Violence" OR "Police Violence" OR "Victimization")

OR

MA("Sex Offenses" OR "Domestic Violence" OR "Gender-Based Violence" OR "Intimate Partner Violence" OR "Physical Abuse" OR "Rape" OR "Torture" OR "Workplace Violence" OR "Gun violence" OR "Battered Women" OR "Adult Survivors of Child abuse" OR "Exposure to Violence" OR "Emotional Abuse" OR "Sexual Harassment" OR "Coercion" OR "Dehumanization" OR "stalking" OR “adverse childhood experiences”)

OR

TI(violence OR "sexual assault" OR "sexual harassment" OR “sexual abuse” OR “sex abuse” OR rape OR “forced sex” OR “sexual coercion” OR “reproductive coercion” OR “sex trafficking” OR “sexual exploitation” OR “forced marriage*” OR “child marriage*” OR “early marriage*” OR “child bride*” OR CEFM OR “female genital mutilation” OR “female genital cutting” OR “female circumcision” OR “female genital circumcision” OR infibulation* OR clitoridectom* OR clitorectom* OR “ritual female genital surger*” OR FGM OR “physical abuse” OR “psychological abuse” OR “emotional abuse” OR “economic abuse” OR “financial abuse” OR "verbal abuse" OR maltreatment OR “violent discipline” OR “corporal punishment” OR “adverse childhood experience*” OR molestation OR “child abuse” OR “partner abuse” OR "dating abuse" OR “wife abuse” OR “spouse abuse” OR “domestic abuse” OR “elder abuse” OR “senior abuse” OR “aged abuse” OR victimization OR dehumanization OR victimisation OR dehumanisation OR stalking OR cyberviolence OR cybervictimization OR cyberstalking)

OR

AB(violence OR "sexual assault" OR "sexual harassment" OR “sexual abuse” OR “sex abuse” OR rape OR “forced sex” OR “sexual coercion” OR “reproductive coercion” OR “sex trafficking” OR “sexual exploitation” OR “forced marriage*” OR “child marriage*” OR “early marriage*” OR “child bride*” OR CEFM OR “female genital mutilation” OR “female genital cutting” OR “female circumcision” OR “female genital circumcision” OR infibulation* OR clitoridectom* OR clitorectom* OR “ritual female genital surger*” OR FGM OR “physical abuse” OR “psychological abuse” OR “emotional abuse” OR “economic abuse” OR “financial abuse” OR "verbal abuse" OR maltreatment OR “violent discipline” OR “corporal punishment” OR “adverse childhood experience*” OR molestation OR “child abuse” OR “partner abuse” OR "dating abuse" OR “wife abuse” OR “spouse abuse” OR “domestic abuse” OR “elder abuse” OR “senior abuse” OR “aged abuse” OR victimization OR dehumanization OR victimisation OR dehumanisation OR stalking OR cyberviolence OR cybervictimization OR cyberstalking)

OR

KW(violence OR "sexual assault" OR "sexual harassment" OR “sexual abuse” OR “sex abuse” OR rape OR “forced sex” OR “sexual coercion” OR “reproductive coercion” OR “sex trafficking” OR “sexual exploitation” OR “forced marriage*” OR “child marriage*” OR “early marriage*” OR “child bride*” OR CEFM OR “female genital mutilation” OR “female genital cutting” OR “female circumcision” OR “female genital circumcision” OR infibulation* OR clitoridectom* OR clitorectom* OR “ritual female genital surger*” OR FGM OR “physical abuse” OR “psychological abuse” OR “emotional abuse” OR “economic abuse” OR “financial abuse” OR "verbal abuse" OR maltreatment OR “violent discipline” OR “corporal punishment” OR “adverse childhood experience*” OR molestation OR “child abuse” OR “partner abuse” OR "dating abuse" OR “wife abuse” OR “spouse abuse” OR “domestic abuse” OR “elder abuse” OR “senior abuse” OR “aged abuse” OR victimization OR dehumanization OR victimisation OR dehumanisation OR stalking OR cyberviolence OR cybervictimization OR cyberstalking))

AND

(DE("Cohort Analysis" OR "Followup Studies" OR "Longitudinal Studies" OR "Retrospective Studies" OR "Prospective Studies" OR "Systematic Review" OR "Meta Analysis")

OR

MA("Case-Control Studies" OR "Cross-Over Studies" OR "Cohort Studies")

OR

TI("systematic review" OR meta-analysis OR cohort OR cross-over OR case-control OR prospective OR retrospective OR longitudinal OR follow-up OR followup)

OR

AB("systematic review" OR meta-analysis OR cohort OR cross-over OR case-control OR prospective OR retrospective OR longitudinal OR follow-up OR followup)

OR

KW("systematic review" OR meta-analysis OR cohort OR cross-over OR case-control OR prospective OR retrospective OR longitudinal OR follow-up OR followup))

AND

(DE("Statistics" OR "Statistical Analysis" OR "Risk Assessment" OR "Statistical Correlation")

OR

MA("Statistics as Topic" OR Risk OR Odds Ratio)

OR

TI(risk* OR odds OR "cross-product ratio*" OR "hazards ratio*" OR "hazard ratio*" OR statistic* OR "HR" OR "RR" OR "aOR" OR relation* OR correlat* OR associat* OR likel*)

OR

AB(risk* OR odds OR "cross-product ratio*" OR "hazards ratio*" OR "hazard ratio*" OR statistic* OR "HR" OR "RR" OR "aOR" OR relation* OR correlat* OR associat* OR likel*)

OR

KW(risk* OR odds OR "cross-product ratio*" OR "hazards ratio*" OR "hazard ratio*" OR statistic* OR "HR" OR "RR" OR "aOR" OR relation* OR correlat* OR associat* OR likel*))

Limits: Publication Date: January 1970 – January 2023

Global Index Medicus Search String

(((mh:(I01.198.240.748* OR Violence OR I01.198.240.856.350* OR I01.198.240.856.463 OR I01.198.240.856.575* OR I01.198.240.856.688 OR I01.198.240.748.640 OR I01.198.240.856.825 OR I01.198.240.856.912 OR I01.198.240.856.519 OR M01.975.155 OR M01.135.500 OR I01.880.735.900.869 OR I01.880.735.305 OR SP9.020.800.010 OR "Harassment, Non-Sexual" OR "Aggression" OR I01.880.604.316 OR Dehumanization OR F01.145.813.191* OR I01.880.735.223.500 OR I01.880.735.035)) OR (ti:(violence OR "sexual assault" OR "sexual harassment" OR "sexual abuse" OR "sex abuse" OR rape OR "forced sex" OR "sexual coercion" OR "reproductive coercion" OR "sex trafficking" OR "sexual exploitation" OR "forced marriage" OR "forced marriages" OR "child marriage" OR "child marriages" OR "early marriage" OR "early marriages" OR "child bride" OR "child brides" OR CEFM OR "female genital mutilation" OR "female genital cutting" OR "female circumcision" OR "female genital circumcision" OR infibulation* OR clitoridectom* OR clitorectom* OR "ritual female genital surgery" OR "ritual female genital surgeries" OR FGM OR "physical abuse" OR "psychological abuse" OR "emotional abuse" OR "economic abuse" OR "financial abuse" OR "verbal abuse" OR maltreatment OR "violent discipline" OR "corporal punishment" OR "adverse childhood experience" OR "adverse childhood experiences" OR molestation OR "child abuse" OR "partner abuse" OR "dating abuse" OR "wife abuse" OR "spouse abuse" OR "domestic abuse" OR "elder abuse" OR "senior abuse" OR "aged abuse" OR victimization OR dehumanization OR victimisation OR dehumanisation OR stalking)) OR (ab:(violence OR "sexual assault" OR "sexual harassment" OR "sexual abuse" OR "sex abuse" OR rape OR "forced sex" OR "sexual coercion" OR "reproductive coercion" OR "sex trafficking" OR "sexual exploitation" OR "forced marriage" OR "forced marriages" OR "child marriage" OR "child marriages" OR "early marriage" OR "early marriages" OR "child bride" OR "child brides" OR CEFM OR "female genital mutilation" OR "female genital cutting" OR "female circumcision" OR "female genital circumcision" OR infibulation* OR clitoridectom* OR clitorectom* OR "ritual female genital surgery" OR "ritual female genital surgeries" OR FGM OR "physical abuse" OR "psychological abuse" OR "emotional abuse" OR "economic abuse" OR "financial abuse" OR "verbal abuse" OR maltreatment OR "violent discipline" OR "corporal punishment" OR "adverse childhood experience" OR "adverse childhood experiences" OR molestation OR "child abuse" OR "partner abuse" OR "dating abuse" OR "wife abuse" OR "spouse abuse" OR "domestic abuse" OR "elder abuse" OR "senior abuse" OR "aged abuse" OR victimization OR dehumanization OR victimisation OR dehumanisation OR stalking OR cyberviolence OR cybervictimization OR cyberstalking)))

AND

((mh:(E05.318.372.500.500* OR E05.318.370.150 OR E05.318.372.500.750* OR V03.850 OR L01.178.682.759.575 OR V03.600 OR E05.318.370.500* OR V03.900)) OR (ti:("systematic review" OR "meta-analysis" OR cohort OR "cross-over" OR "case-control" OR Prospective OR retrospective OR longitudinal OR "follow-up" OR followup)) OR (ab:("systematic review" OR "meta-analysis" OR cohort OR "cross-over" OR "case-control" OR Prospective OR retrospective OR longitudinal OR "follow-up" OR followup)))

AND

((mh:9E05.318.740* OR E05.318.740.600.800* OR E05.318.740.600.600)) OR (ti:(risk* OR odds OR "cross-product ratio" OR "cross-product ratios" OR "hazards ratio" OR "hazards ratios" OR "hazard ratio" OR "hazard ratios" OR statistic* OR "HR" OR "RR" OR "aOR" OR relation* OR correlat* OR associat* OR likel*) OR (ab:(risk* OR odds OR "cross-product ratio" OR "cross-product ratios" OR "hazards ratio" OR "hazards ratios" OR "hazard ratio" OR "hazard ratios" OR statistic* OR "HR" OR "RR" OR "aOR" OR relation* OR correlat* OR associat* OR likel*)))

)

AND

(year_cluster:[1970 TO 2023])

Cochrane Search String

([mh "Sex Offenses"] OR [mh ^"Violence"] OR [mh "Domestic Violence"] OR [mh "Gender-Based Violence"] OR [mh "Intimate Partner Violence"] OR [mh "Physical Abuse"] OR [mh "Rape"] OR [mh "Torture"] OR [mh "Workplace Violence"] OR [mh "Gun violence"] OR [mh "Battered Women"] OR [mh "Adult Survivors of Child abuse"] OR [mh "Exposure to Violence"] OR [mh "Emotional Abuse"] OR [mh "Sexual Harassment"] OR [mh ^"Harassment, Non-Sexual"] OR [mh ^"Aggression"] OR [mh "Coercion"] OR [mh "Dehumanization"] OR [mh "stalking"] OR [mh “adverse childhood experiences”] OR (violence OR "sexual assault" OR "sexual harassment" OR "sexual abuse" OR "sex abuse" OR rape OR "forced sex" OR "sexual coercion" OR "reproductive coercion" OR "sex trafficking" OR "sexual exploitation" OR ((forced OR child OR early) NEXT marriage*) OR (child NEXT bride*) OR CEFM OR "female genital mutilation" OR "female genital cutting" OR "female circumcision" OR "female genital circumcision" OR infibulation* OR clitoridectom* OR clitorectom* OR "ritual female genital surgery" OR "ritual female genital surgeries" OR FGM OR ((physical OR psychological OR emotional OR economic OR financial OR verbal) NEXT abuse) OR maltreatment OR "violent discipline" OR "corporal punishment" OR "adverse childhood experience" OR "adverse childhood experiences" OR molestation OR "child abuse" OR "partner abuse" OR "dating abuse" OR "wife abuse" OR "spouse abuse" OR "domestic abuse" OR "elder abuse" OR "senior abuse" OR "aged abuse" OR victimization OR dehumanization OR victimisation OR dehumanisation OR stalking OR cyberviolence OR cybervictimization OR cyberstalking):ti,ab,kw)

AND

([mh "Case-Control Studies"] OR [mh "Cross-Over Studies"] OR [mh "Cohort Studies"] OR [mh "Systematic Review"] OR [mh "Meta-Analysis"] OR [mh "Twin Study"] OR ("systematic review" OR "meta-analysis" OR "cohort" OR "cross-over" OR "case-control" OR "prospective" OR "retrospective" OR "longitudinal" OR "follow-up" OR "followup"):ti,ab,kw)

AND

([mh "Statistics as Topic"] OR [mh Risk] OR [mh "Odds Ratio"] OR ("risk" OR "odds" OR "cross-product ratio" OR "cross-product ratios" OR "hazards ratio" OR "hazards ratios" OR "hazard ratio" OR "hazard ratios" OR statistic* OR "HR" OR "RR" OR "aOR" OR relation* OR correlat* OR associat* OR likel*):ti,ab,kw)

Limits: January 1970 – January 2023

Web of Science Core Collection Search String

(TS=(

(violence OR "sexual assault" OR "sexual harassment" OR "sexual abuse" OR "sex abuse" OR rape OR "forced sex" OR "sexual coercion" OR "reproductive coercion" OR "sex trafficking" OR "sexual exploitation" OR "forced marriage*" OR “forced marriage*” OR “child marriage*” OR “early marriage*” OR “child bride*” OR CEFM OR "female genital mutilation" OR "female genital cutting" OR "female circumcision" OR "female genital circumcision" OR infibulation* OR clitoridectom* OR clitorectom* OR “ritual female genital surger*” OR FGM OR "physical abuse" OR "psychological abuse" OR "emotional abuse" OR "economic abuse" OR “financial abuse” OR "verbal abuse" OR Maltreatment OR torture OR "violent discipline" OR "corporal punishment" OR "adverse childhood experience*" OR molestation OR "child abuse" OR "partner abuse" OR "dating abuse" OR "wife abuse" OR "battered wom*n" OR "spouse abuse" OR "domestic abuse" OR "elder abuse" OR "senior abuse" OR "aged abuse" OR victimization OR dehumanization OR victimisation OR dehumanisation OR stalking OR cyberviolence OR cybervictimization OR cyberstalking)

AND

("systematic review" OR "meta-analysis" OR cohort OR cross-over OR case-control OR prospective OR retrospective OR longitudinal OR follow-up OR followup)

AND

("risk*" OR "odds" OR "cross-product ratio*" OR "hazards ratio*" OR "hazard ratio*" OR statistic* OR "HR" OR "RR" OR "aOR" OR relation* OR correlat* OR associat* OR likel*)

))

AND

DOP=(1970-01-01/2023-01-31)

Section 4.2: Inclusion and Exclusion Criteria

We used the criteria listed below to title and abstract screen 67,221 identified articles. Each review step (title/abstract screening, full-text screening, and data extraction) began with consensus building exercises across the review team. After training and consensus-building, the first two-thirds of titles/abstracts were reviewed by two independent reviewers, with conflicts resolved by project leaders. Upon confirmation of a low rate of total conflicts (<5% of total screened), the remainder of titles/abstracts were single screened. Non-English articles were screened by reviewers with proficiency in the language. Studies which met inclusion criteria during title/abstract screening (n = 4379) were full text screened and excluded if found to meet any exclusion criteria. Two independent reviewers full text screened 10% of articles, with conflicts resolved by project leads. Upon confirming a low conflict rate (<5%), the remaining 90% of articles were single screened.

Inclusion Criteria

Study design: case–control, cohort, or case-crossover studies.

Participants: Studies conducted in participant groups likely to be generalizable to the population of interest. Exposed groups are defined as any individual who has experienced a form of GBV and/or VAC throughout the lifetime. Comparators will be non-exposed control groups, or study groups without reported exposure to a form of GBV and/or VAC.

Outcomes: Studies reporting an estimate of association (either RR, risk ratio, odds ratio, hazard ratio or similar) or reporting cases and non-cases among those exposed and unexposed. If not provided directly, studies providing enough information to allow an estimate of RR to be calculated will meet inclusion criteria.

Exclusion criteria

Study design: Cross-sectional, ecological, case series or case studies.

Participants: Studies conducted in subgroups identified only by convenience sampling or subgroups identified via a shared characteristic that is likely related to risk of exposure to violence or the reported health outcome (e.g., domestic violence shelter residents).

Exposure measurement: Studies that report only an aggregate measure of exposure combining exposure to a form of violence with other, non-eligible exposures (e.g., reports a composite ACE score only) will be excluded. For these studies, we are unable to disentangle the effect of violence exposure from the effects of other hardships or exposure types, preventing their inclusion in our review.

Does not meet minimum reporting criteria: Studies missing essential data, that is, those that do not report effect sizes and uncertainty information (confidence intervals, sample sizes) or the data needed to impute an effect size with uncertainty information.

Section 4.3: Systematic review and meta-analysis citation searching

We title and abstract and full text screened systematic reviews and/or meta-analyses yielded by our searches according to the criteria outlined for all other articles. In total, we accepted 771 systematic reviews and/or meta-analyses during title/abstract screening. Of these, we accepted 267 in full-text screening.

We then grouped accepted systematic reviews/meta-analyses by unique risk-outcome pair combination (e.g., IPV-anxiety disorders; CSA-alcohol use disorders) in order to extract the citations identified by these reviews. If more than one systematic review was identified for a given risk-outcome pair, one systematic review was selected for citation searching on the basis of publication recency, number of included underlying studies and study quality. Study quality was determined via adherence to PRISMA and GATHER guidelines and by the impact factor of the journal the study was published within.

Once a single systematic review was selected for citation searching per unique risk-outcome pair, we extracted the references identified within each review and de-duplicated them against our primary search records. All new articles from this search were then screened for inclusion with the same criteria as in our larger review (see supplementary section 4.2).

Across all risk outcome pairs, we extracted 1202 articles, 584 of which were not duplicated with our own search records. We accepted 207 articles in title/abstract screening and 38 articles in full-text screening. Thus, this citation-searching step of our review yielded 38 new studies for extraction.

Section 4.4: Data Extraction

All studies were extracted using a modified data extraction template in Covidence data extraction 2.0. Table S20 details the fields extracted for each article.

Table S20: Data Extraction Template

Name		Definition
**Study locations**	**Location Name**	The country or IHME sub-national location where the study took place
	**Location ID**	The location ID corresponding to the country or IHME sub-national location of the study
**Specific Location**		Required if the study took place in a location smaller than the corresponding location name and ID
**Study Name**		Required if cohort is named, do not use the article title. This field is used to screen out duplicative data, so please ensure spelling is correct.
**Year Start**		Year the study started
**Year End**		Year the study ended (NOT publication year)
**Study Design**	**Prospective cohort**	Must select one
	**Retrospective cohort**	

	Case-control	
	Case-cohort	
	Case-crossover	
**Pooled cohort**		Cohort studies only: Yes if the reported effect size is from a pooled analysis and only pooled effect size has been reported, otherwise no
**Study Selection Criteria**		Please specify the selection criteria of the study that is used in the analysis
**Location Representative**		Specify if the participants were representative of the study's geography or not
**Cohort Study: Drop-out rate**		Study dropout rate (%) at the end of the study entered as a decimal
**Cohort Study: Drop-out rate assessment**		Specify how dropout rate was defined in the study.
**Cohort Study: Follow-up measure**		Cohort studies only: Type of follow up measure reported (eg, 'average participant follow-up was 126 days' then select 'mean').
**Cohort Study: Follow-up units**		Cohort studies only: enter units of follow-up duration reported (eg, 'average participant follow-up was 126 days' then enter 'days').
**Cohort Study: Follow-up value**		Cohort studies only: Enter the length of participant follow-up if reported (eg, 'average participant follow-up was 126 days' then enter 126).
**Case-control Study: Percent of participants for which data ascertained**		Percent of participants (%) from total, for which the study has included data entered as a decimal
**Case-control Study: Controls selected from community**		Were the controls selected from the community? Yes or No
**Exposure assessment method**		Self-report, routinely collected/ administrative data, clinical examination
**Exposure assessment instrument**		Specify the name of the exposure assessment instrument. For self-reported exposures, please specify the name of the questionnaire. If more than one instrument, specify all. If the instrument is not names/designed specifically for the study, write "study-specific instrument"
**Exposure assessment period**		How many times information on exposure to type of violence
**Exposure assessment value**		If "exposure assessment period" is multiple, specify the number of times that exposure was assessed (excluding baseline)
**Outcome assessment method**		Select how the study ascertained which participants experienced the outcome
**Outcome assessment instrument**		Specify the name of the outcome assessment instrument. For self-reported outcomes, please specify the name of the questionnaire.If more than one instrument, specify all. If the instrument is not names/designed specifically for the study, write "study-specific instrument"
**Effect size measure**		Select the form of effect size used in the study

**Uncertainty type**		
**Confidence interval level**		If uncertainty is reported as a confidence interval, this column represents the confidence level which is reported at (Eg. 95, 90, 99).
**Extractor notes**		Please use this field to include any notes about the study or your extraction not covered elsewhere.
**Outcome name**		Outcome that is measured in this model
**Outcome type**		Please specify if the outcome definition included incidence of or mortality from a disease endpoint.
**Outcome definition**		Please specify the definition for the outcome as reported in the study.
**Exposure definition**		Please specify the definition for the exposed participants exactly as reported in the study.
**Exposed level**		Enter level of exposure defined for exposed group. If study's exposure is binary (any vs. none), select 'any exposure' for exposed. For all other exposure levels, select other and enter level as reported (eg, 'exposed 3-5 times').
**Unexposed definition**		Provide a brief description of the unexposed group (i.e., the comparison group) as used in estimation of the relative risk
**Unexposed level**		Enter level of exposure defined for unexposed (comparator group). If the study's exposure is binary (any vs. none), select 'no exposure' for unexposed. For all other exposure levels, select other and enter level as reported (eg, 'exposed 1-2 times').
**Violence type**		Select all violence types included for this model
**Violence type combination**		Select HOW the violence types are combined in the model.<br>(1) AND: the model must specify that the exposure group experienced BOTH/ALL types of violence selected in the previous question<br>(2) AND/OR: the exposure group consists of people who experience EITHER type of violence<br>(3) ONLY: model specifies that the exposure group experienced selected violence type, but not other types of violence included in the study.<br><br>*NOTE: Use 'only' option when it has been confirmed that is the ONLY type of violence a participant experienced (eg, experienced sexual but NOT physical violence). Otherwise, if one type of violence assessed and exposure to others are unknown, use 'unknown'.*
**Perpetrator type**		Select all perpetrator types included in this model
**Temporality of Exposure**	**Lower (Age)**	Lower bound of age range provided of when participants experienced violence. If no lower age is provided, fill in this box with '0' and provide more information in the "other information" question for this model (e.g. "temporality of exposure defined as "Childhood and adolescence")

	**Upper (Age)**	Upper bound of age range provided of when participants experienced violence. If no upper age is provided, fill in this box with "99" and provide more information in the "other information" question for this model (e.g. "temporality of exposure defined as "Childhood and adolescence")
**Exposure Recall Type**		Specify whether the exposure occurred throughout lifetime, past year, or indicate other recall type.
**Percent Women or Female**		For the sample of this model, what percent are female or women (0-1) on a per 1 basis (eg, 43% female should be recorded as 0.43). Enter 1 if sample is only female. Enter 0 if sample is only male. If sample includes both sexes or genders and percent female not reported, enter 99.
**Ages**	**Lower**	Lower bound of age of participants included in this model at time of study. If the model includes participants of all ages from the study,this box will match the lower age box in section 2.
	**Upper**	Upper bound of age of participants included in this model at time of study. If the model includes participants of all ages from the study,this box will match the upper age box in section 2.
	**Mean**	Mean of age of participants included in this model.
	**SD**	SD of age of participants included in this model.
**Subgroup analysis**		**PER UNIQUE RISK-OUTCOME PAIR IN A STUDY:** Yes if this effect size is a sub-analysis reported IN ADDITION TO a main analysis from all participants (eg, study reports effect size for combined sexes and also effect sizes separately for males and females). If study only reports effect sizes from specific subgroups (eg, reports effect sizes from males and females separately, without reporting a combined effect size), select no.
**Subgroup analysis free text**		If a sub-analysis, describe stratifier (i.e. age, sex, etc.)
**Effect size**	**Mean**	Mean effect size of model
	**Lower (UI)**	Lower bound of effect size
	**Upper (UI)**	Upper bound of effect size
	**Other uncertainty value**	
**Effect size table #**		Table number where you found effect size from literature
**Sample size**	**Number of cases**	Enter number of participants with measured outcome for each group reported: exposed, unexposed, total
	**Number of participants**	Enter number of participants included in analysis for each group reported: exposed, unexposed, total
	**Person-time (cohort studies)**	Enter person-time for each group reported: exposed, unexposed, total

**Person-time units (cohort studies)**	If cohort study and person-time entered into sample size table, specify the units of person-time reported
**Confounders**	Select all confounders for the most adjusted model, write in any confounders (separated by comma) not included in pre-specified list
**Other information**	Any additional information

Section 4.5: Exposure and Outcome Definitions

The exposures examined in this review were defined in accordance with the risk factor definitions used in the GBD study (Table S21).

In forming our input datasets for IPV models, we accepted author definitions of IPV exposure matching the GBD case definition (i.e., physical and/or IPV), those which measured physical IPV only, and those which measured sexual IPV only. Due to data sparsity, we additionally accepted studies with author definitions which included psychological violence in addition to physical and/or sexual (i.e., defined exposure as any intimate partner violence involving physical, sexual, and/or psychological abuse). Potential bias due to using an accepted alternate exposure definition was accounted for in our modeling process via two study-level bias covariates marking component exposure definitions and aggregate exposure definitions (i.e., those including psychological violence). We did not include author definitions measuring psychological IPV only, economic/financial IPV only, nor those reporting aggregate definitions incorporating economic IPV.

In forming our input datasets for CSA models, we accepted author definitions of CSA exposure using any age threshold ≤ 18 years by any perpetrator. The GBD case definition considers sexual abuse less than 15 years old; for definitions which used alternate ages of exposure, we incorporated a study-level bias covariate to test the impact of setting different upper bounds of the ages which constitute childhood. In addition, certain studies measured CSA perpetrated in the context of specific relationships (i.e., family member-perpetrated CSA); estimates from these studies were marked with a bias covariate indicating that a restricted perpetrator definition was used.

Table S21: Definitions of included risk factors.

Risk factor name	Definition
**Intimate partner violence**	Lifetime prevalence of physical and/or sexual violence by a current or former intimate partner since age 15
**Childhood sexual abuse**	Lifetime prevalence of intercourse or other contact abuse (i.e., fondling or other sexual touching) when aged 15 years or younger in which the contact was unwanted, or perpetrator was 5+ years older than the victim

The outcomes examined in this review were defined in accordance with the cause definitions used in the GBD study (Table S22). We followed cause-specific research team guidance to accept GBD reference and alternate case definitions. For accepted alternate case definitions, we incorporated study-level bias covariates to detect if the use of an alternate definitions significantly biased final model results.

Table S22: Definitions of included outcomes.

Cause Grouping	Cause Name	Definition
**Mental health disorders**	Major depressive disorder	Major depressive disorder assessed according to DSM-4 (296.21–24, 296.31–34) and ICD-10 criteria (F32.0–9, F33.0–9). Diagnostic interviews and symptom scales were accepted according to the GBD criteria (Supplemental Table 4).

	Anxiety disorders	Anxiety disorders involving experiences of intense fear and distress in combination with other physiological symptoms.
	Conduct disorder	Conduct disorder occurs in those under 18 years of age and incorporates disability from antisocial behavior that violates basic rights of others or major age-appropriate societal norms. DSM-IV-TR (312.81–312.89) and ICD-10 (F91) diagnostic criteria were used.
	Bulimia nervosa	Bulimia nervosa is an eating disorder, incorporating deaths and disability from recurrent binge eating, inappropriate compensatory behavior to prevent weight gain, and undue influence of body shape and weight on self-evaluation. DSM and ICD diagnostic criteria were used.
	Anorexia nervosa	Anorexia nervosa is an eating disorder incorporating deaths and disability from refusal to maintain minimally normal body weight, intense fear of gaining weight, and disturbances in how one's body weight or shape is experienced. DSM and ICD diagnostic criteria were used.
	Schizophrenia	Schizophrenia is a chronic psychotic disorder that involves positive symptoms (eg, delusions, hallucinations) and negative symptoms (eg, flat affect, loss of interest). DSM-IV-TR (295.10–295.30, 295.60, 295.90) and ICD-10 (F20) criteria were used.
**Substance use disorders**	Alcohol use disorder	A maladaptive pattern of substance use, leading to clinically significant impairment or distress, as manifested by three (or more) of the DSM-4 criteria for substance dependence occurring any time in a 12-month period.
	Drug use disorders	This aggregate cause incorporates death and disability resulting from opioid use disorder, amphetamine use disorder, cocaine use disorder, cannabis use disorder, and a residual category of other drug use disorders including deaths and disability due to dependence on hallucinogens, inhalants, solvents, and sedatives.
**Maternal disorders**	Maternal abortion and miscarriage	Abortion is defined as elective or medically indicated termination of pregnancy at any gestational age and miscarriage is defined as spontaneous loss of pregnancy before 24 weeks of gestation with complications requiring medical care.
**HIV/AIDS and Sexually Transmitted Infections**	HIV/AIDS	HIV/AIDS as assessed by biomarker tests.
	Sexually transmitted infections excluding HIV	Sexually transmitted infections are viral, bacterial, or parasitic infections that are transmitted through sexual contact. This aggregate group includes syphilis, chlamydia, gonorrhea, genital herpes, trichomoniasis,

		and a residual category of other sexually transmitted infections including chancroid, granuloma inguinale, and unspecified sexually transmitted diseases.
**Injuries**	Self-harm	Self-harm is deliberate bodily damage inflicted on oneself resulting in death or injury (ICD-9: E950–E959, ICD-10: X60–X64.9, X66–X84.9, Y87.0).
**Chronic respiratory diseases**	Asthma	Asthma is a chronic lung disease characterized by reversible airway obstruction due to spasms and secretions in the bronchi usually resulting from an allergic reaction or hypersensitivity and causing difficulty in breathing.
**Diabetes and kidney diseases**	Type 2 diabetes mellitus	Type 2 diabetes is a chronic condition, mostly in adults, where the body forms a resistance to insulin or the pancreas stops producing enough insulin. GBD defines type 2 diabetes as fasting plasma glucose of at least 7 mmol/L (126 mg/dL) or those currently treated with drugs or insulin.
**Cardiovascular diseases**	Ischemic heart disease	Ischemic heart disease is a disease of the coronary arteries, usually from atherosclerosis, leading to myocardial infarction or ischemia, following the Fourth Universal Definition of Myocardial Infarction and, for stable angina, physician diagnosis.

Section 4.5.1: Additional description of accepted definitions and measurement tools for depressive and anxiety disorders

For depressive and anxiety disorders specifically, we received an inventory of acceptable diagnostic interview and symptom scales from the mental health research team at IHME. These tools have been reviewed in terms of their validity and specificity to measuring the symptoms of these specific disorders. We included studies using accepted diagnostic interviews (reference) or symptom scales (accepted alternate). A list of acceptable tools is included below. Studies using symptom scales were marked with a study-level bias covariate.

For other mental disorders, we followed GBD case definitions and accepted studies measuring outcomes by use of International Disease Classification and Diagnostic and Statistical Manual of Mental Disorder criteria.

Depressive and anxiety disorder accepted diagnostic interview and symptom scale measurement tools.

List of accepted diagnostic interview tools (reference method of measurement)

- Structured Clinical Interview for DSM-IV Axis Disorders (SCID-I) (1 month, lifetime)
- Structured Clinical Interview for DSM-IV-TR Research Version, Non-patient edition (SCID-I/NP).
- Schedules for Clinical Assessment in Neuropsychiatry (SCAN)
- Mini International Neuropsychiatric Interview (MINI) (2 weeks, 2Y dysthymia)
- Mini International neuro-psychiatric Interview for children and Adolescents (MINI-KID)
- Diagnostic Interview Schedule (DIS) or Diagnostic Interview Schedule-IV (DIS-IV)
- Chinese modified Diagnostic Interview Schedule (DIS-CM)
- Composite International Diagnostic Interview (CIDI) (1 year, lifetime)
- University of Michigan Composite International Diagnostic Interview (UM-CIDI)

- Korean version of the Composite International Diagnostic Interview (K-CIDI)
- Munich-Composite International Diagnostic Interview (M-CIDI)
- Geriatric Mental State Schedule (GMS) (1 month) - AGECAT (Automated Geriatric Examination for Computer Assisted Taxonomy)
- Primary Care Evaluation of Mental Disorders (PRIME-MD)
- Development and Well-Being Assessment (DAWBA)
- Schedule for Affective Disorders and Schizophrenia (SADS)
- Kiddie Schedule for Affective Disorders and Schizophrenia (K-SADS-PL)
- Comprehensive Psychopathological Rating Scale (CPRS)
- The Alcohol Use Disorder and Associated Disabilities Interview Schedule-IV (AUDADIS-IV) (1 year, lifetime)
- Diagnostic Interview for Children and Adolescents (DICA)
- SPIKE interview
- Clinical Interview Schedule-Revised (CIS-R) (1 week)
- Diagnostic Interview Schedule for Children Version IV (DISC)
- Diagnostic interview schedule for children-young child version (DISC-YC)
- Diagnostic Interview Schedule for Children, Parent Report (DISC-P)
- Present state examination (PSE)
- Child and adolescent psychiatric assessment (CAPA)
- Preschool age psychiatric assessment (PAPA)
- Children's Depression Rating Scale—Revised (CDRS-R)

List of accepted symptom scale tools (alternate method of measurement)

- Patient Health Questionnaire (PHQ)
- Revised Brief Patient Health Questionnaire (Brief PHQ-R)
- Beck Depression Inventory (BDI)
- Center for Epidemiologic Studies Depression Scale (CES-D)
- Center for Epidemiologic Studies Depression Scale for Children (CES-DC)
- Hospital Anxiety and Depression Scale (HADS)
- Depression Anxiety Stress Scale (DASS)
- Reynolds Adolescent Depression Scale (RADS)
- Child Depression Inventory (CDI)
- Duke Anxiety-Depression scale (DUKE-AD)
- Emotional State Questionnaire (EST-Q)
- Hopkins Symptom Checklist (HSCL) -DMI (25 items)
- Health & Daily Living Form (HDL)
- Child behavior checklist (CBCL)
- Hamilton depression rating scale (HAM-D)
- Harvard Department of Psychiatry National Depression Screening Day Scale (HANDS)
- Children’s Depression Scale (CDS)
- Major Depression Inventory (MDI)
- Quick Inventory of Depressive Symptomology (QIDS)

Section 4.5.2: Additional description of accepted definitions for substance use disorders

For all the substance use disorders modeled in the GBD, the base reference case definitions are the DSM-4 criteria for substance dependence. Dependence is defined as a maladaptive pattern of substance use, leading to clinically significant impairment or distress, as manifested by three (or more) of the following, occurring any time in a 12-month period:

- Tolerance, as defined by either of the following: (a) a need for markedly increased amounts of the substance to achieve intoxication or desired effect, or (b) markedly diminished effect with continued use of the same amount of the substance.

- Withdrawal, as manifested by either of the following: (a) the characteristic withdrawal syndrome for the substance, or (b) the same (or closely related) substance is taken to relieve or avoid withdrawal symptoms.
- The substance is often taken in larger amounts or over a longer period than intended.
- There is a persistent desire or unsuccessful efforts to cut down or control substance use.
- A great deal of time is spent in activities necessary to obtain the substance, use the substance, or recover from its effects.
- Important social, occupational, or recreational activities are given up or reduced because of substance use.
- The substance use is continued despite knowledge of having a persistent physical or psychological problem that is likely to have been caused or exacerbated by the substance (e.g., current cocaine use despite recognition of cocaine-induced depression, continued drinking despite recognition that an ulcer was made worse by alcohol consumption)

DSM-4 also provides the diagnostic criteria for substance abuse. The criteria for substance abuse focus on social and situational consequences of use and does not mention withdrawal or tolerance. For alcohol use disorder specifically, we accepted author definitions of alcohol abuse and/or dependence. We did not accept author definitions measuring 'problematic' or 'harmful' alcohol use via the alcohol use disorders identification test (AUDIT). For drug use disorders we also include data on regular use, defined as how many cases use the substance weekly. Definitions described here which are not specifically measuring dependence are considered alternate accepted definitions and marked with a corresponding bias covariate.

Section 5: Supplementary Methods

Section 5.1: Definitions of bias covariates

Following GRADE criteria, the risk of bias criteria for individual studies included in our analyses captured representativeness of the study population, exposure and outcome measurement quality, control for confounding, selection bias, and risk of reverse causation. Because our analyses covered two distinct risk factors and many different health outcomes, we created a core set of bias covariates across all risk-outcomes pairs (Table S25) as well as additional bias covariates specific to CSA analyses (Table S24; Table S27), IPV analyses (Table S23; Table S26) and the different health outcomes selected for analyses (Table S28). For all covariates, the reference value is zero while indication of the specific bias type was coded as a one. All covariates meeting eligibility requirements (i.e., at least two studies represented for each value of the covariate) were tested for significance using the selection algorithm in the MR-BRT tool. Minimum availability of two observations for each value of the covariate meant that a reduced set of covariates were able to be tested for risk-outcome pairs with low total study counts.

Table S23. IPV-specific bias covariates.

Bias category	**Bias covariate name**	**Description**	**Values**

**Exposure measurement**	Component_exp_def	Author definition of IPV measured sexual violence only or physical violence only.	0 if no; 1 if yes
	Aggregate_exp_def	Author definition of IPV includes psychological violence.	0 if no; 1 if yes
	non_lifetime_recall	Author definition of IPV measure exposure over a time period shorter than lifetime (e.g., past year, past 3 years, past 6 months)	0 if no; 1 if yes

Table S24. CSA-specific bias covariates.

Bias category	Bias covariate name	Description	Values
**Representativeness of study population**	men_only women_only	Covariates included for CSA analyses only (IPV analyses are only among women samples). Captures if sample consists of men only, women only, or both men and women.	men_only = 1 if male only study sample women_only = 1 if female only study sample both covariates = 0 if study reports effect size for men and women or two effect sizes separately by gender
**Exposure measurement**	admin_exp_method	Captures if exposure was ascertained from administrative databases (health systems, agency records, etc.), since many children experiencing sexual abuse would not be reported/investigated through official channels.	1 if study used administrative source for exposure ascertainment, 0 if not (self-report)
	Restricted_perpetrator	Captures if exposure to CSA was measured in the context of specific relationships (i.e., perpetrated by family member or caregiver).	1 if study used restricted perpetrator identity; 0 if not
	Exp_before_agebelow15	Captures if exposure to sexual abuse/violence was measured	1 if study used upper

		prior to an age less than 15 (i.e., experiences before age 12).	exposure age below 15; 0 if measured before age 15
	Exp_before_ageabove15	Captures if exposure to sexual abuse/violence was measured prior to an age above than 15 (e.g., experiences before age 18).	1 if study used upper exposure age above 15; 0 if measured before age 15

Table S25. Standard bias covariates created across all input datasets.

Bias category	Bias covariate name	Description	Values
**Representativeness of study population**	representativeness	Study sample is not geographically representative of underlying location	0 if no; 1 if yes
	subpop	Study sample represents a sub-population, e.g., men who have sex with men	0 if no; 1 if yes
**Selection bias**	selection_bias	Captures if study is at risk for selection bias. Study determined to be at risk for selection bias if loss to follow-up (cohorts) or percent for whom data not ascertained (case-controls) > 20%.	0 if no; 1 if yes
**Reverse causation**	reverse_causation	Captures if study is at risk of reverse causation. Case-control studies are assumed to be at risk of reverse-causation.	0 if no; 1 if yes
**Control for confounding**	confounding_uncontrolled	Captures if a study reports completely unadjusted estimates of association (raw or crude effect sizes).	0 if no; 1 if yes
**Other**	odds_ratio	Study reports an odds ratio (reference: relative risk).	0 if no; 1 if yes

Table S26. Bias covariates for control for confounding used in intimate partner violence analyses.

Level of adjustment	Definition	Variables and values	
		Age_uncontrolled	L1
**Insufficient**	Does not control for age, regardless of control for other variables	1	1

**Middle**	Controls for age and 1-2 other confounders	0	1
**Optimal**	Controls for age and 3+ confounders	0	0

Table S27. Bias covariates for control for confounding used in childhood sexual abuse analyses.

Level of adjustment	Definition	Variables and values		
		Age_uncontrolled	Sex_uncontrolled	L1
**Insufficient**	Does not control for age or sex, regardless of control for other variables	1	1	1
**Middle**	Controls for age and sex only	0	0	1
	Controls for age only	0	1	1
	Controls for sex only	1	0	1
**Optimal**	Controls for age, sex, and at least one additional confounder	0	0	0

Table S28. Outcome-specific bias covariates.

Outcome	Bias covariate	Description	Values
**Major depressive disorder**	symptom_scale	Study used a symptom scale (see list of accepted symptom scales in Table S4) to measure depressive disorders. Reference measurement tool is a diagnostic instrument.	0 if no (diagnostic interview); 1 if yes (symptom scale)
	aggregate_outcome_def	Study measured depressive disorders rather than major depressive disorder specifically.	0 if no (major depressive disorder); 1 if yes (depressive disorders in aggregate)
	baseline_uncontrolled	**IPV analyses only**: study did not control for baseline diagnoses	0 if no; 1 if yes

		of depressive disorders.	
	Lifetime_diagnoses	<u>**CSA analyses only:**</u> Study measured prevalent depression diagnoses rather than incident diagnoses.	0 if no; 1 if yes
**Anxiety disorders**	Component_outcomedef	Study measured a specific anxiety disorder (e.g. PTSD, generalized anxiety disorder) rather than any anxiety disorder	0 if no; 1 if yes
**Alcohol use disorder**	alternate_outcomedef	Study measured alcohol abuse and/or dependence rather than explicitly alcohol dependence (GBD reference definition)	0 if no; 1 if yes
**Drug use disorders**	use	Study measured illicit drug use without measuring use disorder according to DSM criteria.	0 if no; 1 if yes
	specific_substance	Study measured specific drug use disorder rather than any drug use disorder.	0 if no; 1 if yes
	cannabis	Study measured cannabis use / use disorder.	0 if no; 1 if yes
**Self-harm**	lifetime_attempts	Study assessed 'ever' suicide attempts rather than incident attempts.	0 if no; 1 if yes
**Type 2 diabetes mellitus**	type_nonspecific	Study measured diabetes mellitus without specifying type 2.	0 if no; 1 if yes
	prevalent_diabetes	Study measured diabetes prevalence, not incidence.	0 if no; 1 if yes
**Asthma**	Regular_symptoms	Study required presence of regular/semi-regular symptoms (e.g., wheezing, need for	0 if no; 1 if yes

		inhaler) in addition to asthma diagnosis for positive cases	

Section 5.2: Data adjustment for non-mutually exclusive observations from a single study

In certain cases, we used multiple data points from a single study within our input modeling set. If observations reflected different participants/subgroups (i.e., sex-stratified or age-stratified effect sizes), we included all observations from the study without adjusting the standard error.

If observations reflected non-mutually exclusive measurement using the same participants (i.e., study reported effect size for physical IPV exposure [regardless of sexual IPV exposure] compared to unexposed *and* sexual IPV exposure [regardless of physical IPV exposure] compared to unexposed), we included each observation but adjusted standard errors using the following formula:

$$adjusted\ standard\ error = \sqrt{original\ standard\ error^2 * scalar}$$

where scalar = number of observations per unique participant group.

This approach ensured that the studies using repeated measurements were not over-represented in the final model.

Lastly, if observations reflected different, mutually exclusive exposure levels using the same referent group (i.e., exposed once vs. never exposed, exposed twice vs. never exposed, etc.), we included each of the multiple levels from a single study (which together make up any exposure) and did not adjust standard error.

Section 6: Study Quality and Bias Assessment

We report the bias covariates marked and tested for each IPV risk-outcome pair in Table S29 and each CSA risk-outcome pair in Tables S30a and S30b. N/A indicates that there were not enough studies within the risk-outcome pair combination to test the respective bias covariate; 1 indicates that the bias covariate applies to the study for that risk-outcome pair; 0 indicates that the bias covariate does not apply to the study for that risk-outcome pair.

Table S29: Study bias characteristics for intimate partner violence.

Author	Health outcome	Study reports odds ratio	Study at risk of selection bias	Uncontrolled for age	Represents a subpopulation	Cases ascertained from clinical sample	Study at risk of reverse causation	Uncontrolled for any confounders	Exposure definition is a component of reference definition	Measured recent/current IPV (rather than lifetime)	Sample not geographically representative	Used symptom scaled to ascertain depression	Outcome definition is a component of reference definition	Uncontrolled more than 2 covariates beyond age	Outcome definition is aggregate of reference	Exposure definition includes psychological violence
Ahmadabadi 2020	Anxiety disorders	N/A	1	N/A	0	N/A	N/A	N/A	1	0	1	0	0	0	N/A	N/A
Brown 2020	Anxiety disorders	N/A	1	N/A	1	N/A	N/A	N/A	0	1	1	1	0	0	N/A	N/A
Ehrensaft 2006	Anxiety disorders	N/A	0	N/A	0	N/A	N/A	N/A	1	1	0	0	1	1	N/A	N/A
Makaroun 2020	Anxiety disorders	N/A	0	N/A	1	N/A	N/A	N/A	0	1	1	0	0	1	N/A	N/A
Suglia 2011	Anxiety disorders	N/A	1	N/A	1	N/A	N/A	N/A	0	0	0	1	1	0	N/A	N/A
Delong 2019	HIV/AIDS	0	0	0	0	N/A	N/A	N/A	N/A	N/A	N/A	N/A	N/A	N/A	N/A	N/A
Deyessa 2018	HIV/AIDS	1	0	0	0	N/A	N/A	N/A	N/A	N/A	N/A	N/A	N/A	N/A	N/A	N/A
Fonck 2005	HIV/AIDS	1	0	1	1	N/A	N/A	N/A	N/A	N/A	N/A	N/A	N/A	N/A	N/A	N/A
Jewkes 2010	HIV/AIDS	0	0	0	0	N/A	N/A	N/A	N/A	N/A	N/A	N/A	N/A	N/A	N/A	N/A
Kouyoumdjian 2013	HIV/AIDS	0	1	1	0	N/A	N/A	N/A	N/A	N/A	N/A	N/A	N/A	N/A	N/A	N/A
Maman 2002	HIV/AIDS	1	1	0	1	N/A	N/A	N/A	N/A	N/A	N/A	N/A	N/A	N/A	N/A	N/A
Ahmadabadi 2020	Major depressive disorder	N/A	1	0	0	N/A	0	0	0	0	1	0	N/A	0	0	0

Author	Health outcome	Study reports odds ratio	Study at risk of selection bias	Uncontrolled for age	Represents a subpopulation	Cases ascertained from clinical sample	Study at risk of reverse causation	Uncontrolled for any confounders	Exposure definition is a component of reference definition	Measured recent/current IPV (rather than lifetime)	Sample not geographically representative	Used symptom scaled to ascertain depression	Outcome definition is a component of reference definition	Uncontrolled more than 2 covariates beyond age	Outcome definition is aggregate of reference	Exposure definition includes psychological violence
Ali 2009	Major depressive disorder	N/A	0	1	1	N/A	1	1	0	0	1	0	N/A	1	1	1
Ali 2009	Major depressive disorder	N/A	0	0	1	N/A	1	0	0	0	1	0	N/A	0	1	1
Brown 2020	Major depressive disorder	N/A	1	0	1	N/A	0	0	0	1	1	1	N/A	0	0	1
Chowdhary 2008	Major depressive disorder	N/A	0	0	1	N/A	0	0	0	0	1	0	N/A	1	1	0
Ehrensaft 2006	Major depressive disorder	N/A	0	0	0	N/A	0	0	1	1	0	0	N/A	1	0	0
Han 2019	Major depressive disorder	N/A	0	0	1	N/A	0	0	1	1	0	1	N/A	0	1	0
Llosamartínez 2019	Major depressive disorder	N/A	1	1	0	N/A	1	1	0	1	1	1	N/A	1	1	1
Makaroun 2020	Major depressive disorder	N/A	0	0	1	N/A	0	0	0	1	1	0	N/A	1	1	0
Ouellet-Morin 2015	Major depressive disorder	N/A	0	1	1	N/A	0	0	0	1	0	0	N/A	1	1	1
Pico-Alfonso 2006	Major depressive disorder	N/A	0	1	0	N/A	1	1	0	0	1	1	N/A	1	1	1

Author	Health outcome	Study reports odds ratio	Study at risk of selection bias	Uncontrolled for age	Represents a subpopulation	Cases ascertained from clinical sample	Study at risk of reverse causation	Uncontrolled for any confounders	Exposure definition is a component of reference definition	Measured recent/current IPV (rather than lifetime)	Sample not geographically representative	Used symptom scaled to ascertain depression	Outcome definition is a component of reference definition	Uncontrolled more than 2 covariates beyond age	Outcome definition is aggregate of reference	Exposure definition includes psychological violence
Suglia 2011	Major depressive disorder	N/A	1	0	1	N/A	0	0	0	0	0	1	N/A	0	1	0
Taft 2008	Major depressive disorder	N/A	1	0	0	N/A	0	0	0	0	0	1	N/A	0	1	0
Abdollahi 2015	Maternal abortion and miscarriage	N/A	N/A	0	N/A	N/A	0	0	0	1	N/A	N/A	N/A	N/A	N/A	N/A
Bourassa 2007	Maternal abortion and miscarriage	N/A	N/A	1	N/A	N/A	1	1	0	1	N/A	N/A	N/A	N/A	N/A	N/A
Catak 2016	Maternal abortion and miscarriage	N/A	N/A	1	N/A	N/A	1	0	1	0	N/A	N/A	N/A	N/A	N/A	N/A
Ibrahim 2015	Maternal abortion and miscarriage	N/A	N/A	1	N/A	N/A	0	1	1	1	N/A	N/A	N/A	N/A	N/A	N/A
Johri 2011	Maternal abortion and miscarriage	N/A	N/A	0	N/A	N/A	1	0	0	1	N/A	N/A	N/A	N/A	N/A	N/A
Leung 2002	Maternal abortion and miscarriage	N/A	N/A	1	N/A	N/A	1	1	0	1	N/A	N/A	N/A	N/A	N/A	N/A
Nelson 2003	Maternal abortion and miscarriage	N/A	N/A	1	N/A	N/A	1	1	1	0	N/A	N/A	N/A	N/A	N/A	N/A
Romito 2009	Maternal abortion and miscarriage	N/A	N/A	1	N/A	N/A	1	0	0	1	N/A	N/A	N/A	N/A	N/A	N/A

Author	Health outcome	Study reports odds ratio	Study at risk of selection bias	Uncontrolled for age	Represents a subpopulation	Cases ascertained from clinical sample	Study at risk of reverse causation	Uncontrolled for any confounders	Exposure definition is a component of reference definition	Measured recent/current IPV (rather than lifetime)	Sample not geographically representative	Used symptom scaled to ascertain depression	Outcome definition is a component of reference definition	Uncontrolled more than 2 covariates beyond age	Outcome definition is aggregate of reference	Exposure definition includes psychological violence
Taft 2019	Maternal abortion and miscarriage	N/A	N/A	0	N/A	N/A	0	0	0	0	N/A	N/A	N/A	N/A	N/A	N/A
Ackard 2007	Self-harm	N/A	1	N/A	N/A	0	N/A	N/A	N/A	N/A	N/A	N/A	N/A	N/A	N/A	N/A
Chowdhary 2008	Self-harm	N/A	0	N/A	N/A	0	N/A	N/A	N/A	N/A	N/A	N/A	N/A	N/A	N/A	N/A
Kaslow 2000	Self-harm	N/A	1	N/A	N/A	1	N/A	N/A	N/A	N/A	N/A	N/A	N/A	N/A	N/A	N/A
Makaroun 2020	Self-harm	N/A	0	N/A	N/A	1	N/A	N/A	N/A	N/A	N/A	N/A	N/A	N/A	N/A	N/A

N/A, not available across minimum number of studies needed and not tested for given risk outcome pair. 1 = bias covariate applies to the study; 0 = bias covariate does not apply.

Table S30a: Study bias Characteristics for childhood sexual abuse

Author	Health outcome	sex_uncontrolled	regular_symptoms	exp_before_age above15	exp_before_age below15	women_only	selection_bias	age_uncontrolled	selfreported	type_nonsp	prevalent_diabetes	restricted_perp	reverse_causation	subpop	odds_ratio
Borges 2021	Alcohol use disorders	N/A	N/A	N/A	N/A	0	1	N/A	N/A	N/A	N/A	N/A	N/A	0	0
Cutajar 2010	Alcohol use disorders	N/A	N/A	N/A	N/A	0	0	N/A	N/A	N/A	N/A	N/A	N/A	0	1
Dinwiddie 2000	Alcohol use disorders	N/A	N/A	N/A	N/A	0	0	N/A	N/A	N/A	N/A	N/A	N/A	1	1
Fenton 2013	Alcohol use disorders	N/A	N/A	N/A	N/A	0	1	N/A	N/A	N/A	N/A	N/A	N/A	0	1
Fergusson 1996	Alcohol use disorders	N/A	N/A	N/A	N/A	0	0	N/A	N/A	N/A	N/A	N/A	N/A	0	1
Kendler 2000	Alcohol use disorders	N/A	N/A	N/A	N/A	1	1	N/A	N/A	N/A	N/A	N/A	N/A	1	1
Najman 2022	Alcohol use disorders	N/A	N/A	N/A	N/A	0	1	N/A	N/A	N/A	N/A	N/A	N/A	0	1
Nelson 2002	Alcohol use disorders	N/A	N/A	N/A	N/A	0	0	N/A	N/A	N/A	N/A	N/A	N/A	1	0
Roustit 2009	Alcohol use disorders	N/A	N/A	N/A	N/A	0	1	N/A	N/A	N/A	N/A	N/A	N/A	0	1
Sartor 2007	Alcohol use disorders	N/A	N/A	N/A	N/A	1	1	N/A	N/A	N/A	N/A	N/A	N/A	1	1
Laporte 2001	Anorexia nervosa	N/A	N/A	N/A	N/A	N/A	N/A	N/A	N/A	N/A	N/A	1	1	N/A	N/A
Paraventi 2011	Anorexia nervosa	N/A	N/A	N/A	N/A	N/A	N/A	N/A	N/A	N/A	N/A	0	1	N/A	N/A
Sanci 2008	Anorexia nervosa	N/A	N/A	N/A	N/A	N/A	N/A	N/A	N/A	N/A	N/A	1	0	N/A	N/A

Author	Health outcome	sex_uncontrolled	regular_symptoms	exp_before_age above15	exp_before_age below15	women_only	selection_bias	age_uncontrolled	selfreported	type_nonsp	prevalent_diabetes	restricted_perp	reverse_causation	subpop	odds_ratio
Talmon 2021	Anorexia nervosa	N/A	N/A	N/A	N/A	N/A	N/A	N/A	N/A	N/A	N/A	0	0	N/A	N/A
Cohen 2001	Anxiety disorders	N/A	N/A	1	0	0	0	0	N/A	N/A	N/A	N/A	N/A	N/A	N/A
Copeland 2018	Anxiety disorders	N/A	N/A	1	0	0	0	1	N/A	N/A	N/A	N/A	N/A	N/A	N/A
Dinwiddie 2000	Anxiety disorders	N/A	N/A	1	0	1	0	0	N/A	N/A	N/A	N/A	N/A	N/A	N/A
Fergusson 2008	Anxiety disorders	N/A	N/A	1	0	0	0	0	N/A	N/A	N/A	N/A	N/A	N/A	N/A
Fujiwara 2011	Anxiety disorders	N/A	N/A	1	0	0	1	0	N/A	N/A	N/A	N/A	N/A	N/A	N/A
Hovens 2015	Anxiety disorders	N/A	N/A	1	0	0	0	0	N/A	N/A	N/A	N/A	N/A	N/A	N/A
Kendler 2000	Anxiety disorders	N/A	N/A	1	0	1	1	0	N/A	N/A	N/A	N/A	N/A	N/A	N/A
Kisely 2021	Anxiety disorders	N/A	N/A	1	0	0	1	0	N/A	N/A	N/A	N/A	N/A	N/A	N/A
Raposo 2014	Anxiety disorders	N/A	N/A	1	0	0	0	1	N/A	N/A	N/A	N/A	N/A	N/A	N/A
Tenhave 2019	Anxiety disorders	N/A	N/A	1	0	0	1	0	N/A	N/A	N/A	N/A	N/A	N/A	N/A
Widom 1999	Anxiety disorders	N/A	N/A	0	1	0	1	0	N/A	N/A	N/A	N/A	N/A	N/A	N/A
Zinzow 2012	Anxiety disorders	N/A	N/A	0	1	1	1	0	N/A	N/A	N/A	N/A	N/A	N/A	N/A
Abajobir 2017	Asthma	1	0	0	1	N/A	N/A	N/A	N/A	N/A	N/A	N/A	N/A	N/A	N/A
Coogan 2013	Asthma	0	1	0	1	N/A	N/A	N/A	N/A	N/A	N/A	N/A	N/A	N/A	N/A
Han 2022	Asthma	0	1	1	0	N/A	N/A	N/A	N/A	N/A	N/A	N/A	N/A	N/A	N/A
Kascakova 2022	Asthma	1	0	1	0	N/A	N/A	N/A	N/A	N/A	N/A	N/A	N/A	N/A	N/A
Andrews 1995	Bulimia nervosa	N/A	N/A	N/A	N/A	N/A	1	1	N/A	N/A	N/A	N/A	N/A	N/A	N/A

Author	Health outcome	sex_uncontrolled	regular_symptoms	exp_before_age above15	exp_before_age below15	women_only	selection_bias	age_uncontrolled	selfreported	type_nonsp	prevalent_diabetes	restricted_perp	reverse_causation	subpop	odds_ratio
Kendler 2000	Bulimia nervosa	N/A	N/A	N/A	N/A	N/A	1	0	N/A	N/A	N/A	N/A	N/A	N/A	N/A
Paraventi 2011	Bulimia nervosa	N/A	N/A	N/A	N/A	N/A	0	1	N/A	N/A	N/A	N/A	N/A	N/A	N/A
Sanci 2008	Bulimia nervosa	N/A	N/A	N/A	N/A	N/A	1	0	N/A	N/A	N/A	N/A	N/A	N/A	N/A
Talmon 2021	Bulimia nervosa	N/A	N/A	N/A	N/A	N/A	0	0	N/A	N/A	N/A	N/A	N/A	N/A	N/A
Dinwiddie 2000	Conduct disorder	N/A	N/A	N/A	N/A	N/A	N/A	N/A	N/A	N/A	N/A	N/A	N/A	N/A	N/A
Fergusson 1996	Conduct disorder	N/A	N/A	N/A	N/A	N/A	N/A	N/A	N/A	N/A	N/A	N/A	N/A	N/A	N/A
Nelson 2002	Conduct disorder	N/A	N/A	N/A	N/A	N/A	N/A	N/A	N/A	N/A	N/A	N/A	N/A	N/A	N/A
Borges 2021	Drug use disorders	N/A	N/A	1	0	0	1	1	N/A	N/A	N/A	0	0	0	0
Cohen 2001	Drug use disorders	N/A	N/A	1	0	0	0	0	N/A	N/A	N/A	1	0	0	1
Conroy 2009	Drug use disorders	N/A	N/A	1	0	0	0	0	N/A	N/A	N/A	0	1	1	1
Cutajar 2010	Drug use disorders	N/A	N/A	1	0	0	0	0	N/A	N/A	N/A	0	1	0	1
Duncan 2008	Drug use disorders	N/A	N/A	1	0	0	1	0	N/A	N/A	N/A	0	0	1	0
Fergusson 1996	Drug use disorders	N/A	N/A	1	0	0	0	0	N/A	N/A	N/A	0	0	0	1
Huang 2011	Drug use disorders	N/A	N/A	0	1	0	1	0	N/A	N/A	N/A	1	0	0	1
Kalichman 2001	Drug use disorders	N/A	N/A	1	0	0	0	1	N/A	N/A	N/A	0	0	1	1
Kaukinen 2005	Drug use disorders	N/A	N/A	0	1	1	1	0	N/A	N/A	N/A	0	0	1	1
Kendler 2000	Drug use disorders	N/A	N/A	1	0	1	1	0	N/A	N/A	N/A	1	0	1	1
Najman 2022	Drug use disorders	N/A	N/A	1	0	0	1	0	N/A	N/A	N/A	0	0	0	1

Author	Health outcome	sex_uncontrolled	regular_symptoms	exp_before_age above15	exp_before_age below15	women_only	selection_bias	age_uncontrolled	selfreported	type_nonsp	prevalent_diabetes	restricted_perp	reverse_causation	subpop	odds_ratio
Nelson 2006	Drug use disorders	N/A	N/A	1	0	0	0	0	N/A	N/A	N/A	0	0	1	0
Nelson 2006	Drug use disorders	N/A	N/A	1	0	0	0	0	N/A	N/A	N/A	0	0	1	0
Sweet 2013	Drug use disorders	N/A	N/A	1	0	0	1	1	N/A	N/A	N/A	0	0	0	1
Sweet 2013	Drug use disorders	N/A	N/A	1	0	0	1	0	N/A	N/A	N/A	0	0	0	1
Tanaka 2015	Drug use disorders	N/A	N/A	1	0	0	1	0	N/A	N/A	N/A	0	0	0	1
Tonmyr 2017	Drug use disorders	N/A	N/A	1	0	0	0	0	N/A	N/A	N/A	0	0	0	1
Zinzow 2012	Drug use disorders	N/A	N/A	0	1	1	1	0	N/A	N/A	N/A	0	0	1	1
Deyessa 2018	HIV/AIDS	N/A	N/A	1	0	1	0	N/A	N/A	N/A	N/A	N/A	1	1	N/A
Maman 2002	HIV/AIDS	N/A	N/A	0	1	1	1	N/A	N/A	N/A	N/A	N/A	0	1	N/A
Mimiaga 2009	HIV/AIDS	N/A	N/A	1	0	0	0	N/A	N/A	N/A	N/A	N/A	0	1	N/A
Naicker 2022	HIV/AIDS	N/A	N/A	1	0	0	1	N/A	N/A	N/A	N/A	N/A	0	0	N/A
Widom 2012	HIV/AIDS	N/A	N/A	0	1	0	1	N/A	N/A	N/A	N/A	N/A	0	0	N/A
Wyatt 2002	HIV/AIDS	N/A	N/A	1	0	1	0	N/A	N/A	N/A	N/A	N/A	1	1	N/A
Xavierhall 2021	HIV/AIDS	N/A	N/A	0	1	0	0	N/A	N/A	N/A	N/A	N/A	0	1	N/A
Dong 2004	Ischemic heart disease	N/A	N/A	N/A	N/A	N/A	N/A	N/A	N/A	N/A	N/A	N/A	N/A	N/A	N/A
Kascakova 2022	Ischemic heart disease	N/A	N/A	N/A	N/A	N/A	N/A	N/A	N/A	N/A	N/A	N/A	N/A	N/A	N/A
Monnat 2015	Ischemic heart disease	N/A	N/A	N/A	N/A	N/A	N/A	N/A	N/A	N/A	N/A	N/A	N/A	N/A	N/A
Andrews 1995	Major depressive disorder	0	N/A	1	0	1	1	1	N/A	N/A	N/A	N/A	0	1	1
Bifulco 1991	Major depressive disorder	0	N/A	1	0	1	1	1	N/A	N/A	N/A	N/A	0	1	1

Author	Health outcome	sex_uncontrolled	regular_symptoms	exp_before_age above15	exp_before_age below15	women_only	selection_bias	age_uncontrolled	selfreported	type_nonsp	prevalent_diabetes	restricted_perp	reverse_causation	subpop	odds_ratio
Chapman 2004	Major depressive disorder	0	N/A	1	0	0	1	1	N/A	N/A	N/A	N/A	0	1	1
Cheasty 1998	Major depressive disorder	0	N/A	1	0	1	1	1	N/A	N/A	N/A	N/A	1	1	1
Cohen 2001	Major depressive disorder	0	N/A	1	0	0	0	0	N/A	N/A	N/A	N/A	0	0	1
Comijs 2013	Major depressive disorder	0	N/A	1	0	0	0	0	N/A	N/A	N/A	N/A	1	0	1
Copeland 2018	Major depressive disorder	0	N/A	1	0	0	0	1	N/A	N/A	N/A	N/A	0	0	1
Dinwiddie 2000	Major depressive disorder	0	N/A	1	0	0	0	0	N/A	N/A	N/A	N/A	0	0	1
Dube 2005	Major depressive disorder	0	N/A	1	0	0	0	0	N/A	N/A	N/A	N/A	0	1	1
Ebert 2019	Major depressive disorder	1	N/A	1	0	0	1	1	N/A	N/A	N/A	N/A	0	1	1
Fergusson 2008	Major depressive disorder	0	N/A	1	0	0	0	0	N/A	N/A	N/A	N/A	0	0	1
Galloeag 2017	Major depressive disorder	0	N/A	0	0	1	1	0	N/A	N/A	N/A	N/A	0	1	1
Houtepen 2020	Major depressive disorder	0	N/A	0	0	0	0	0	N/A	N/A	N/A	N/A	0	0	1
Hovens 2015	Major depressive disorder	0	N/A	1	0	0	0	0	N/A	N/A	N/A	N/A	0	0	1
Jaffee 2002	Major depressive disorder	0	N/A	0	1	0	0	1	N/A	N/A	N/A	N/A	0	0	1
Kendler 2000	Major depressive disorder	0	N/A	1	0	1	1	0	N/A	N/A	N/A	N/A	0	1	1
Kisely 2021	Major depressive disorder	0	N/A	1	0	0	1	0	N/A	N/A	N/A	N/A	0	0	1
Mullen 1996	Major depressive disorder	0	N/A	1	0	1	1	1	N/A	N/A	N/A	N/A	0	1	1

Author	Health outcome	sex_uncontrolled	regular_symptoms	exp_before_age above15	exp_before_age below15	women_only	selection_bias	age_uncontrolled	selfreported	type_nonsp	prevalent_diabetes	restricted_perp	reverse_causation	subpop	odds_ratio
Nelson 2002	Major depressive disorder	0	N/A	1	0	0	0	0	N/A	N/A	N/A	N/A	0	0	0
Ratner 2003	Major depressive disorder	0	N/A	0	1	0	0	1	N/A	N/A	N/A	N/A	0	1	1
Roustit 2009	Major depressive disorder	0	N/A	1	0	0	1	0	N/A	N/A	N/A	N/A	0	0	1
Su 2022	Major depressive disorder	1	N/A	1	0	0	1	1	N/A	N/A	N/A	N/A	0	0	0
Widom 2007	Major depressive disorder	0	N/A	0	1	0	1	0	N/A	N/A	N/A	N/A	0	0	1
Wise 2001	Major depressive disorder	0	N/A	1	0	1	0	0	N/A	N/A	N/A	N/A	1	1	0
Xiao 2022	Major depressive disorder	1	N/A	0	0	0	0	1	N/A	N/A	N/A	N/A	1	0	1
Zinzow 2012	Major depressive disorder	0	N/A	0	1	1	1	0	N/A	N/A	N/A	N/A	0	1	1
Abajobir 2018	Maternal abortion and miscarriage	N/A	N/A	N/A	N/A	N/A	1	N/A	N/A	N/A	N/A	N/A	N/A	N/A	1
Demakakos 2020	Maternal abortion and miscarriage	N/A	N/A	N/A	N/A	N/A	0	N/A	N/A	N/A	N/A	N/A	N/A	N/A	0
Fortin-Langelier 2019	Maternal abortion and miscarriage	N/A	N/A	N/A	N/A	N/A	1	N/A	N/A	N/A	N/A	N/A	N/A	N/A	0
Kerkar 2021	Maternal abortion and miscarriage	N/A	N/A	N/A	N/A	N/A	0	N/A	N/A	N/A	N/A	N/A	N/A	N/A	1
Taft 2019	Maternal abortion and miscarriage	N/A	N/A	N/A	N/A	N/A	1	N/A	N/A	N/A	N/A	N/A	N/A	N/A	1
Vanroode 2009	Maternal abortion and miscarriage	N/A	N/A	N/A	N/A	N/A	1	N/A	N/A	N/A	N/A	N/A	N/A	N/A	0
Chatziioannidis 2019	Schizophrenia	N/A	N/A	1	N/A	N/A	N/A	N/A	N/A	N/A	N/A	N/A	N/A	N/A	N/A
Cutajar 2010	Schizophrenia	N/A	N/A	1	N/A	N/A	N/A	N/A	N/A	N/A	N/A	N/A	N/A	N/A	N/A

Author	Health outcome	sex_uncontrolled	regular_symptoms	exp_before_age above15	exp_before_age below15	women_only	selection_bias	age_uncontrolled	selfreported	type_nonsp	prevalent_diabetes	restricted_perp	reverse_causation	subpop	odds_ratio
Mall 2020	Schizophrenia	N/A	N/A	1	N/A	N/A	N/A	N/A	N/A	N/A	N/A	N/A	N/A	N/A	N/A
Mansueto 2022	Schizophrenia	N/A	N/A	0	N/A	N/A	N/A	N/A	N/A	N/A	N/A	N/A	N/A	N/A	N/A
Murphy 2020	Schizophrenia	N/A	N/A	0	N/A	N/A	N/A	N/A	N/A	N/A	N/A	N/A	N/A	N/A	N/A
Banyard 2004	Self-harm	N/A	N/A	1	N/A	0	1	1	N/A	N/A	N/A	N/A	0	1	1
Bentivegna 2022	Self-harm	N/A	N/A	1	N/A	0	0	0	N/A	N/A	N/A	N/A	0	0	0
Borges 2021	Self-harm	N/A	N/A	1	N/A	0	1	1	N/A	N/A	N/A	N/A	0	0	0
Brown 1999	Self-harm	N/A	N/A	1	N/A	0	0	0	N/A	N/A	N/A	N/A	0	0	1
Chen 2014	Self-harm	N/A	N/A	1	N/A	1	0	1	N/A	N/A	N/A	N/A	1	1	1
Dinwiddie 2000	Self-harm	N/A	N/A	1	N/A	1	0	0	N/A	N/A	N/A	N/A	0	1	1
Dube 2005	Self-harm	N/A	N/A	1	N/A	0	0	0	N/A	N/A	N/A	N/A	0	1	1
Enns 2006	Self-harm	N/A	N/A	1	N/A	0	1	0	N/A	N/A	N/A	N/A	0	0	1
Guiney 2022	Self-harm	N/A	N/A	1	N/A	0	0	1	N/A	N/A	N/A	N/A	0	0	0
Kiselydmedres 2022	Self-harm	N/A	N/A	0	N/A	0	1	0	N/A	N/A	N/A	N/A	0	0	1
Mullen 1996	Self-harm	N/A	N/A	1	N/A	1	1	1	N/A	N/A	N/A	N/A	0	1	1
Nelson 2002	Self-harm	N/A	N/A	1	N/A	0	0	0	N/A	N/A	N/A	N/A	0	1	0
Rajapakse 2020	Self-harm	N/A	N/A	1	N/A	0	0	0	N/A	N/A	N/A	N/A	1	1	1
Roustit 2009	Self-harm	N/A	N/A	1	N/A	0	1	0	N/A	N/A	N/A	N/A	0	0	1
Thompson 2019	Self-harm	N/A	N/A	1	N/A	0	1	0	N/A	N/A	N/A	N/A	0	0	1
Xavierhall 2021	Self-harm	N/A	N/A	0	N/A	0	0	0	N/A	N/A	N/A	N/A	0	1	1

Author	Health outcome	sex_uncontrolled	regular_symptoms	exp_before_age above15	exp_before_age below15	women_only	selection_bias	age_uncontrolled	selfreported	type_nonsp	prevalent_diabetes	restricted_perp	reverse_causation	subpop	odds_ratio
Guiney 2022	Sexually transmitted infections excluding HIV	N/A	N/A	1	N/A	N/A	0	1	N/A	N/A	N/A	0	N/A	N/A	N/A
Haydon 2011	Sexually transmitted infections excluding HIV	N/A	N/A	1	N/A	N/A	1	0	N/A	N/A	N/A	1	N/A	N/A	N/A
Wilson 2009	Sexually transmitted infections excluding HIV	N/A	N/A	0	N/A	N/A	1	1	N/A	N/A	N/A	1	N/A	N/A	N/A
Wilson 2009	Sexually transmitted infections excluding HIV	N/A	N/A	0	N/A	N/A	1	1	N/A	N/A	N/A	0	N/A	N/A	N/A
Xavierhall 2021	Sexually transmitted infections excluding HIV	N/A	N/A	0	N/A	N/A	0	0	N/A	N/A	N/A	0	N/A	N/A	N/A
Duncan 2015	Type 2 diabetes mellitus	N/A	N/A	N/A	N/A	0	0	0	0	1	1	1	N/A	N/A	N/A
Kascakova 2022	Type 2 diabetes mellitus	N/A	N/A	N/A	N/A	0	0	1	1	1	1	0	N/A	N/A	N/A
Monnat 2015	Type 2 diabetes mellitus	N/A	N/A	N/A	N/A	0	0	0	1	1	1	0	N/A	N/A	N/A
Rich-Edwards 2010	Type 2 diabetes mellitus	N/A	N/A	N/A	N/A	1	1	0	1	0	0	0	N/A	N/A	N/A
Seid 2022	Type 2 diabetes mellitus	N/A	N/A	N/A	N/A	1	0	1	1	1	0	0	N/A	N/A	N/A
Shields 2016	Type 2 diabetes mellitus	N/A	N/A	N/A	N/A	0	0	0	1	1	1	0	N/A	N/A	N/A
Thomas 2008	Type 2 diabetes mellitus	N/A	N/A	N/A	N/A	0	1	0	0	0	0	1	N/A	N/A	N/A

N/A, not available across minimum number of studies needed and not tested for given risk outcome pair. 1 = bias covariate applies to the study; 0 = bias covariate does not apply.

Table S30b: Study bias Characteristics for childhood sexual abuse (continued)

Author	Health outcome	repres entativ eness	confounding_u ncontrolled	lifetime_at tempts	L1	admin_ exp_me thod	alternate_out comedef	component_o utcomedef	use	specific _substa nce	cann abis	component_ outcome	symptom _scale	lifetime_di agnosis	aggregate_ou tcomedef
Borges 2021	Alcohol use disorders	0	N/A	N/A	1	N/A	1	N/A	N/A	N/A	N/A	N/A	N/A	N/A	N/A
Cutajar 2010	Alcohol use disorders	1	N/A	N/A	0	N/A	1	N/A	N/A	N/A	N/A	N/A	N/A	N/A	N/A
Dinwiddi e 2000	Alcohol use disorders	0	N/A	N/A	0	N/A	0	N/A	N/A	N/A	N/A	N/A	N/A	N/A	N/A

Author	Health outcome	representativeness	confounding_uncontrolled	lifetime_attempts	L1	admin_exp_method	alternate_outcomedef	component_outcomedef	use	specific_substance	cannabis	component_outcome	symptom_scale	lifetime_diagnosis	aggregate_outcomedef
Fenton 2013	Alcohol use disorders	0	N/A	N/A	0	N/A	0	N/A	N/A	N/A	N/A	N/A	N/A	N/A	N/A
Fergusson 1996	Alcohol use disorders	1	N/A	N/A	0	N/A	1	N/A	N/A	N/A	N/A	N/A	N/A	N/A	N/A
Kendler 2000	Alcohol use disorders	1	N/A	N/A	0	N/A	0	N/A	N/A	N/A	N/A	N/A	N/A	N/A	N/A
Najman 2022	Alcohol use disorders	0	N/A	N/A	1	N/A	1	N/A	N/A	N/A	N/A	N/A	N/A	N/A	N/A
Nelson 2002	Alcohol use disorders	1	N/A	N/A	0	N/A	0	N/A	N/A	N/A	N/A	N/A	N/A	N/A	N/A
Roustit 2009	Alcohol use disorders	1	N/A	N/A	0	N/A	0	N/A	N/A	N/A	N/A	N/A	N/A	N/A	N/A
Sartor 2007	Alcohol use disorders	1	N/A	N/A	0	N/A	0	N/A	N/A	N/A	N/A	N/A	N/A	N/A	N/A
Laporte 2001	Anorexia nervosa	N/A	N/A	N/A	N/A	N/A	N/A	N/A	N/A	N/A	N/A	N/A	N/A	N/A	N/A
Paraventi 2011	Anorexia nervosa	N/A	N/A	N/A	N/A	N/A	N/A	N/A	N/A	N/A	N/A	N/A	N/A	N/A	N/A
Sanci 2008	Anorexia nervosa	N/A	N/A	N/A	N/A	N/A	N/A	N/A	N/A	N/A	N/A	N/A	N/A	N/A	N/A
Talmon 2021	Anorexia nervosa	N/A	N/A	N/A	N/A	N/A	N/A	N/A	N/A	N/A	N/A	N/A	N/A	N/A	N/A
Cohen 2001	Anxiety disorders	0	N/A	N/A	1	N/A	N/A	0	N/A	N/A	N/A	N/A	N/A	N/A	N/A
Copeland 2018	Anxiety disorders	0	N/A	N/A	1	N/A	N/A	0	N/A	N/A	N/A	N/A	N/A	N/A	N/A
Dinwiddie 2000	Anxiety disorders	0	N/A	N/A	1	N/A	N/A	1	N/A	N/A	N/A	N/A	N/A	N/A	N/A
Fergusson 2008	Anxiety disorders	0	N/A	N/A	0	N/A	N/A	0	N/A	N/A	N/A	N/A	N/A	N/A	N/A
Fujiwara 2011	Anxiety disorders	1	N/A	N/A	0	N/A	N/A	0	N/A	N/A	N/A	N/A	N/A	N/A	N/A
Hovens 2015	Anxiety disorders	1	N/A	N/A	0	N/A	N/A	0	N/A	N/A	N/A	N/A	N/A	N/A	N/A
Kendler 2000	Anxiety disorders	1	N/A	N/A	0	N/A	N/A	1	N/A	N/A	N/A	N/A	N/A	N/A	N/A
Kisely 2021	Anxiety disorders	0	N/A	N/A	0	N/A	N/A	0	N/A	N/A	N/A	N/A	N/A	N/A	N/A

Author	Health outcome	representativeness	confounding_uncontrolled	lifetime_attempts	L1	admin_exp_method	alternate_outcomedef	component_outcomedef	use	specific_substance	cannabis	component_outcome	symptom_scale	lifetime_diagnosis	aggregate_outcomedef
Raposo 2014	Anxiety disorders	0	N/A	N/A	1	N/A	N/A	0	N/A	N/A	N/A	N/A	N/A	N/A	N/A
Tenhave 2019	Anxiety disorders	0	N/A	N/A	1	N/A	N/A	0	N/A	N/A	N/A	N/A	N/A	N/A	N/A
Widom 1999	Anxiety disorders	1	N/A	N/A	1	N/A	N/A	1	N/A	N/A	N/A	N/A	N/A	N/A	N/A
Zinzow 2012	Anxiety disorders	1	N/A	N/A	0	N/A	N/A	1	N/A	N/A	N/A	N/A	N/A	N/A	N/A
Abajobir 2017	Asthma	N/A	N/A	N/A	N/A	N/A	N/A	N/A	N/A	N/A	N/A	N/A	N/A	N/A	N/A
Coogan 2013	Asthma	N/A	N/A	N/A	N/A	N/A	N/A	N/A	N/A	N/A	N/A	N/A	N/A	N/A	N/A
Han 2022	Asthma	N/A	N/A	N/A	N/A	N/A	N/A	N/A	N/A	N/A	N/A	N/A	N/A	N/A	N/A
Kascakova 2022	Asthma	N/A	N/A	N/A	N/A	N/A	N/A	N/A	N/A	N/A	N/A	N/A	N/A	N/A	N/A
Andrews 1995	Bulimia nervosa	N/A	1	N/A	N/A	N/A	N/A	N/A	N/A	N/A	N/A	1	N/A	N/A	N/A
Kendler 2000	Bulimia nervosa	N/A	0	N/A	N/A	N/A	N/A	N/A	N/A	N/A	N/A	1	N/A	N/A	N/A
Paraventi 2011	Bulimia nervosa	N/A	1	N/A	N/A	N/A	N/A	N/A	N/A	N/A	N/A	0	N/A	N/A	N/A
Sanci 2008	Bulimia nervosa	N/A	1	N/A	N/A	N/A	N/A	N/A	N/A	N/A	N/A	0	N/A	N/A	N/A
Talmon 2021	Bulimia nervosa	N/A	0	N/A	N/A	N/A	N/A	N/A	N/A	N/A	N/A	1	N/A	N/A	N/A
Dinwiddie 2000	Conduct disorder	N/A	N/A	N/A	N/A	N/A	N/A	N/A	N/A	N/A	N/A	N/A	N/A	N/A	N/A
Fergusson 1996	Conduct disorder	N/A	N/A	N/A	N/A	N/A	N/A	N/A	N/A	N/A	N/A	N/A	N/A	N/A	N/A
Nelson 2002	Conduct disorder	N/A	N/A	N/A	N/A	N/A	N/A	N/A	N/A	N/A	N/A	N/A	N/A	N/A	N/A
Borges 2021	Drug use disorders	0	0	N/A	1	N/A	N/A	N/A	0	0	0	N/A	N/A	N/A	N/A
Cohen 2001	Drug use disorders	0	0	N/A	1	N/A	N/A	N/A	0	0	0	N/A	N/A	N/A	N/A
Conroy 2009	Drug use disorders	1	0	N/A	0	N/A	N/A	N/A	0	1	0	N/A	N/A	N/A	N/A

Author	Health outcome	representativeness	confounding_uncontrolled	lifetime_attempts	L1	admin_exp_method	alternate_outcomedef	component_outcomedef	use	specific_substance	cannabis	component_outcome	symptom_scale	lifetime_diagnosis	aggregate_outcomedef
Cutajar 2010	Drug use disorders	1	0	N/A	1	N/A	N/A	N/A	0	0	0	N/A	N/A	N/A	N/A
Duncan 2008	Drug use disorders	1	0	N/A	0	N/A	N/A	N/A	0	1	1	N/A	N/A	N/A	N/A
Fergusson 1996	Drug use disorders	1	0	N/A	0	N/A	N/A	N/A	0	0	0	N/A	N/A	N/A	N/A
Huang 2011	Drug use disorders	0	0	N/A	0	N/A	N/A	N/A	1	0	0	N/A	N/A	N/A	N/A
Kalichman 2001	Drug use disorders	1	1	N/A	1	N/A	N/A	N/A	1	0	0	N/A	N/A	N/A	N/A
Kaukinen 2005	Drug use disorders	1	0	N/A	0	N/A	N/A	N/A	1	0	0	N/A	N/A	N/A	N/A
Kendler 2000	Drug use disorders	1	0	N/A	0	N/A	N/A	N/A	0	0	0	N/A	N/A	N/A	N/A
Najman 2022	Drug use disorders	0	0	N/A	1	N/A	N/A	N/A	0	0	0	N/A	N/A	N/A	N/A
Nelson 2006	Drug use disorders	0	0	N/A	1	N/A	N/A	N/A	1	1	1	N/A	N/A	N/A	N/A
Nelson 2006	Drug use disorders	0	0	N/A	1	N/A	N/A	N/A	1	1	0	N/A	N/A	N/A	N/A
Sweet 2013	Drug use disorders	0	1	N/A	1	N/A	N/A	N/A	0	0	0	N/A	N/A	N/A	N/A
Sweet 2013	Drug use disorders	0	0	N/A	1	N/A	N/A	N/A	0	0	0	N/A	N/A	N/A	N/A
Tanaka 2015	Drug use disorders	0	0	N/A	0	N/A	N/A	N/A	1	0	0	N/A	N/A	N/A	N/A
Tonmyr 2017	Drug use disorders	1	0	N/A	0	N/A	N/A	N/A	1	0	0	N/A	N/A	N/A	N/A
Zinzow 2012	Drug use disorders	1	0	N/A	0	N/A	N/A	N/A	0	0	0	N/A	N/A	N/A	N/A
Deyessa 2018	HIV/AIDS	N/A	N/A	N/A	N/A	N/A	N/A	N/A	N/A	N/A	N/A	N/A	N/A	N/A	N/A
Maman 2002	HIV/AIDS	N/A	N/A	N/A	N/A	N/A	N/A	N/A	N/A	N/A	N/A	N/A	N/A	N/A	N/A
Mimiaga 2009	HIV/AIDS	N/A	N/A	N/A	N/A	N/A	N/A	N/A	N/A	N/A	N/A	N/A	N/A	N/A	N/A
Naicker 2022	HIV/AIDS	N/A	N/A	N/A	N/A	N/A	N/A	N/A	N/A	N/A	N/A	N/A	N/A	N/A	N/A
Widom 2012	HIV/AIDS	N/A	N/A	N/A	N/A	N/A	N/A	N/A	N/A	N/A	N/A	N/A	N/A	N/A	N/A

Author	Health outcome	representativeness	confounding_uncontrolled	lifetime_attempts	L1	admin_exp_method	alternate_outcomedef	component_outcomedef	use	specific_substance	cannabis	component_outcome	symptom_scale	lifetime_diagnosis	aggregate_outcomedef
Wyatt 2002	HIV/AIDS	N/A	N/A	N/A	N/A	N/A	N/A	N/A	N/A	N/A	N/A	N/A	N/A	N/A	N/A
Xavierhall 2021	HIV/AIDS	N/A	N/A	N/A	N/A	N/A	N/A	N/A	N/A	N/A	N/A	N/A	N/A	N/A	N/A
Dong 2004	Ischemic heart disease	N/A	N/A	N/A	N/A	N/A	N/A	N/A	N/A	N/A	N/A	N/A	N/A	N/A	N/A
Kascakova 2022	Ischemic heart disease	N/A	N/A	N/A	N/A	N/A	N/A	N/A	N/A	N/A	N/A	N/A	N/A	N/A	N/A
Monnat 2015	Ischemic heart disease	N/A	N/A	N/A	N/A	N/A	N/A	N/A	N/A	N/A	N/A	N/A	N/A	N/A	N/A
Andrews 1995	Major depressive disorder	1	1	N/A	1	N/A	N/A	N/A	N/A	N/A	N/A	N/A	0	0	1
Bifulco 1991	Major depressive disorder	1	1	N/A	1	N/A	N/A	N/A	N/A	N/A	N/A	N/A	0	0	1
Chapman 2004	Major depressive disorder	1	0	N/A	1	N/A	N/A	N/A	N/A	N/A	N/A	N/A	1	0	1
Cheasty 1998	Major depressive disorder	1	1	N/A	1	N/A	N/A	N/A	N/A	N/A	N/A	N/A	1	0	0
Cohen 2001	Major depressive disorder	0	0	N/A	1	N/A	N/A	N/A	N/A	N/A	N/A	N/A	0	0	1
Comijs 2013	Major depressive disorder	1	0	N/A	1	N/A	N/A	N/A	N/A	N/A	N/A	N/A	0	0	1
Copeland 2018	Major depressive disorder	0	0	N/A	1	N/A	N/A	N/A	N/A	N/A	N/A	N/A	0	0	1
Dinwiddie 2000	Major depressive disorder	0	0	N/A	1	N/A	N/A	N/A	N/A	N/A	N/A	N/A	0	1	0
Dube 2005	Major depressive disorder	1	0	N/A	0	N/A	N/A	N/A	N/A	N/A	N/A	N/A	0	0	1
Ebert 2019	Major depressive disorder	1	0	N/A	1	N/A	N/A	N/A	N/A	N/A	N/A	N/A	0	0	0
Fergusson 2008	Major depressive disorder	0	0	N/A	0	N/A	N/A	N/A	N/A	N/A	N/A	N/A	0	0	0

Author	Health outcome	representativeness	confounding_uncontrolled	lifetime_attempts	L1	admin_exp_method	alternate_outcomedef	component_outcomedef	use	specific_substance	cannabis	component_outcome	symptom_scale	lifetime_diagnosis	aggregate_outcomedef
Galloeag 2017	Major depressive disorder	0	0	N/A	0	N/A	N/A	N/A	N/A	N/A	N/A	N/A	0	0	0
Houtepen 2020	Major depressive disorder	0	0	N/A	0	N/A	N/A	N/A	N/A	N/A	N/A	N/A	0	0	1
Hovens 2015	Major depressive disorder	1	0	N/A	0	N/A	N/A	N/A	N/A	N/A	N/A	N/A	0	0	1
Jaffee 2002	Major depressive disorder	1	0	N/A	1	N/A	N/A	N/A	N/A	N/A	N/A	N/A	0	0	0
Kendler 2000	Major depressive disorder	1	0	N/A	0	N/A	N/A	N/A	N/A	N/A	N/A	N/A	0	0	0
Kisely 2021	Major depressive disorder	0	0	N/A	0	N/A	N/A	N/A	N/A	N/A	N/A	N/A	0	0	1
Mullen 1996	Major depressive disorder	1	1	N/A	1	N/A	N/A	N/A	N/A	N/A	N/A	N/A	0	1	1
Nelson 2002	Major depressive disorder	1	0	N/A	1	N/A	N/A	N/A	N/A	N/A	N/A	N/A	0	0	0
Ratner 2003	Major depressive disorder	1	1	N/A	1	N/A	N/A	N/A	N/A	N/A	N/A	N/A	0	0	1
Roustit 2009	Major depressive disorder	1	0	N/A	0	N/A	N/A	N/A	N/A	N/A	N/A	N/A	0	0	1
Su 2022	Major depressive disorder	1	0	N/A	1	N/A	N/A	N/A	N/A	N/A	N/A	N/A	0	0	0
Widom 2007	Major depressive disorder	1	0	N/A	1	N/A	N/A	N/A	N/A	N/A	N/A	N/A	0	0	0
Wise 2001	Major depressive disorder	0	0	N/A	0	N/A	N/A	N/A	N/A	N/A	N/A	N/A	0	0	1
Xiao 2022	Major depressive disorder	0	1	N/A	1	N/A	N/A	N/A	N/A	N/A	N/A	N/A	0	0	1
Zinzow 2012	Major depressive disorder	1	0	N/A	0	N/A	N/A	N/A	N/A	N/A	N/A	N/A	0	0	0
Abajobir 2018	Maternal abortion and miscarriage	N/A	N/A	N/A	0	1	N/A	N/A	N/A	N/A	N/A	N/A	N/A	N/A	N/A

Author	Health outcome	representativeness	confounding_uncontrolled	lifetime_attempts	L1	admin_exp_method	alternate_outcomedef	component_outcomedef	use	specific_substance	cannabis	component_outcome	symptom_scale	lifetime_diagnosis	aggregate_outcomedef
Demakakos 2020	Maternal abortion and miscarriage	N/A	N/A	N/A	0	0	N/A	N/A	N/A	N/A	N/A	N/A	N/A	N/A	N/A
Fortin-Langelier 2019	Maternal abortion and miscarriage	N/A	N/A	N/A	1	1	N/A	N/A	N/A	N/A	N/A	N/A	N/A	N/A	N/A
Kerkar 2021	Maternal abortion and miscarriage	N/A	N/A	N/A	0	0	N/A	N/A	N/A	N/A	N/A	N/A	N/A	N/A	N/A
Taft 2019	Maternal abortion and miscarriage	N/A	N/A	N/A	0	0	N/A	N/A	N/A	N/A	N/A	N/A	N/A	N/A	N/A
Vanroode 2009	Maternal abortion and miscarriage	N/A	N/A	N/A	1	0	N/A	N/A	N/A	N/A	N/A	N/A	N/A	N/A	N/A
Chatziioannidis 2019	Schizophrenia	N/A	1	N/A	1	N/A	N/A	N/A	N/A	N/A	N/A	N/A	N/A	N/A	N/A
Cutajar 2010	Schizophrenia	N/A	0	N/A	1	N/A	N/A	N/A	N/A	N/A	N/A	N/A	N/A	N/A	N/A
Mall 2020	Schizophrenia	N/A	0	N/A	0	N/A	N/A	N/A	N/A	N/A	N/A	N/A	N/A	N/A	N/A
Mansueto 2022	Schizophrenia	N/A	0	N/A	0	N/A	N/A	N/A	N/A	N/A	N/A	N/A	N/A	N/A	N/A
Murphy 2020	Schizophrenia	N/A	1	N/A	1	N/A	N/A	N/A	N/A	N/A	N/A	N/A	N/A	N/A	N/A
Banyard 2004	Self-harm	1	0	1	1	N/A	N/A	N/A	N/A	N/A	N/A	N/A	N/A	N/A	N/A
Bentivegna 2022	Self-harm	0	0	0	0	N/A	N/A	N/A	N/A	N/A	N/A	N/A	N/A	N/A	N/A
Borges 2021	Self-harm	0	0	0	1	N/A	N/A	N/A	N/A	N/A	N/A	N/A	N/A	N/A	N/A
Brown 1999	Self-harm	1	0	0	0	N/A	N/A	N/A	N/A	N/A	N/A	N/A	N/A	N/A	N/A
Chen 2014	Self-harm	1	1	0	1	N/A	N/A	N/A	N/A	N/A	N/A	N/A	N/A	N/A	N/A
Dinwiddie 2000	Self-harm	0	0	1	1	N/A	N/A	N/A	N/A	N/A	N/A	N/A	N/A	N/A	N/A
Dube 2005	Self-harm	1	0	1	0	N/A	N/A	N/A	N/A	N/A	N/A	N/A	N/A	N/A	N/A
Enns 2006	Self-harm	0	0	0	0	N/A	N/A	N/A	N/A	N/A	N/A	N/A	N/A	N/A	N/A
Guiney 2022	Self-harm	0	0	0	1	N/A	N/A	N/A	N/A	N/A	N/A	N/A	N/A	N/A	N/A

Author	Health outcome	representativeness	confounding_uncontrolled	lifetime_attempts	L1	admin_exp_method	alternate_outcomedef	component_outcomedef	use	specific_substance	cannabis	component_outcome	symptom_scale	lifetime_diagnosis	aggregate_outcomedef
Kiselydmedres 2022	Self-harm	1	1	0	1	N/A	N/A	N/A	N/A	N/A	N/A	N/A	N/A	N/A	N/A
Mullen 1996	Self-harm	1	1	0	1	N/A	N/A	N/A	N/A	N/A	N/A	N/A	N/A	N/A	N/A
Nelson 2002	Self-harm	1	0	1	1	N/A	N/A	N/A	N/A	N/A	N/A	N/A	N/A	N/A	N/A
Rajapakse 2020	Self-harm	1	0	0	1	N/A	N/A	N/A	N/A	N/A	N/A	N/A	N/A	N/A	N/A
Roustit 2009	Self-harm	1	0	0	0	N/A	N/A	N/A	N/A	N/A	N/A	N/A	N/A	N/A	N/A
Thompson 2019	Self-harm	0	0	0	0	N/A	N/A	N/A	N/A	N/A	N/A	N/A	N/A	N/A	N/A
Xavierhall 2021	Self-harm	1	0	0	0	N/A	N/A	N/A	N/A	N/A	N/A	N/A	N/A	N/A	N/A
Guiney 2022	Sexually transmitted infections excluding HIV	0	N/A	N/A	N/A	N/A	N/A	N/A	N/A	N/A	N/A	N/A	N/A	N/A	N/A
Haydon 2011	Sexually transmitted infections excluding HIV	0	N/A	N/A	N/A	N/A	N/A	N/A	N/A	N/A	N/A	N/A	N/A	N/A	N/A
Wilson 2009	Sexually transmitted infections excluding HIV	1	N/A	N/A	N/A	N/A	N/A	N/A	N/A	N/A	N/A	N/A	N/A	N/A	N/A
Wilson 2009	Sexually transmitted infections excluding HIV	1	N/A	N/A	N/A	N/A	N/A	N/A	N/A	N/A	N/A	N/A	N/A	N/A	N/A
Xavierhall 2021	Sexually transmitted infections excluding HIV	1	N/A	N/A	N/A	N/A	N/A	N/A	N/A	N/A	N/A	N/A	N/A	N/A	N/A
Duncan 2015	Type 2 diabetes mellitus	N/A	N/A	N/A	N/A	N/A	N/A	N/A	N/A	N/A	N/A	N/A	N/A	N/A	N/A
Kascakova 2022	Type 2 diabetes mellitus	N/A	N/A	N/A	N/A	N/A	N/A	N/A	N/A	N/A	N/A	N/A	N/A	N/A	N/A

Author	Health outcome	representativeness	confounding_uncontrolled	lifetime_attempts	L1	admin_exp_method	alternate_outcomedef	component_outcomedef	use	specific_substance	cannabis	component_outcome	symptom_scale	lifetime_diagnosis	aggregate_outcomedef
Monnat 2015	Type 2 diabetes mellitus	N/A	N/A	N/A	N/A	N/A	N/A	N/A	N/A	N/A	N/A	N/A	N/A	N/A	N/A
Rich-Edwards 2010	Type 2 diabetes mellitus	N/A	N/A	N/A	N/A	N/A	N/A	N/A	N/A	N/A	N/A	N/A	N/A	N/A	N/A
Seid 2022	Type 2 diabetes mellitus	N/A	N/A	N/A	N/A	N/A	N/A	N/A	N/A	N/A	N/A	N/A	N/A	N/A	N/A
Shields 2016	Type 2 diabetes mellitus	N/A	N/A	N/A	N/A	N/A	N/A	N/A	N/A	N/A	N/A	N/A	N/A	N/A	N/A
Thomas 2008	Type 2 diabetes mellitus	N/A	N/A	N/A	N/A	N/A	N/A	N/A	N/A	N/A	N/A	N/A	N/A	N/A	N/A

N/A, not available across minimum number of studies needed and not tested for given risk outcome pair. 1 = bias covariate applies to the study; 0 = bias covariate does not apply.

Section 7: Calculating the ROS and measure of excess risk

Following the methods outlined by Zheng and colleagues, the burden of proof risk function is defined as the 5th quantile closest to the RR equal to one, interpretable as the smallest harmful effect of the risk factor consistent with available evidence. The BPRF is calculated by combining uncertainty of the mean, estimated between-study heterogeneity (γ), and the 95th quantile of γ obtained from the fisher information matrix. The risk-outcome score (ROS) summarizes the effect of the risk factor on the health outcome under study and is calculated as the absolute value of the log BRPF divided by two:

$$ROS = \frac{|\log{(BPRF)}|}{2}$$

ROS are categorized into star ratings according to the following thresholds: ROS (one-star, ≤ 0.0 ROS; two-star, > 0.0–0.14 ROS; three-star, > 0.14–0.41 ROS; four-star, > 0.41–0.62 ROS; five-star, > 0.62 ROS).

From the log BPRF, we can also calculate a measure of excess risk:

$$Minimum\ percent\ increase\ in\ risk = (BPRF - 1) * 100\%$$
